# Functional Nano-Objects by Electrostatic Self-Assembly: Structure, Switching, and Photocatalysis

**DOI:** 10.3389/fchem.2021.779360

**Published:** 2022-03-10

**Authors:** Anja Krieger, Alexander Zika, Franziska Gröhn

**Affiliations:** Department of Chemistry and Pharmacy and Interdisciplinary Center for Molecular Materials (ICMM) and Bavarian Polymer Institute (BPI), Friedrich-Alexander University (FAU) Erlangen-Nürnberg, Erlangen, Germany

**Keywords:** nanostructures, organic-inorganic hybrids, photocatalysis, self-assembly, stimuli-responsiveness, structure analysis, supramolecular chemistry, thermodynamics

## Abstract

The design of functional nano-objects by electrostatic self-assembly in solution signifies an emerging field with great potential. More specifically, the targeted combination of electrostatic interaction with other effects and interactions, such as the positioning of charges on stiff building blocks, the use of additional amphiphilic, π−π stacking building blocks, or polyelectrolytes with certain architectures, have recently promulgated electrostatic self-assembly to a principle for versatile defined structure formation. A large variety of architectures from spheres over rods and hollow spheres to networks in the size range of a few tenths to a few hundred nanometers can be formed. This review discusses the state-of-the-art of different approaches of nano-object formation by electrostatic self-assembly against the backdrop of corresponding solid materials and assemblies formed by other non-covalent interactions. In this regard, particularly promising is the facile formation of triggerable structures, i.e. size and shape switching through light, as well as the use of electrostatically assembled nano-objects for improved photocatalysis and the possible solar energy conversion in the future. Lately, this new field is eliciting an increasing amount of understanding; insights and limitations thereof are addressed in this article. Special emphasis is placed on the interconnection of molecular building block structures and the resulting nanoscale architecture via the key of thermodynamics.

## 1 Introduction

The creation of all architectures and structures found in Mother Nature is credited to self-assembly. On the lowest and essential level of evolution, DNA base pairs assemble themselves and enable life. Self-assembly further generates many complex architectures and functions in nature, such as the structure of the tobacco virus, chloroplasts in leaves, the structures in the retina that enables us to see, the inorganic-organic structures in bones, and many more. This kindles a strong desire for synthetic, self-assembled materials and structures. Self-assembled — or supramolecular — structures are formed without the formation of covalent bonds, and self-assembly can be described as “chemistry beyond the covalent bond” (Lehn, 1988). The various applications of such assemblies include optical devices, sensors, drug delivery, solar energy conversion, switches, and catalysts. The research on self-assembly has so far yielded two Nobel prizes in chemistry. In 1987, Jean-Marie Lehn was awarded together with Donald J. Cram and Charles J. Pedersen, for his work on supramolecular chemistry. He investigated molecular recognition and host-guest interactions on cryptands (Lehn, 1995). In 2016, Fraser Stoddart, Jean-Pierre Sauvage and Ben Feringa received the Nobel prize for designing and synthesizing molecular machines, which are based on self-assembly using ring structures (Balzani et al., 2000). Considering the role of energy input and dissipation, George M. Whitesides distinguished between static and dynamic self-assembly (Whitesides and Grzybowski, 2002). However, this strict categorization has its limits, especially for many more complex systems currently being investigated.

The structure formation without a covalent bond is possible by several effects and interactions — the hydrophobic effect, hydrogen bonds, π-π interactions, and electrostatic interactions. Unlike classical routes, for example, micelle formation, the creation of stable nano-objects in solution by electrostatic self-assembly has only been established in recent years. Currently, a great potential for the design of functional and switchable structures by electrostatic self-assembly has become evident. Here, the combination of electrostatic interaction with other secondary interaction effects is often exploited, promoting the non-directional spherical symmetric electrostatic potential to a directional structure-designing effect. The great capability of the approach lies in its versatility, as the availability of a large variety of ionic building blocks can allow for an effective targeted structure and function design. In this review article, we will discuss recent developments in electrostatic self-assembly in solution, in front of the background of, and in conjunction with, the self-assembly by other forces. After an introductory part regarding self-assembly by different interactions (Section 2), we will focus on electrostatically driven structures. First we will address the essence of the more established layered and material-based systems (Sections 3, 4). Then we will present in detail and comprehensively the formation of nano-objects by electrostatic self-assembly in solution (Sections 5–8). We will demonstrate the impressive structural variety and discuss the state of knowledge regarding structure-directing principles. Concerning functionality, we will provide an overview of switchable structures that respond to various triggers — in particular pH and light — and photocatalytically active structures created by electrostatic self-assembly (Sections 10, 11). While an immense future potential of versatile and functional structures becomes evident, it is also very apparent that a fundamental understanding of the structure formation principles is most crucial for exploiting the capability of this type of electrostatic self-assembly.

## 2 Self-Assembly by Different Interaction Forces

In the hydrophobic effect, amphiphilic components like surfactants possessing a hydrophilic and hydrophobic part classically form micelles in water (Tadros, 2005). The free energy of the system decreases as the entropy of the water molecules increases when they no longer have to order around hydrophobic tails upon micelle formation (Southall et al., 2002; Chandler, 2005). The importance of the hydrophobic effect in protein folding and other biological materials was recognized early (Tanford, 1962; Tanford, 1978), and the formation of supramolecular materials has been extended from surfactants to amphiphilic block copolymers (Antonietti et al., 1996; Li et al., 2004). In addition to vesicles (Kukula et al., 2002), complex structures such as trefoil knots can be created (Cougnon et al., 2018). Furthermore, the hydrophobic effect plays an important role in reactions involving ligand-metal catalysts like the Suzuki-coupling where the ligands generate hydrophobic oxygen-free cores for the metal (Lipshutz et al., 2018).

Hydrogen bonds are defined as interactions between a positively polarized hydrogen atom and a highly electronegative atom, such as an oxygen or nitrogen atom where the hydrogen atom acts as the electron acceptor and the nitrogen or oxygen atom is the electron donor (Arunan et al., 2011). The strength of hydrogen bonds varies between 5 kJ mol^−1^ up to >100 kJ mol^−1^ (Emsley, 1980; Grabowski, 2004). Besides the interaction of water molecules (Latimer and Rodebush, 1920), hydrogen bonds are responsible for the folding of proteins, for DNA base pair association (Watson and Crick, 1953), causing viruses such as SARS-CoV-2 to bind to the receptors (Pauling et al., 1951; Wang et al., 2020). The formation of these bonds can be verified by several analytical methods like IR spectroscopy and NMR measurements (Bakels et al., 2019). In ^1^H-NMR spectroscopy, the hydrogen-bonded proton shows a downfield shift as decreasing electron density has a de-shielding effect (Steiner, 2002). A different method to detect hydrogen bonds is by atomic force microscopy (AFM) where a modified tip recognizes the interaction between the molecules (Boland and Ratner, 1995). By using a special operation mode, dynamic force microscopy, hydrogen bonds can be made visible (Sweetman et al., 2014). Yet this is still controversially discussed as the probe molecule used to modify the AFM tip can cause fake images of intermolecular bonds (Hämäläinen et al., 2014).

In contrast to van-der Waals-forces, hydrogen bonding is a directional force. One of the first non-biological systems using hydrogen bonds for self-assembly linked two chromophores with alternated side hydrogen bonding complementary groups (Tecilla et al., 1990). The directing properties of hydrogen bonds are often used for self-assembly to create dimers or polymeric structures (Brunsveld et al., 2001; Schmuck and Wienand, 2001). For the formation of self-assembled structures, hydrogen bonds often are combined with π-π stacking, electrostatic interactions, or halogen bonding (Schmuck and Wienand, 2003; Drain et al., 2005). Meijer and coworkers introduced self-assembled helical structures of bifunctional triazines linked by a polymer (Hirschberg et al., 2000; Brunsveld et al., 2002; Terashima et al., 2011). The backbone of the helical polymer is formed by π-π stacking. For example, bipyridine-based discs and functionalized oligo (*p*-phenylene vinylene)s, which both have a C3-symmetry can serve as the backbone for supramolecular fibers (van Gorp et al., 2002; George et al., 2004). Another backbone for helical supramolecular polymers are perylene diimides, polycyclic aromatic hydrocarbons that are often used for π-π interactions (Li M. et al., 2020). Even more complex structures than helices like helix-turn-helix structures composed of helices of different orientations and handedness can be formed via hydrogen bonds (Mazzier et al., 2020). By implementing a hierarchical single, double, and quadruple hydrogen-bonding moieties in a polymer backbone mimicking the folding of proteins, polymers with high tensile strength and self-healing properties are synthesized (Cao et al., 2017; Song et al., 2018; Tamate et al., 2018).

π-π interactions are based on electron-rich systems, which are predominantly aromatic. The strength of the interaction, which can be up to 50 kJ mol^−1^, depends on substituents attached to the π-system (Steed et al., 2007; Mariani et al., 2016a). Geometrically, it is possible to make an edge-to-face, a face-to-face, and a side-by-side staircase-like configuration. For example, porphyrins interact by π-π stacking due to their extended π-systems. Depending on the substituents, the concentration, and further added molecules, porphyrins can form stacks, which can be divided into forming face-to-face H-aggregates, side-by-side J-aggregates, and couple electronically (Pasternack et al., 1972; Maiti et al., 1998; Li et al., 2017). Inspired by the light collecting antenna system in leaves, other dyes like Acid red 26 (Ar26) also form stacks (Neumann et al., 2000). Moreover, π-π stacking offers advantages for supramolecular electronics. Nanowires from self-assembled poly (thiophenes) show high conductivities (40 S/cm) (Bjørnholm et al., 1999). Oligo (thiophene) and copolymers of fluorene or indenofluorene with oligo (thiophene) build fiber structures (Surin et al., 2005; Miller et al., 2018).

Efficient charge transport can also be achieved with small molecules like the planar perylene-bis(dicarboximide) derivatives, which form millimeter-long fibers with a constant cross-section of a few hundred nanometers by solvent-vapor annealing (De Luca et al., 2007), or with porphyrin derivatives, specifically semisynthetic zinc chlorin dyes (Patwardhan et al., 2012). Depending on the exact molecular structure, the zinc chlorin dyes, form either two-dimensional (2D) side-by-side stacks or one-dimensional (1D) tubular assemblies. Diimide-zinc chlorin triads assemble into rod antennae structures of J-aggregated zinc chlorin dyes powered by peripheral light-harvesting chromophores and harvest solar light up to 63% when compared with the natural light-harvesting capacity of bacteriochlorophyll (Röger et al., 2008). Graphene represents an extended π-system where the layers are held together by π-π stacking. Therefore, non-covalent π-π interactions can be used to separate the layers of graphene by perylene bisimides-based bolaamphiphiles which delaminate and stabilize graphene flakes (Englert et al., 2009), and it was shown that the conductivity of carbon nanotubes can be enhanced via π-π mediated self-assembly (Zelada-Guillén et al., 2018). Furthermore, π-π interactions play an important role for aromatic-rich dipeptides, which form organogels and show potential for oil spill recovery and wastewater treatment (Chetia et al., 2020), as well as for reduced graphene oxide (rGO) hydrogel interacting with a perylene derivative, which has water purifying properties and removes over 99.5% of gram-negative *Escherichia coli* (*E. coli*) and gram-positive *Staphylococcus aureus* (*S. aureus*) bacteria (Wang L. et al., 2019).

Electrostatic self-assembly is based on the attraction of oppositely charged ions where the interactions can be between cationic and anionic or between zwitterionic molecules. Although the interaction is strong and long-ranged, and ionic species of various kinds are readily available, the formation of defined nanostructures in solution by electrostatic self-assembly has been achieved only much more recently. The reason probably lies in the missing directionality of the electrostatic potential per se, as a single charge exhibits a spherical interaction potential equally in all directions — as opposed to directed binding motives like hydrogen bonding and π-π interaction. However, recent developments prove that electrostatic self-assembly can indeed yield very well-defined nano-objects in solution. The key here is to apply electrostatics in conjunction with other structure-directing effects. The current research focus is on both developing a fundamental understanding of structure control and the fabrication of an increasing number of architectures that fulfill a certain function. While this is quite new for nano-objects in solution, electrostatic self-assembly is more established on a 2D and 3D level. In what follows, we will thus commence from the 2D films and 3D materials, and come to assemblies in solution thereafter.

## 3 2D Polyelectrolyte Multilayers

**FIGURE 1:**
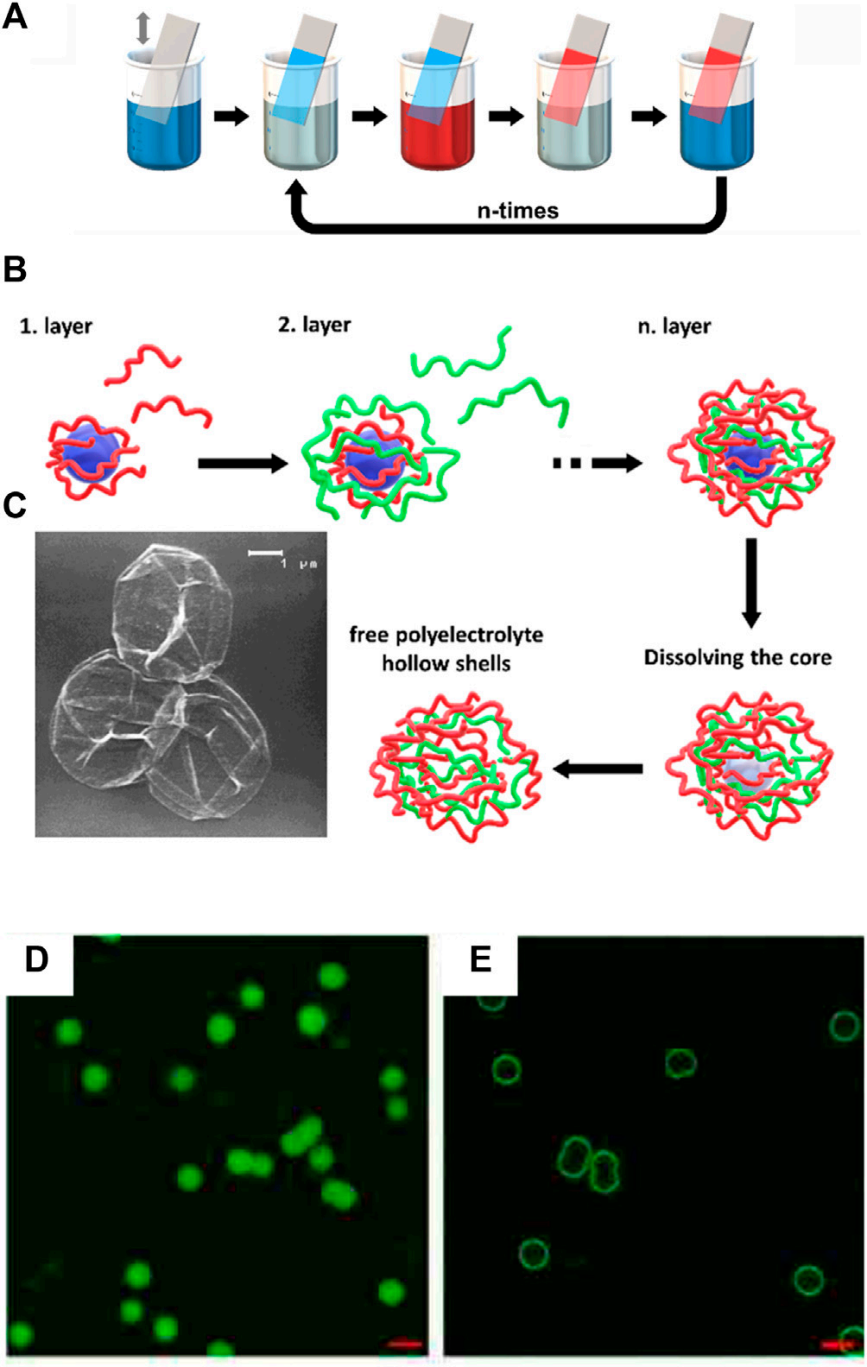
Polyelectrolyte multilayers: **(A)** Scheme of the film deposition process using slides and beakers. Steps 1 and 3 represent the adsorption of a polyanion and polycation, respectively, and steps 2 and 4 are washing steps for the basic buildup sequence (A/B)_n_. **(B)** Capsule formation: Schematic illustration of the polyelectrolyte deposition onto a particle process and of subsequent core decomposition; **(C)** SEM of nine-layer [(poly (styrene sulfonate)/poly (allylamine hydrochloride)_4_/poly (styrene sulfonate)] capsules. Drying, together with the topological constraints of the closed surface, results in a completely folded upper hemisphere (Donath et al., 1998). **(D,E)** Poly (styrene sulfonate)/poly (N, N-dimethylaminoethyl methacrylate) capsules with encapsulation of fluorescein isothiocyanate: **(D)** at pH = 9 and **(E)** release at pH = 7; Scale bar 5 μm (Xu et al., 2014). Reprinted with permission. Copyright ^©^ 1998 WILEY-VCH Verlag GmbH, Weinheim, Fed. Rep. of Germany. Copyright ^©^ 2014 American Chemical Society.

In 1966, Iler et al. were the first to utilize electrostatic interaction for the material design. Oppositely charged colloids of silica and alumina were deposited on a surface alternately. (Iler, 1966). The possibility to extend the layer-by-layer method to surfactants and polymers was identified. Decher et al. established the formation of polymer films by cationic and anionic polymer layers in 1992. This technique was successful for the formation of several charged polymers and the film thickness increased to over 100 layers and several millimeters. (Decher et al., 1992). By alternately dipping a surface in the sodium poly (styrene sulfonate) and poly (allylamine hydrochloride) polymer solutions with a rinsing step in between resulting in films with an ABABAB … -structure as depicted in Figure 1.

The layer-by-layer deposition technique cannot only be used to fabricate 2D structures like films. Möhwald et al. devolved hollow capsules of layered charged polymers. Polymer layers of poly (sodium styrene sulfonate) and poly (allylamine hydrochloride) are absorbed on weakly cross-linked melamine-formaldehyde colloidal particles. The core colloid can be dissolved in the end to form hollow 3D polymer capsules as shown in Figures 1B,C (Decher, 1997; Donath et al., 1998; Sukhorukov et al., 1998a; Sukhorukov et al., 1998b). Layer-by-layer capsules are stimuli-responsive to pH and temperature and can be opened to release with target molecules (Figures 1D,E) (Majumdar et al., 2003; Xu et al., 2014) As an alternative to polymers, other polyelectrolytes like proteins or epigallocatechin gallate can be used to form capsules (Caruso and Möhwald, 1999; Shutava et al., 2009). The broad variety of suitable building blocks allows potential applications of this method for drug delivery (Shutava et al., 2009; Matsusaki et al., 2012), or filters for wastewater treatment (Guzmán et al., 2020).

Organic-inorganic hybrid material films like gold or zirconium dioxide particles implemented in polymer films can be manufactured by an electrostatic layer-by-layer approach also (Liu et al., 1998; Rosidian et al., 1998). The application of these materials ranges from sensors to catalysts and nanoelectronic devices (Arregui et al., 1999; Chirea et al., 2007; Tian et al., 2019). Films of gold colloids protected by poly (diallyl dimethyl ammonium chloride) alternated with poly (styrene sulfonate) layers can be used as humidity sensors since the environmental conditions can lead to a swelling of such layer-by-layer films (Arregui et al., 1999; Wong et al., 2004). For-layer-by-layer films, it is not just the chemical nature of the polyelectrolytes, their counterions, or the polymer length that influence film thickness; the adsorption conditions also play a role. Hence higher temperatures lead to thicker films due to an increase in the polymer-solvent interaction (Büscher et al., 2002). Sukhishvili et al. showed that even zwitterionic polymers can be exploited for electrostatic layer-by-layer assembly (Kharlampieva et al., 2007).

## 4 Spontaneous Electrostatic Self-Assembly

The electrostatic interaction between cationic and anionic building blocks can also be exploited directly in solution. Here, building blocks are mixed in one pot rather than in a step-by-step procedure. This allows a fast and facile fabrication of new materials. It enables the use of a wide variety of building blocks in a tool-box principle: Molecules like polyelectrolytes, surfactants, dyes, or other charged small particles can serve as building blocks in various combinations and lead to a variety of structures and properties.

### 4.1 Polyelectrolyte-Polyelectrolyte Aggregates

The aggregation of two oppositely charged polyelectrolytes has been studied extensively since quite early. (Kurokawa et al., 1980). When oppositely charged polymers interact with each other, so-called polyelectrolyte complexes are formed. Due to potential biomedical applications, complexes of polycations with chitosan and cellulose, and poly(L-lysin)-poly(saccharide) complexes were investigated (Sato and Nakajima, 1975; Kawashima et al., 1985; Chavasit et al., 1988; Argelles-Monal et al., 1990; Xu et al., 2010). The primary complex formation is due to the electrostatic interactions and takes place rapidly. It was postulated that in a second step, the polymers rearrange within the complex, and in a third step, the polyelectrolyte complexes interact with each other and precipitate (Tsuchida, 1994). The stoichiometry, composition and structure of the polyelectrolyte complexes depend on the polymer chemistry, concentration, molecular mass, pH levels, salt concentration, mixing ratio, hydrophobicity and ionic strength (Tsuchida, 1994; Dautzenberg et al., 1996; Biesheuvel and Cohen Stuart, 2004; Starchenko et al., 2012). Depending on the molar mass and charge ratio, so-called “ladder-like” or “scrambled egg-like” aggregates have been reported (Starchenko et al., 2012; Weber et al., 2018). It has also been discussed that for smaller molar masses, i.e. less highly charged components, more uniform structures can be formed, while for higher molar masses kinetic effects usually lead to a broader size distribution. Overall, these polyelectrolyte assemblies often have a rather undefined form and size and tend to agglomerate and precipitate with time, due to the high charge numbers and kinetic processes involved in the assembly (Chollakup et al., 2010). Müller et al. used cylindrical polyelectrolyte brushes to introduce a larger extent of structural control-in combination with linear polyelectrolytes, leading to structures ranging from worm-like to spheres depending on the composition (Starchenko et al., 2012). Moreover, polyelectrolyte-polyelectrolyte materials can originate from the coating of spherical thermoresponsive microgels with linear polyelectrolytes (Kleinen et al., 2010).

### 4.2 Inorganic Nanoparticle Aggregates

In 2006, Grzybowski et al. presented the self-assembly of oppositely charged, equally sized gold and silver nanoparticles, leading to the formation of large 2-µm-sized diamond-like crystals (Kalsin et al., 2006a). The same authors also showed that mixing oppositely charged inorganic nanoparticles could lead to core-shell aggregates, with an excess of one kind electrostatically stabilizing them in the solution (Kalsin et al., 2006b; Bishop and Grzybowski, 2007; Kalsin et al., 2007; Smoukov et al., 2007). The ligands’ charge on the nanoparticle surface allows control over the nanoparticles’ ion-like behavior, making them suitable as both positively and negatively charged building blocks. As a whole, the super-crystals with solid nanoparticle interiors behave according to DLVO (Derjaguin-Landau-Verwey-Overbeek) theory. These surface-modified charged nanoparticles represent an excellent model system with which to study spherical macroions’ electrostatic behavior, whereas few nanoscale architectures are realizable for solution assemblies. Aggregates precipitate at charge stoichiometry. With regard to the stable non-stoichiometric assemblies, parallels can be seen with the — mostly organic — nano-objects created with structural counterions, to be discussed in the next section. Both approaches evolved at around the same time but were brought forward by different groups. Unlike Grzybowski’s “supercrystals,” most nano-objects built via the structural-counterion concept inherently form assemblies with an excess of one component, even at an experimentally set stoichiometric composition, as will be discussed in detail in the next section.

### 4.3 Polyelectrolyte-Surfactant Assemblies as 3D Material

Polyelectrolyte-surfactant materials formed by the electrostatic interaction of polyelectrolytes with oppositely charged surfactants have the advantage over the polyelectrolyte-polyelectrolyte materials in that they are often much more defined in structure. One reason is the lower charge of one of the components that causes the kinetic effects in the structure formation to be less expressed (Antonietti et al., 1994; Thünemann and Lochhaas, 1998). Secondly, the electrostatic attraction is here combined with the association of surfactants due to the hydrophobic effect. The surfactant molecules that first attach electrostatically to the oppositely charged polyelectrolyte bind in a cooperative process preferably next to each other. The result is a material that is internally structured due to the presence of an ion pair and a hydrophobic subphase each with nanoscale extension. With this combination of non-covalent interactions, materials with a wide variety of architectures have become accessible, with structures in analogy but also beyond the ones of classical surfactants, here solidified into a material.

In 1994, Thüneman and Antonietti introduced this new class of assembled structures for cationic polyferrocenylsilane polyelectrolytes in combination with various anionic surfactants. By combining the surfactant and the polyelectrolyte mesostructures precipitate which are redox-active due to the polyferrocenylsilane. The type of surfactant contributes to the design and liquid-crystalline properties of the material (Antonietti et al., 1994). Highly transparent and flexible films of lamellar structures are obtained by assemblies of surfactants, containing trimethylsilyl groups, and a cationic polyelectrolyte (Thünemann and Lochhaas, 1998). Poly (ethyleneimine) with perfluorinated carboxylic acids as surfactants form liquid-crystalline mesophase structures with “super-hydrophobic surfaces” with tunable wettability (Thünemann, 2000). The implementation of double-tail dialkyl phosphate surfactants layers in conjugated oligoanilines by spontaneous electrostatic self-assembly (without a layer-by-layer technique) allowed a temperature-controlled switchability of the films due to disorder (Wei et al., 2005). Meanwhile a wide variety of such materials exists, including the use of supramolecular metallo-polyelectrolytes with surfactants (Kurth et al., 2000), or small ionic dendrimers with surfactants (Hernández-Ainsa et al., 2011; Castillo-Vallés et al., 2020).

### 4.4 Surfactant-Dye Assemblies as 3D Material

By going to an even lower number of charges of the building block molecules, i.e. using smaller charged molecules for electrostatic self-assembly, Faul and Antonietti presented surfactant-dye materials obtained by what they called “ionic self-assembly” in 2002 (Faul and Antonietti, 2002). In the first study, the surfactant dodecyltrimethylammonium chloride was combined with several azo dyes such as Ar26, Acid red 27 (Ar27), Acid red 18 (Ar18), and Orange C. The results are defined needle-like structures with one-to-one stoichiometry (Faul and Antonietti, 2002). This concept was extended to further anionic azo dyes and the influence of the surfactant tail lengths of trimethylammonium bromides with ten to fourteen carbon atoms in the tails, and the influence of double-tailed dimethylammonium bromides surfactant was analyzed. The highly organized supramolecular tightly packed herringbone architecture with 1 nm thick crystalline planes is crystalline/liquid crystalline. The dye molecule as well as the volume fraction of the surfactant tails govern the phase structure and the onset of thermal phase transitions (Guan et al., 2002). The liquid crystalline phases within the material are responsible for their anisotropic optical properties upon the irradiation with polarized light. Hence, the surfactant dye materials are alternatives to photosensitive polymers (Guan et al., 2002; Zakrevskyy et al., 2006). The optical anisotropy depends on phase structure and consequently on temperature (Zakrevskyy et al., 2006; Faul, 2014).

Apart from azo dye-based surfactant-dye materials exist surfactant perylenediimide complexes formed from the surfactant (bis(2-ethylhexyl) sulfosuccinate and cationic N, N′- bis(2-(trimethylammonium)ethylene)-perylene-3,4,9,10- tetracarboxylic diimide bromide as dyes, which have a thermotropic liquid crystalline phase within the perylenediimide part of the structure and a lyotropic liquid crystalline mesophase within the surfactant part of the material (Guan et al., 2003; Zakrevskyy et al., 2004). By exchanging the surfactant with a chiral lysine-based surfactant, the chiral information of the surfactant is transferred into the supramolecular structure and left-handed helical stacking occurs (Franke et al., 2006).

### 4.5 Dye-Dye 3D Assembled Materials

**FIGURE 2:**
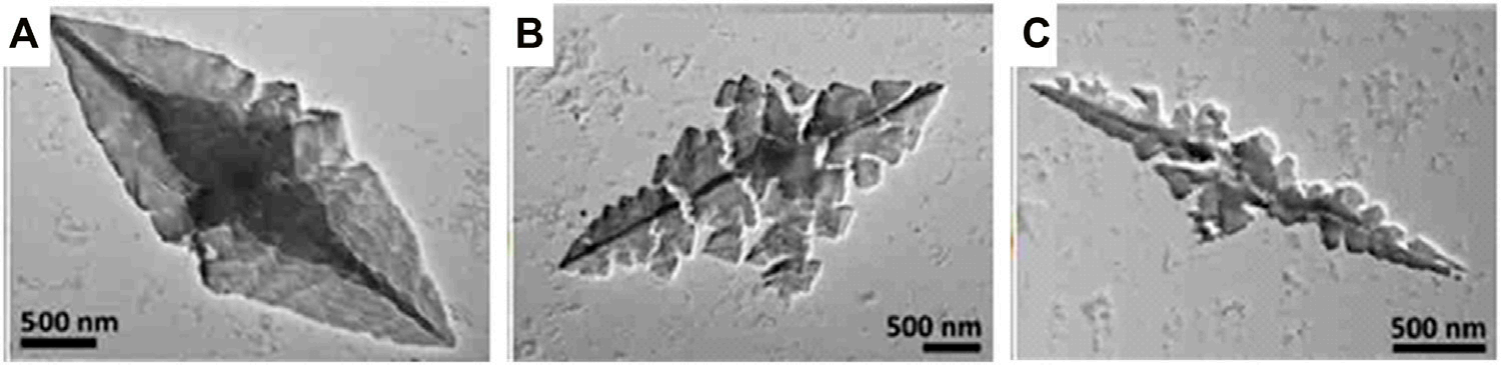
Cationic-anionic di-porphyrin needles: (**A–C**) TEM images of needles formed by an assembly of anionic tetra-(4-sulfonatophenyl)porphyrin and cationic tetra-(N-methyl-4-pyridyl)porphyrin with different ratios (Düring et al., 2018). Reprinted with permission. Copyright ^©^ 2018, Springer-Verlag GmbH Germany, part of Springer Nature.

Ionic dye-dye aggregation is a rather new route to electrostatically self-assembled materials. Aggregation leads to large complex-shaped entities that usually precipitate. For example, perylenediimide and chiral porphyrins form long helical fiber-like structures (Guan et al., 2005). The combination of oppositely charged Zn- and Sn-porphyrins lead to various flower-like structures depending on the temperature and composition (Martin et al., 2010; Martin et al., 2013) and oppositely charged Fe^3+^-porphyrins form bulky flower-like structures (Xie et al., 2019). Anionic tetra-(4-sulfonatophenyl)porphyrin and cationic tetra-(N-methyl-4-pyridyl)porphyrin form ionic organic solid microneedles with various ratios as depicted in Figure 2, which are photocatalytic active (Düring et al., 2018).

Assemblies of tetrakis (4-carboxyphenyl)porphyrin and tetrakis (1-methylpyridinium-4-yl)porphyrin *p*-toluenesulfonate are organic crystalline nanofibers that show an aggregation-induced chromism (Rodríguez-Abreu et al., 2020). In all these structures, it is again taken advantage of combining two different non-covalent interactions, in this case, electrostatics and π-π interactions.

### 4.6 Polyelectrolyte Assemblies With Block-Polyelectrolytes

**FIGURE 3:**
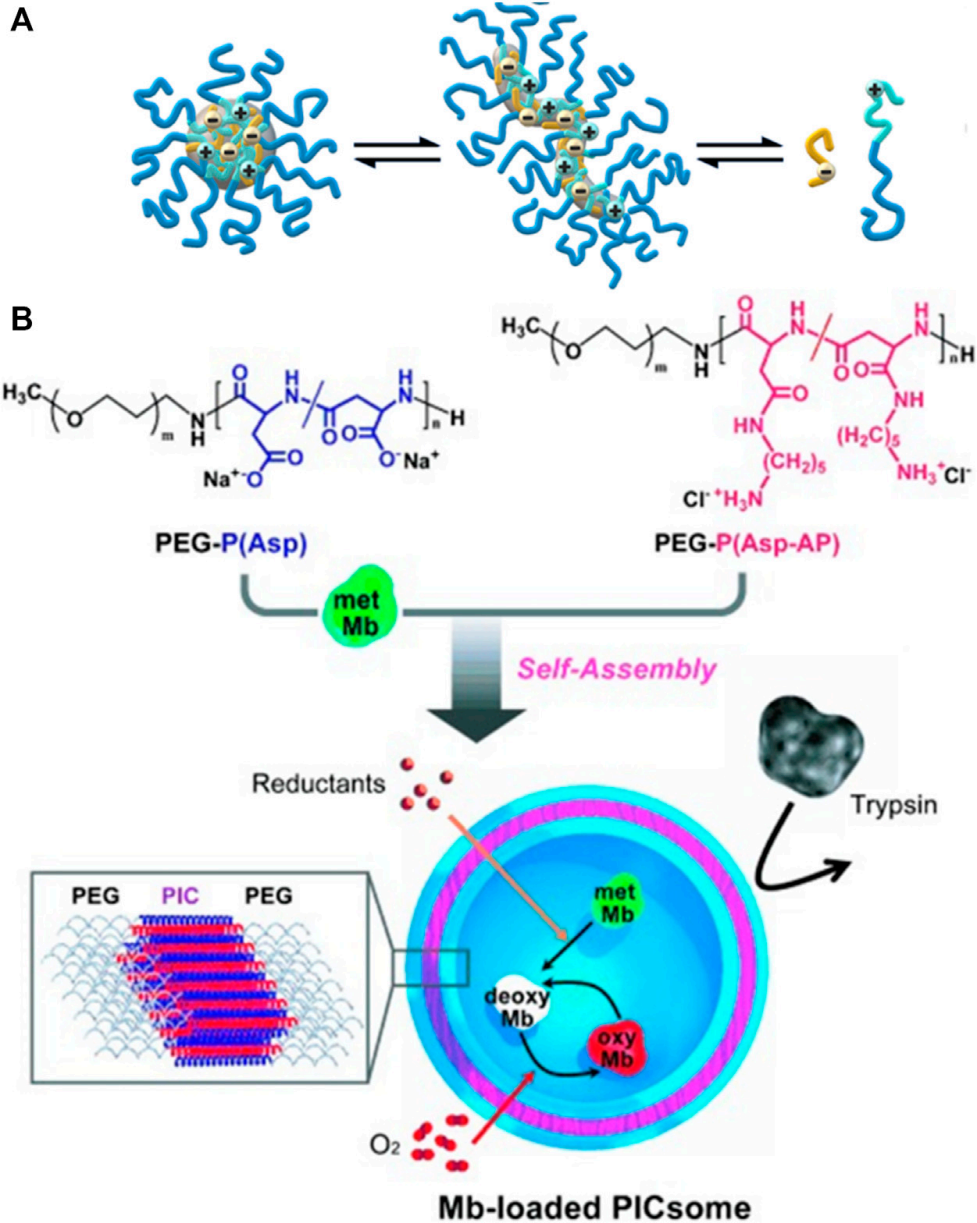
Electrostatic self-assembly with double-hydrophilic block-polyelectrolytes: **(A)** Formation of spherical complex coacervate core micelles from anionic poly (acrylic acid) (red) and block copolymer consisting of a cationic poly (N-methyl-2-vinylpyridinium) (blue) and a neutral poly (ethylene oxide) block (green); An increase in the salt concentration leads to the formation of an elongated worm-like structure; an even further increase in the salt concentration above the critical salt concentration leads to the separation of the polymers (van der Kooij et al., 2012); **(B)** Reversible myoglobin (Mb) oxygenation inside the polyion complex membrane self-assembled from a pair of oppositely charged block polyelectrolytes (Kishimura et al., 2007). Reprinted with permission. Copyright ^©^ 2007 WILEY-VCH Verlag GmbH and Co. KGaA, Weinheim.

As a special case of polyelectrolyte-polyelectrolyte materials, also diblock copolymers and triblock copolymers with polyelectrolyte blocks have been used for electrostatic self-assembly. Here, oppositely charged polyelectrolyte blocks attach to each other, forming the insoluble interior of a micelle, while an uncharged hydrophilic block forms the corona of the micelle-like structure, which allows for the formation of stable and well-defined micelle-like complexes in solution (Chollakup et al., 2010; Tsitsilianis et al., 2000; Voets et al., 2006a; Voets et al., 2006b; Voets et al., 2009; van der Kooij et al., 2012; Yan et al., 2008a; Yan et al., 2008b; Wang et al., 2010; Wang J. et al., 2019; Zhou et al., 2019; Procházka et al., 1996; Srivastava et al., 2020; Marras et al., 2021). Cohen Stuart et al. investigated various systems of these so-called polyelectrolyte complex micelles (PCMs) which form a variety of defined structures. The shapes can range from spherical micelles to more complex disk-like micelles with a coacervate core and an asymmetric corona (Voets et al., 2006a; Voets et al., 2006b; Voets et al., 2009). For complex coacervate core micelles formed from poly (acrylic acid) and poly (N-methyl-2-vinylpyridinium)-b-poly (ethylene oxide), the shape can be changed by the added salt concentration from spherical micelles to elongated structures below the critical salt concentration if the poly (acrylic acid) homopolymer is not too long as depicted in Figure 3A. Other block copolymers form raspberry-like precipitates due to electrostatic interactions (Li et al., 2008). Moreover, it is possible to form semipermeable polymer vesicles from two oppositely charged block copolymers, which can be used as oxygen carriers as shown by Kataoka et al. in Figure 3B (Koide et al., 2006; Kishimura et al., 2007; Anraku et al., 2010; Anraku et al., 2013).

The group of Tirrell also compared block-polyelectrolyte assemblies with zwitterionic corona to the ones with a polyethylene oxide corona (Ting et al., 2020). Assemblies of charged-neutral diblock copolymers and oppositely charged coordination polymers formed from metal ions and bisligand molecules form micelles implementing coordination additional to electrostatic interactions (Yan et al., 2008a; Wang et al., 2010). The metal ions for coordination are, for example, Zn^2+^ or Fe^2+^ ions (Yan et al., 2008a; Yan et al., 2008b; Wang et al., 2010; Wang J. et al., 2019). Lanthanides in the coordination polyanion lead to luminescent hydrogels (Zhou et al., 2019). The assemblies only exist below a critical salt concentration, which is proven by light scattering and cryo-TEM (Yan et al., 2008a). Nanoribbons formed by triblock polymers, containing histidine proteins as biochemical polyelectrolytes, and coordination polymers act as supramolecular polymers. The assemblies are stabilized by a complex interplay of several non-covalent interactions like metal-ligand complexation of Zn^2+^ ions, hydrogen bonding, hydrophobic interactions, and electrostatic interactions (Yan et al., 2008b). Besides the application of polyelectrolyte-polyelectrolyte complexes as carrier systems, electrostatically self-assembled materials of block copolymers show an efficient ion transport and provide mechanical stability making them potential materials for lithium-ion batteries (Sing et al., 2014). Furthermore, electrostatically self-assembled oppositely charged conjugated polymers can function as excitonic donor/acceptor pairs, possessing significant potential as artificial energy transfer antennae as the energy is transferred from the donor to the acceptor in less than 250 fs (Hollingsworth et al., 2018).

Instead of the combination of two block copolymers also a block copolymer and a poly(amidoamine) (PAMAM) dendrimer, which is another type of polyelectrolyte with a highly branched structure, interact electrostatically and forms defined, and stable assemblies in water (Reinhold et al., 2009; Wang et al., 2014; Amaral et al., 2018). Another special case of polyelectrolyte-polyelectrolyte structures yielding interesting structures are charged lanthanide-based coordination polymers together with oppositely charged dendrimers (Huang et al., 2019).

## 5 Nano-Objects by Electrostatic Self-Assembly in Solution

Most of the electrostatic self-assembly discussed above yields materials that precipitate from solution or generates layered structures. Yet for many applications, from drug delivery to catalysis, nanoscale particles in solution are needed and thus, it has also been highly desirable to exploit structure formation principles using electrostatic interactions for forming defined nano-assemblies in solution. For a long time, it was believed that programmable growth limiting factors as naturally present in micelle formation (and in the micelle-analog approach with block-polyelectrolytes) would be generally missing in electrostatic self-assembly. Thus, it is also a fundamental question currently of interest: What are the “exceptions” in the fields of the materials presented above that stay stable in solution, what design concepts are available and how can such assemblies be stable in solution? Answering these questions opens a whole new field of nanostructures in solution, beyond the classical micelle formation, which we will focus ion in the following.

**FIGURE 4:**
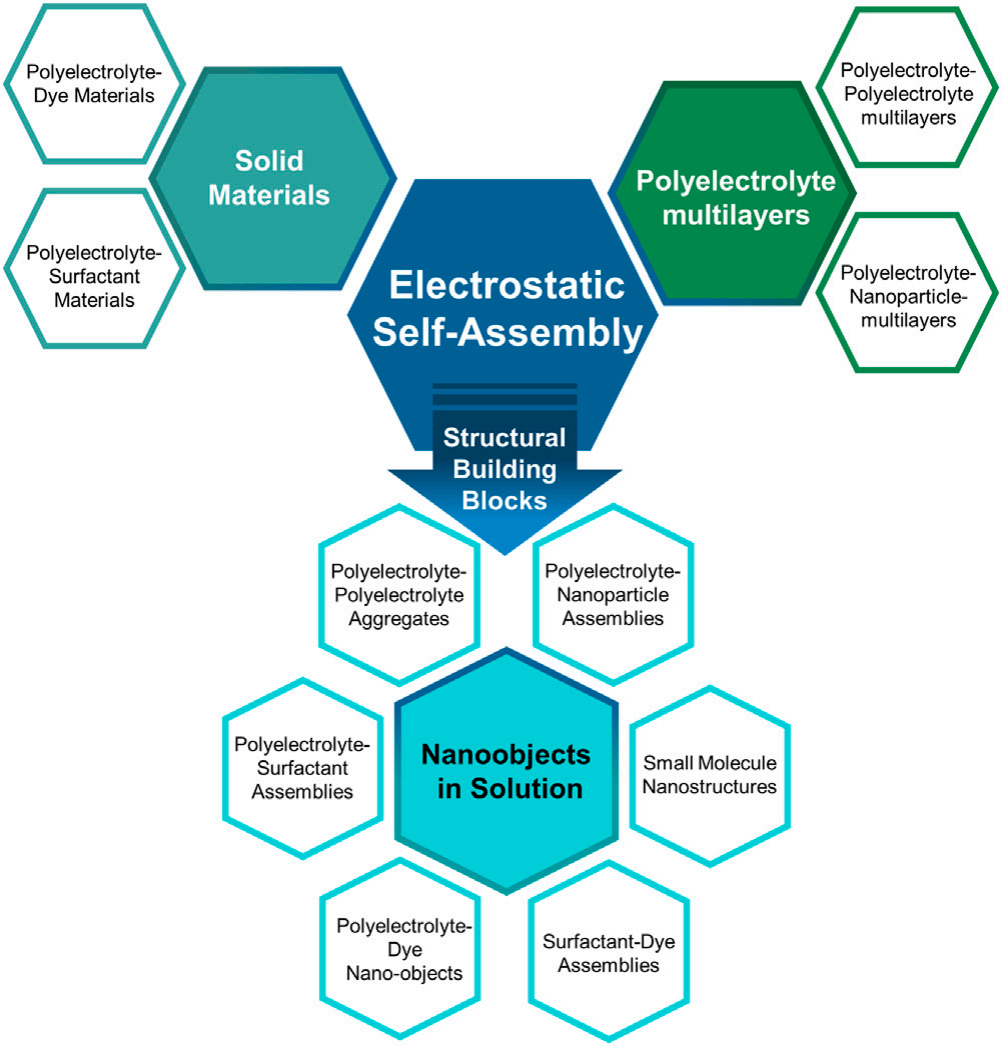
Electrostatic self-assembly: overview of established and emerging strategies.

The Gröhn group introduced a novel concept for forming electrostatically self-assembled nano-objects in solution by using “structural counterions” and presented a variety of supramolecular structures in solution formed by electrostatic self-assembly (Gröhn, 2008; Li et al., 2009; Ruthard et al., 2009; Willerich et al., 2009; Gröhn, 2010; Gröhn et al., 2010; Kutz et al., 2016; Mariani et al., 2017a; Frühbeißer and Gröhn, 2017; Frühbeißer et al., 2018). Similar to the material and the multilayer case, structures from various building block combinations can be created. Figure 4 sketches how the newer approach of forming nano-objects in solution emerges from the more established fields of structure formation by electrostatic interaction, together with typical building blocks for each strategy.

**FIGURE 5:**
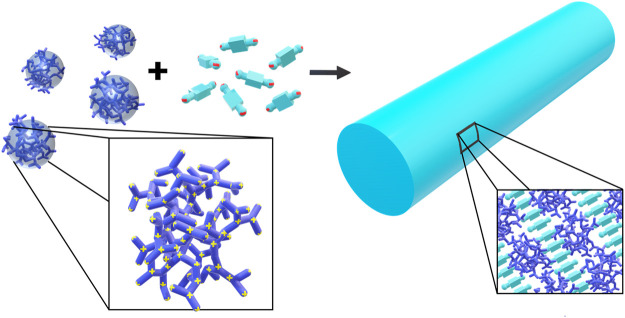
Electrostatic self-assembly in solution: polyelectrolytes interact with oppositely charged stiff, multivalent counterions to form nano-objects with a defined size and shape; for example, cationic 5 nm-sized G4 PAMAM dendrimers interacts with dianionic dye molecules to form a 100 nm scale elongated rod-like stricture with layered internal structure.

The concept is based on self-assembly by electrostatic interactions but utilizes structural counterions which can exhibit secondary effects, such as mutual π-π interactions or geometric factors to direct the structure formation resulting in nanoscale assemblies that are stable in solution with a certain size and shape. In terms of the goal to form defined nanoscale objects, this is in contrast to the nanoparticle supercrystals by Grzybowski (Kalsin et al., 2006a; Kalsin and Grzybowski, 2007) and to the electrostatically self-assembled materials shown above and rather extends the approach of the block polyelectrolyte complexes (in Figure 4 included as polyelectrolyte-polyelectrolyte complexes), albeit toward a more general use of various building blocks apart from block copolymers. The concept of this type of electrostatic self-assembly for the formation of nano-objects in solution is illustrated in Figure 5. Directional effects can be geometric properties like the rigidity or symmetry of the building blocks in solution or secondary non-covalent forces like π-π interaction or hydrogen bonds which enforce the structure control in solution (Koide et al., 2006; Kishimura et al., 2007; Anraku et al., 2010; van der Kooij et al., 2012; Anraku et al., 2013). For example, in a model system, the macroion is a highly branched polyelectrolyte PAMAM dendrimer with distinct geometry, which has several primary and ternary charged groups. The dendrimer interacts with a small organic dye molecule carrying two negative charges. Upon association due to ionic interaction, the dye molecules preferably attach next to each other undergoing mutual π-π interaction. The resulting supramolecular assembly has a defined overall shape and a defined internal structure and is stable in solution. Advantages of well-defined supramolecular structures are the straightforward “synthesis” and handling, which does not have to involve centrifugation or separation of the precipitate and purification processes. As the assemblies are stable in solution, the supramolecular structures are ready to use. Due to the huge variety of possible building blocks the applications of the assemblies range from catalysts to light-harvesting materials for solar energy conversion to switchable structures deployable as sensors (Frühbeißer and Gröhn, 2012; Krieger et al., 2017; Kutz and Gröhn, 2017; Kutz et al., 2018; Mariani et al., 2018).

The size and shape of the structures in solution can be analyzed with light scattering and small-angle neutron scattering (SANS) accompanied by several microscopic techniques, which reveal the assemblies as dried structures on a surface. Spectroscopic techniques such as UV/Vis and fluorescence spectroscopy can give an insight view into the internal structure. In the following, we will first present experimental results on the versatile structures formed and how the nanoscale structure can be tuned via the macroion architecture and size, counterion, building lock ratio, etc. Thereafter, the design principles will be elucidated from a thermodynamic and molecular point of view according to the state of knowledge.

## 6 Structural Variety of Supramolecular Assemblies in Solution

**FIGURE 6:**
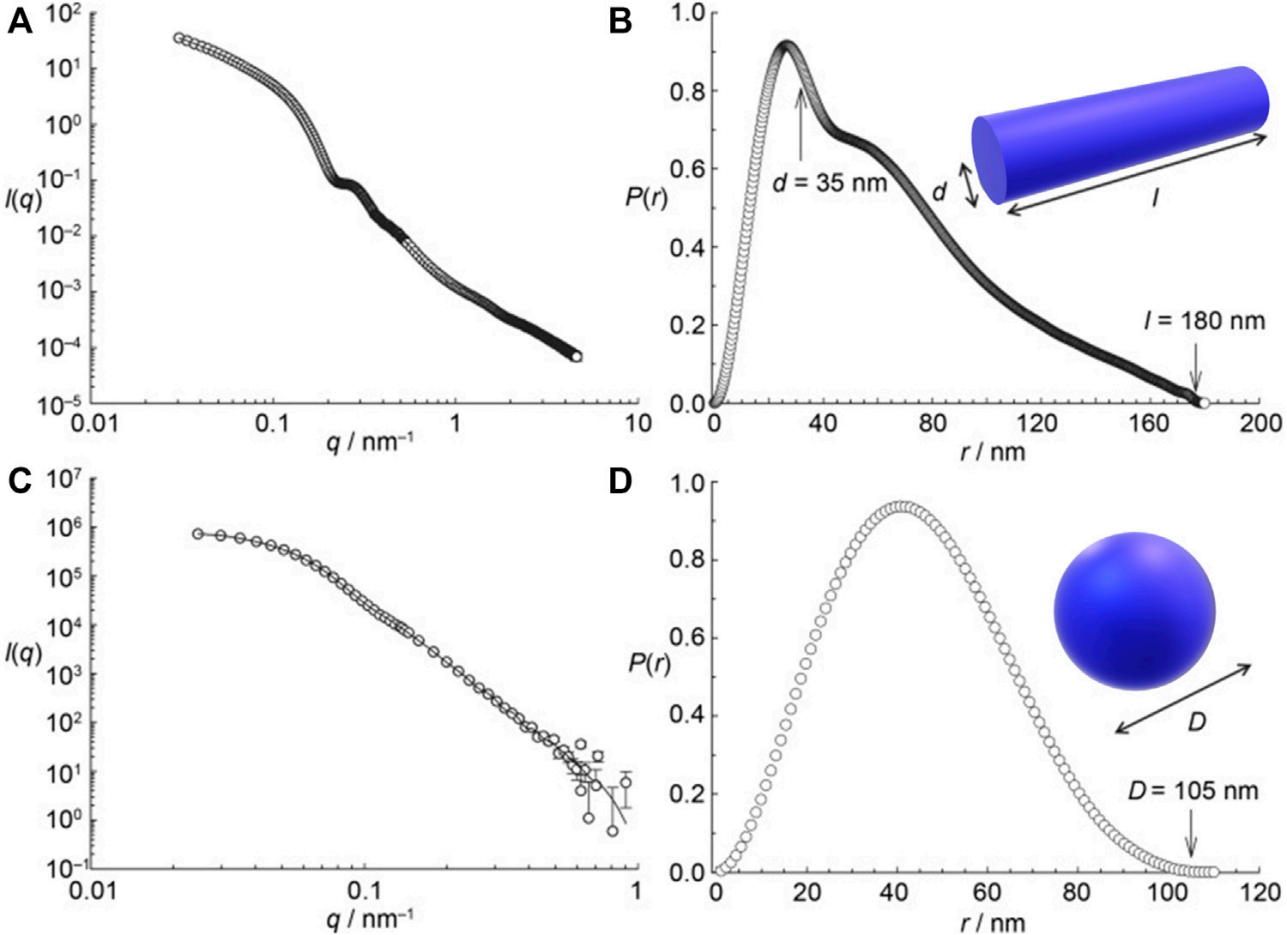
Small-angle scattering characterization of dendrimer-naphthalene dicarboxylic acid dye assemblies: **(A,B)** with 1,4-naphthalene dicarboxylic acid; **(C,D)** with 2,3 -naphthalene dicarboxylic acid; both with a loading ratio of 2:1; left: scattering curves I(q), right: pair distance distribution functions P(r). I(q) and P(r) are in arbitrary units. 1,4-NDC yields cylindrical aggregates but 2,3-NDC spherical aggregates (Gröhn et al., 2008). Reprinted with permission. Copyright ^©^ 2008 WILEY-VCH Verlag GmbH and Co. KGaA, Weinheim.

In the first proof of concept for this type of structure formation, stable assemblies of cationic PAMAM dendrimers of the fifth generation (G5) and 1,4- or 2,3-naphthalene dicarboxylic acids with defined size and shape were formed in methanolic solution. Here, the building blocks interact by electrostatic interaction and π-π interactions of the naphthalene dicarboxylic acid, as confirmed by a shift in the absorption band. Although the naphthalene dicarboxylic acid isomers are quite similar and vary only in the position of the acidic groups the generated nanostructures differ substantially: As the small-angle scattering data depicted in Figure 6 shows, 1,4-naphthalene dicarboxylic acid forms cylindrical aggregates and 2,3-naphthalene dicarboxylic acid spherical aggregates (Gröhn et al., 2008).

**FIGURE 7:**
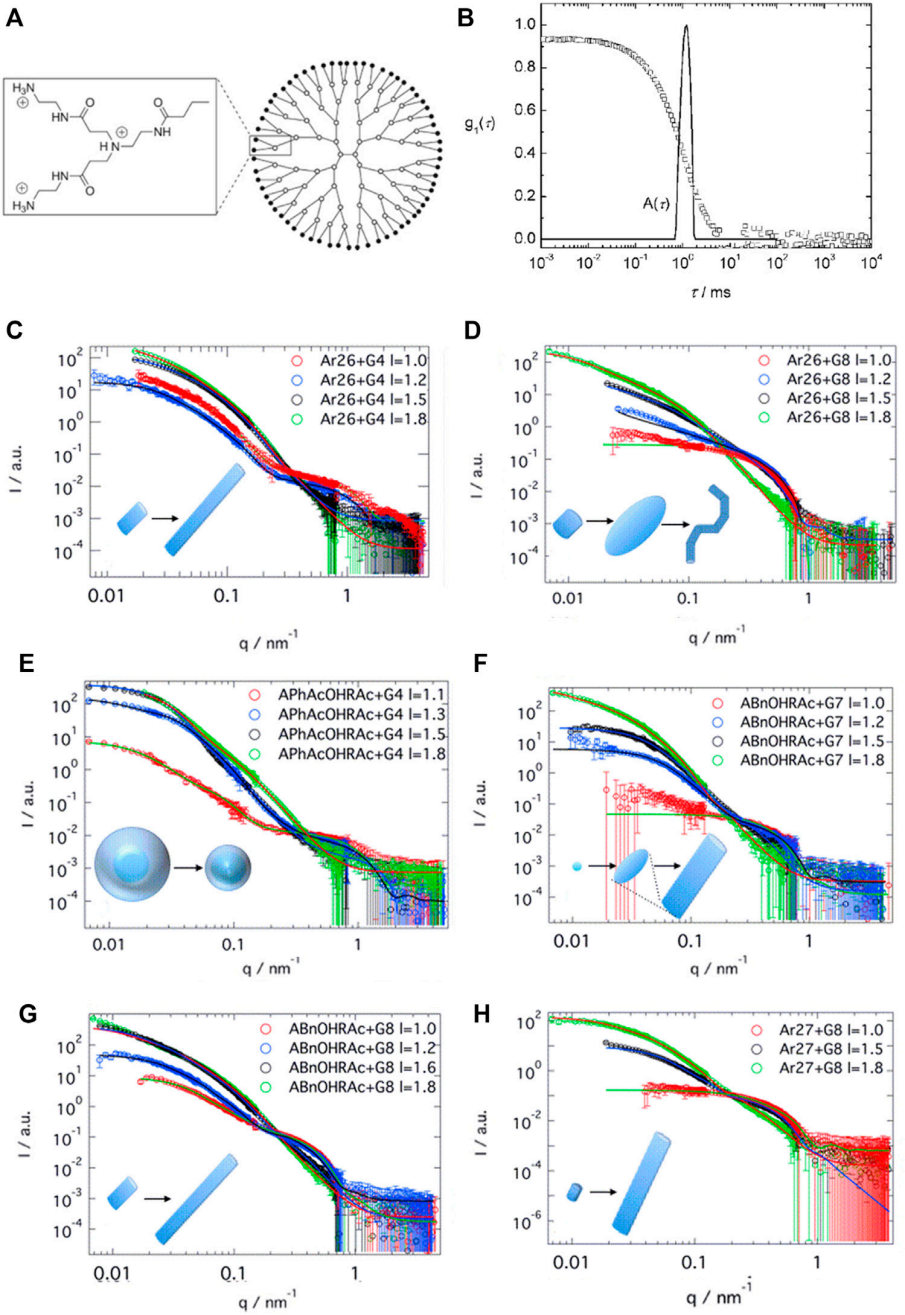
Scattering analysis of a dendrimer dye model system for electrostatic self-assembly in solution: **(A)** Dendrimer structure; **(B)** Dynamic light scattering (DLS): electric field autocorrelation function and decay time distribution at a scattering angle θ = 90° for Ar44-G4 dendrimer with a ratio l = 2.1 (Willerich et al., 2009); **(C–H)** SANS and resulting nanoparticle shapes as a function of dye type, dendrimer generation and loading ratio: SANS results for **(C)** Ar26 + G4, **(D)** Ar26 + G8, **(E)** APhAcOHRAc + G4, **(F)** ABnOHRAc + G7, **(G)** ABnOHRAc + G8, and **(H)** SuACAc + G8, each at varying loading ratio. Continuous lines represent the best structural fit (Mariani et al., 2016a). Reprinted with permission. Copyright ^©^ 2008 and 2015 American Chemical Society.

This is quite remarkable. To derive further insights, generation four PAMAM dendrimer-dye (G4) assemblies were studied systematically with a set of oppositely charged aromatic and aliphatic organic azo dyes with two or three sulfonate groups at various positions as counterions. It turned out that both the molecular structure of the dyes and the molar charge ratio of dye to dendrimer charges play an important role in determining the size and shapes of the assemblies, which can be spherical, ellipsoidal, or rod-like (Willerich et al., 2009; Willerich et al., 2010; Willerich and Gröhn, 2011a; Mariani et al., 2016a; Mariani et al., 2016b). An exemplary size distribution determined by light scattering, and an overview of the shape depending on the dye and the loading ratio for G4 PAMAM dendrimers with various azo dyes, as analyzed by SANS, is given in Figure 7.

Overall, the number of charges and molecular structure of the dye molecule determine the basic shape of the assemblies, while the loading ratio controls the aggregation number and size of the assemblies (Mariani et al., 2016a; Mariani et al., 2016b). The charge ratio can be influenced by the amount of added dye but also by the pH. At pH > 7 only the outer primary amine groups of the dendrimers are protonated, and the resulting particles are spherical, whereas at lower pH the ternary amines are protonated additionally and rod-like structures form. For stable particles, there has to be an excess of dendrimer charges (Mariani et al., 2017b). PAMAM dendrimers with the trivalent dye Ar27 form pH-switchable capsules (see section Switchable Structures) (Gröhn et al., 2010). Even ternary systems with two different dyes are possible enabling hetero π-π stacks as directional secondary forces (Mariani et al., 2017a). Thus, it is not only possible to form finite-size assemblies; a large range of structures is also accessible even within this model system. The underlying structure controlling effects will be discussed in the next section.

Further building block combinations with dendrimers have been exploited — for example, ionic dendrimer-ionic porphyrin combinations (Paulo et al., 2003; Kubát et al., 2005). A direct comparison of dendrimer-porphyrin and linear poly (diallyldimethyl-ammonium chloride) (PDADMAC)-porphyrin assemblies shows the importance of the polyelectrolyte architecture, i.e. geometric effects, for the nanoscale assembly architecture. The less flexible G4 dendrimer leads to spherical particles, whereas the more flexible linear PDADMAC yields rather undefined structures (Frühbeißer and Gröhn, 2017; Frühbeißer et al., 2018). Again different architectures are formed with the flexible ions and with pyrene tetrasulfonate (Yildiz et al., 2009).

So-called coiled-coil structures occur in a designed peptide with porphyrins depending on the pH, which affects the protonation and consequently, the charges of the porphyrins. The peptides show secondary helical folding beside the electrostatic interaction, the π-π interaction between porphyrin-porphyrin and porphyrin-peptide molecules (Kovaric et al., 2006; Kuciauskas and Caputo, 2009).

**FIGURE 8:**
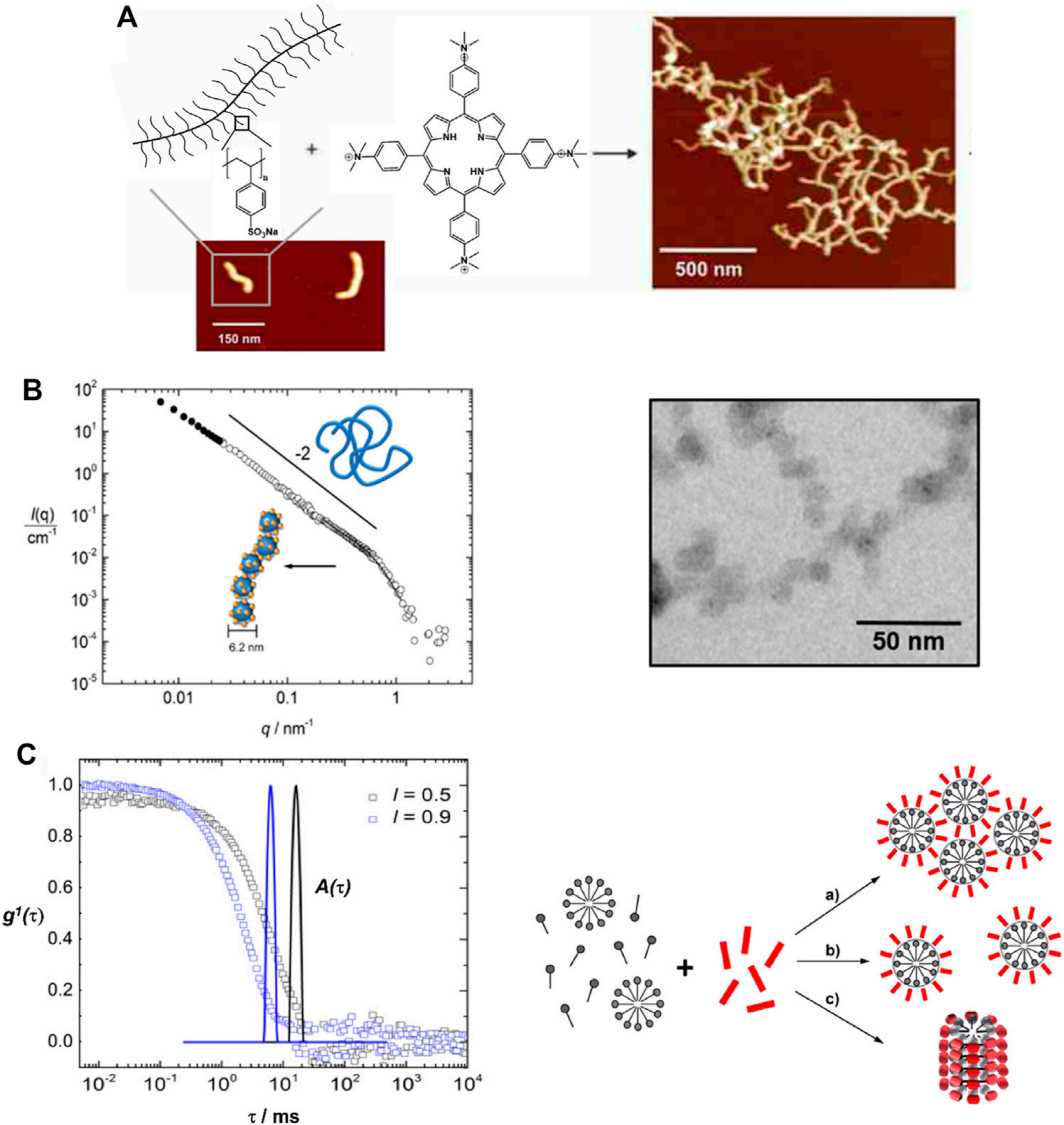
Shape variety in electrostatic self-assembly with different building blocks: **(A)** Wormlike bottle-brush polyelectrolytes and tetravalent porphyrin counterions form finite-size networks; AFM of brush and brush-porphyrin aggregates spin-coated on mica (Ruthard et al., 2009); **(B)** POM–dendrimer assemblies; left: static light scattering and SANS of POM–dendrimer assemblies with l = 0.7; filled symbols: SLS data, open symbols: SANS data, black line at high q: flexible cylinder fit; right: TEM image (Kutz et al., 2018); **(C)** Surfactant micelles connected by Ar26 ions as linkers; left: DLS, electric field autocorrelation function g^1^(τ) and distribution of relaxation times A(τ) for C_12_TAB-Ar26 assemblies; right: overview of structures formed: associated a) and individual spherical surfactant micelles b) with Ar26 molecules acting as connectors and condensed counterions, respectively, and cylindrical surfactant–dye aggregates from cylindrical surfactant micellization with condensed mutually π–π interacting Ar26 counterions c) (Kutz et al., 2016). Reprinted with permission. Copyright ^©^ 2009 American Chemical Society and ^©^ The Royal Society of Chemistry 2018 and ^©^ 2015, Springer-Verlag Berlin Heidelberg.

The tetravalent cationic porphyrin meso-tetrakis(4-N-methyl-pyridinium)porphyrin (TMPyP) or meso-tetrakis(4-(trimethyl-ammonium)phenyl)-porphyrin (TAPP) has also been used as a linker for anionic cylindrical poly (styrene sulfonate) brushes, that is, polymers with a worm-like shape. Electrostatic interactions between the polymer brush and the porphyrin, and π-π interactions in-between the porphyrin molecules create interconnected networks of the polymer brushes with additional stacked porphyrin molecules within the brush, which is depicted in Figure 8A. Salt addition disconnects the brush networks so that single elongated worms of porphyrin-loaded brushes are formed as the electrostatic interactions are weakened (Ruthard et al., 2009; Frühbeißer and Gröhn, 2012). Assemblies with the metalated porphyrins show similar structures, however, for Cu^2+^ the interactions are slightly stronger due to a parallel side-by-side stacking, which leads to H- and J-aggregates (Ruthard et al., 2011a). These polyelectrolyte-porphyrin structures have particular photocatalytic properties, as will be discussed below.

An interesting and functional architecture has been created by phycocyanin combined with a four-armed porphyrin star macroion assembling in a pomegranate-like structure that can serve as a light-harvesting system (Liu Y. et al., 2016). Ternary systems of pillar (Southall et al., 2002) arene, a salicylaldehyde azine derivative and a dye molecule suitable for light-harvesting and stable in solution due to the interaction of several non-covalent forces, were reported to have onion-like spherical structures (Guo et al., 2018).

In a different study, the Keggin-type polyoxotungstate SiW_12_O_4_^4−^ with four negative charges is used as the interconnecting structural counterion with G4 dendrimer, yielding assemblies with expressed photocatalytic activity, as illustrated by the Gröhn group (Kutz et al., 2018). The particles comprise a single dendrimer strain ribbon decorated and interconnected by polyoxometalate (POM) clusters, with a total hydrodynamic radius ranging from 120 nm < R_H_ < 180 nm, with a defined size of these assemblies depending on the loading ratio. SANS reveals assemblies in the shape of a flexible cylinder with a diameter of 6.2 nm, i.e. a chain of dendrimers interconnected by POM clusters (Figure 8B). This is remarkable from a structural point of view, as, unlike the electrostatically self-assembled dendrimer-dye nano-objects, no secondary π-π interaction occurs in this system; only electrostatics in combination with geometric factors and probable dipole effects direct the structure formation here.

Further, surfactants can be combined either with oppositely charged polyelectrolytes or, as micelles, with multivalent “counterions” (Kabanov et al., 1998; Sahoo et al., 2018; Uchman et al., 2012). The above mentioned cylindrical polymer brushes as building blocks have also been used in combination with surfactants in organic solvents by the group of Maskos. (Duschner et al., 2006; Duschner et al., 2008). Ayzner et al. presented polyelectrolyte-surfactant complexes in the solution formed by interconjugated polyelectrolyte complexes which can be separated by ionic surfactants. The assemblies act as light-harvesting antennae and enable the electronic energy transfer above and below the critical micelle concentration (Segura et al., 2019). In some cases, the association of small ionic dendrimers with surfactants can lead to amphiphile assemblies in aqueous solution rather than the macroscopic materials outlined in Section 4.3. Typical amphiphile assemblies such as micelles and vesicles result with common surfactants, while helices were created with an ionic bent amphiphile containing a bent aromatic moiety between head and tail group (Hernández-Ainsa et al., 2011; Castillo-Vallés et al., 2020). In a different system, the Keggin-type polyoxotungstates SiW_12_O_4_^4−^ with four negative charges build onion-like layered spherical architectures with cationic dimethyldioctadecylammonium surfactants, as shown by Sun et al. (Li et al., 2007)

The group of Tianbu Liu explored the interaction of a type of “Keplerate” polyoxometalate macroanionic clusters with cationic surfactants. In difference to the POM building blocks mentioned above, the POM here acts more like a polyelectrolyte, where a large number of surfactants can interact with the macroion. Association with surfactants can lower the macroion charge density. Consequently, the macroions aggregate into larger “blackberries”, which also start to precipitate at a specific amount of surfactant (Kistler et al., 2009).

All these assemblies contain larger macroions; however, oppositely charged small molecules form assemblies in solution by combining several secondary interaction forces as well. Alkyltrimethylammonium surfactants and Ar26 build cylindrical particles of micelles with mutually π-π-stacked dye-counterions, as shown in Figure 8C: The charged surfactant and dye molecules interact by electrostatic interaction, the surfactants form micelles due to the hydrophobic effect and stacking due to π-π interactions between the dyes occurs (Kutz et al., 2016). Thus, this system, on the one hand, represents an analogy to Faul’s dye-surfactant ionic self-assembly for material design and to the polyelectrolyte-dye nano-objects on the other hand. A different approach to self-assembly in aqueous solution using small molecules was introduced by Carsten Schmuck by forming supramolecular structures from small zwitterionic molecules based on the Schmuck binding motif — the guanidiniocarbonyl‐pyrrole zwitterion binding motif (Schmuck and Wienand, 2003; Schmuck et al., 2006; Schmuck et al., 2007; Giese et al., 2020). Here, the zwitterion binding can also be combined with further non-covalent forces like π-π interactions or hydrogen bonding, thus yielding versatile structures with potential from nanostructure design to biomedicine (Schmuck and Wienand, 2003; Rehm et al., 2012).

**FIGURE 9:**
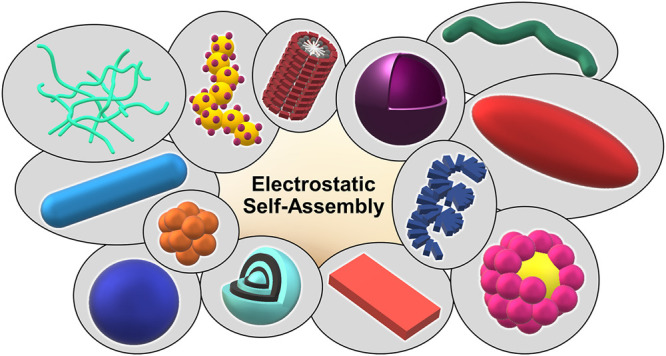
Shape variety achieved by electrostatic self-assembly: networks, decorated wires, stacked micelles, vesicles, tubes, ellipsoids, helices, pomegranate-like structures, discs, onion-like structures, raspberry-like structures, spheres, and rods.

Figure 9 summarizes the enormous shape and structural variety of electrostatically self-assembled structures ranging from spherical particles to elongated wires and rods to raspberry structures of multilayer capsules.

## 7 Understanding the Driving Force in Electrostatic Self-Assembly

Considering the large diversity of possible shapes, the question of what enables the control over the shape, size, and stability in solution of self-assembled aggregates should be addressed. In general, there are two possibilities of reaching the particles. Either the process is kinetically controlled — this means that the self-assembled structures are formed, which have the lowest activation energy but are not the most stable product. In this case, the reaction conditions play a major role, including parameters like temperature and stirring velocity, etc. Or the process is thermodynamically controlled, which means that products with the lowest free energy ΔG are formed, independent of the preparation path. The free energy ΔG is:

ΔG = ΔH – TΔS (1)

where ΔH represents the enthalpy, T denotes the temperature, and S is the entropy.

**FIGURE 10:**
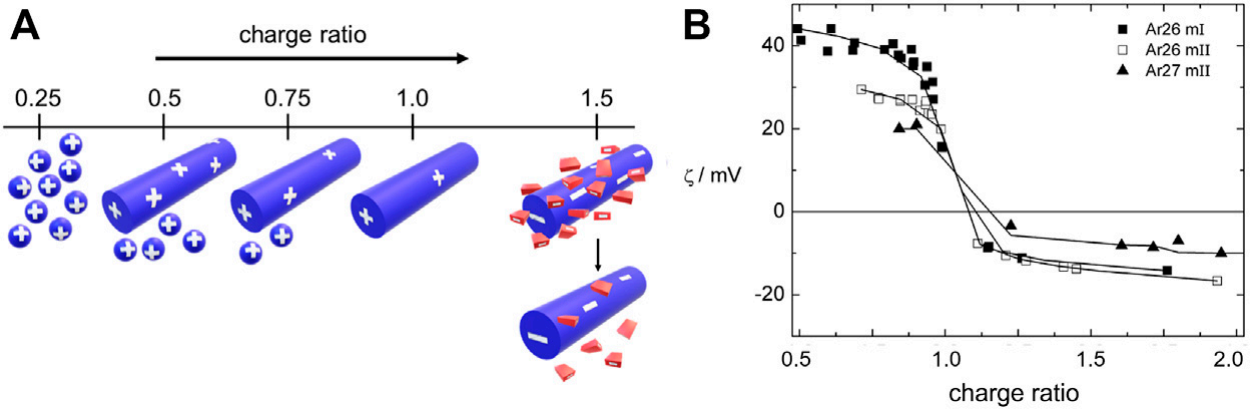
Charge ratio-dependent assembly-formation in a cationic dendrimer-anionic dye system: **(A)** Scheme: small spheres represent individual dendrimers, cylinders larger assemblies, and red rectangles dye molecules. Bound dye molecules are not sketched for simplicity; “+” and “−” represent the sign and relative magnitude of the net charge; **(B)** ζ-potential in dependence on charge ratio (Willerich et al., 2010). Reprinted with permission. Copyright ^©^ 2010 American Chemical Society.

We will mostly focus on thermodynamically controlled structures in this section. The polyelectrolyte dendrimer-azo dye system serves as a model system with the great advantage that features of both building blocks can be systematically varied, the molecular dye geometry and the dendrimer generation. Together with detailed structural and thermodynamic considerations, this has helped derive significant insights. As indicated in Figure 10, the charge ratio of the cationic groups to anionic groups influences the stability and of the assemblies. The charge status of the nano-objects can be analyzed by ζ-potential measurements, given in Figure 10B for the example of a G4 dendrimer with the divalent sulfonate-group carrying azo dyes Ar26 and Ar27.

Here, the loading ratio also determines the presence of co-existing free molecules additional to the dendrimer-dye assemblies (Willerich et al., 2010). The dendrimer-dye assemblies with dendrimer in excess (left on the axis in Figure 10A) show a positive ζ-potential which is lower than for pure dendrimers. The charge density (as derived from the ζ-potential) is reduced due to the association of oppositely charged dye molecules. An excess of dye results in negatively charged assemblies, which are again stable in solution. A similar observation is made for assemblies with other dyes like Acid Yellow 38 (AR38) and Direct Yellow 12 in excess (Willerich and Gröhn, 2011b). Interestingly, at a charge ratio *l* = 1.0, the assemblies themselves have a positive surface charge due to excess cationic dendrimer charges in the actual assembly, as the ζ-potential shows ((In some publications on these systems a pH independent loading ratio *l* was defined, which for pH = 3.5 amounts to twice the charge ratio, with charge stoichiometry lying at *l* = 2.0 at this pH). In accordance, the assemblies’ slightly understoichiometric nature is also evident from isothermal titration calorimetry (ITC; *vide infra*). Thus, at a stoichiometric mixing ratio, in equilibrium, not all dendrimer charges become neutralized; some excess charge remains, which stabilizes the assemblies in solution. This also applies to other systems with other polyelectrolytes like polymer brushes or DNA (Li et al., 2009; Ruthard et al., 2009). By the slight excess of polyelectrolyte charge, the stability in solution is ensured; that is, the dendrimer-dye assemblies are stabilized like an electrostatically stabilized colloid. However, they do not have a compact hydrophobic interior but represent internally swollen hydrophilic species (as was shown by SANS, light scattering, and AFM in conjunction). Individual dendrimers loaded with dye molecules are found only at more extreme excess of dendrimers (loading of just 25% of the dendrimer charges in case of Ar26, Ar27). The most stable assemblies are formed when samples are prepared at a slight excess of dendrimers (loading ratio 0.7–0.95). (Li et al., 2009; Ruthard et al., 2009).

Secondary dye-dye π-π interactions play an important role besides electrostatic forces, as evidenced in the possibility to build stable nano-objects with excess dye. UV/Vis spectra reveal interactions between the dyes due to stacking observed as hypochromic or bathochromic shifts. From loading ratio-dependent UV/Vis spectroscopy on various systems, it is evident that the binding process is cooperative, that is, ionic dye molecules first bind electrostatically to the polyelectrolyte, after which further dye molecules preferably bind adjacently such that they can undergo both electrostatic and π-π interaction (Ruthard et al., 2011a; Willerich and Gröhn, 2011a; Frühbeißer et al., 2016). Here, the π-π interaction is induced as “secondary” interaction via the electrostatic interaction at dye concentrations where these molecules otherwise do not stack (and for dye molecules that without polyelectrolyte do not stack at all). Simultaneously, the multivalent dye molecules interconnect the dendrimers into larger assemblies, rather than forming host-guest complexes as sometimes found for mono- and uncharged molecules.

**FIGURE 11:**
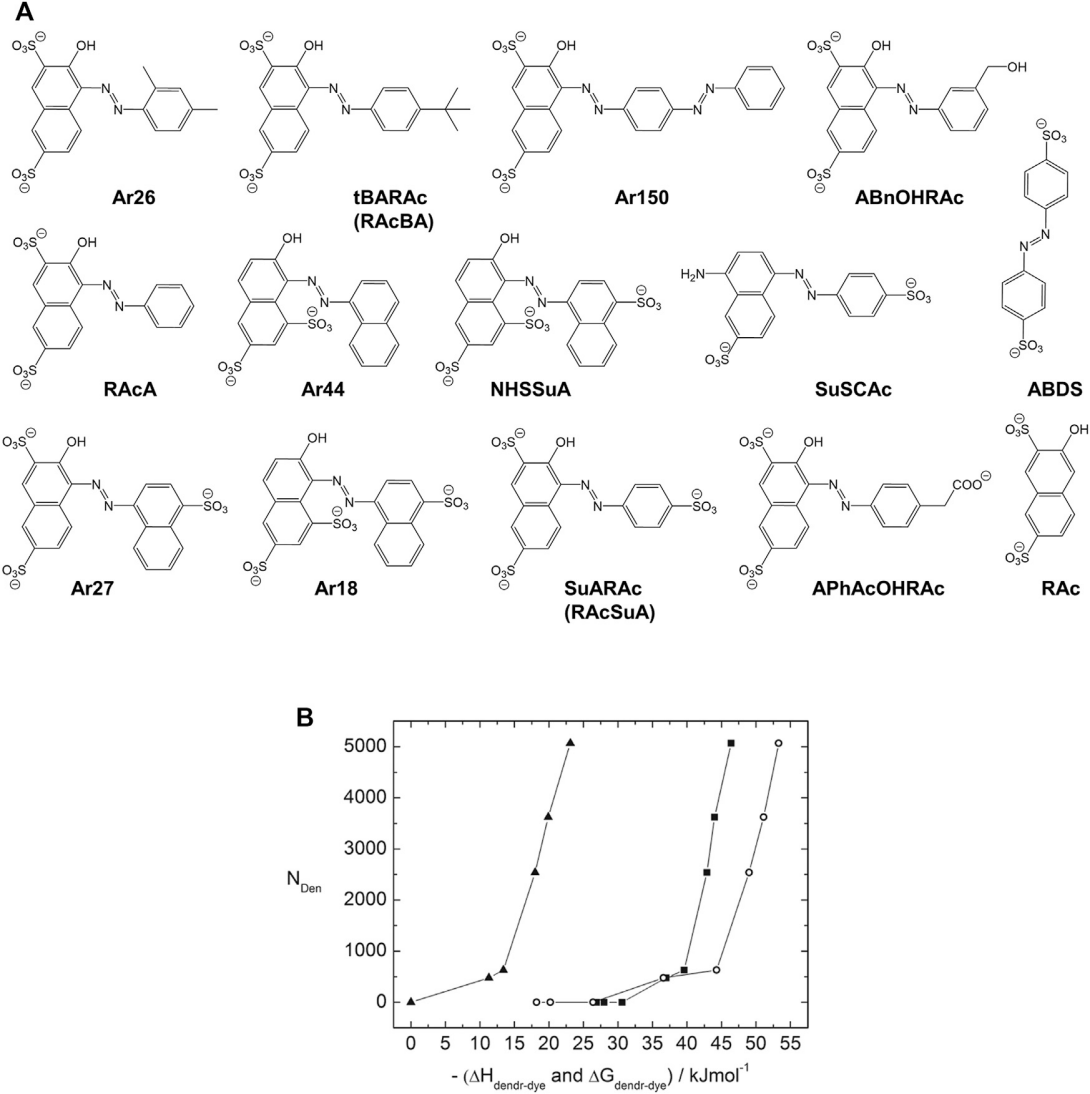
*Thermodynamic study of a dendrimer-dye model system for electrostatic self-assembly in solution, elucidating the nano-objects’ size control:* **(A)** *Structural formulae of structurally related divalent and trivalent azo dyes;* **(B)** *Dendrimer aggregation number N*_*Den*_ *in dependence on the dendrimer-dye interaction free energy ΔG*_dendrimer-dye_ (*squares*) *and dendrimer-dye interaction enthalpy ΔH*_*dendrimer-dye*_ (*open circles*) *and dye-dye interaction free energy ΔG*_*dye_dye*_ (*triangles*) *for divalent dyes* (Willerich and Gröhn, 2011a). *Reprinted with permission. Copyright © 2011 American Chemical Society.*

The Gröhn group demonstrated that the assembly can be further understood from thermodynamic considerations by a systematic model study using the mentioned family of dyes with a variety of di- and trivalent azo-dyes containing sulfonate-groups at different positions (Figure 11A) (Willerich and Gröhn, 2008; Willerich et al., 2009; Willerich and Gröhn, 2011a) Isothermal titration calorimetry (ITC) allows the tracking of the heat evolution during the assembly formation process for the dendrimer-dye interactions. The enthalpies ΔH_dendrimer-dye_, as well as the stoichiometries, result directly from the titration experiments while fitting the titration curve yields the binding equilibrium constants K_dednrimer-dye_. The free energy ΔG is

ΔG = −RT ⋅lnK (2)

and from ΔH and ΔG, ΔS is accessible as well. Notably, the process of the dye binding to the polyelectrolyte is undeniably exothermic, for example, with about ΔH = −3,000 kJ/mol per G4 dendrimer, that is, for the binding of 62 Ar26 molecules (slightly understoichiometric), corresponding to ΔH = −45 kJ/mol per dye molecule binding to the dendrimer. This is remarkable as it is in contrast to polyelectrolyte aggregation with multivalent counterions, which is often endothermic and entropically driven. (Sinn et al., 2004) Considering various dyes, a gain of a free energy gain of at least ΔG ≈ −32 kJ mol per dye molecule is necessary for the interconnection of dendrimer and dye.

The free energy of the assembly formation can then be compared to the free energy of the dye-dye interaction. For those dyes that do self-associate at concentrations much higher than the ones used for the assembly here, thermodynamic parameters on the dye-dye interaction can be extricated from dye dilution experiments. Correspondingly, a comparison ITC experiment on the association of the dendrimer with a non-aromatic disulfonate — which leads to a rather undefined aggregation with broad size distribution in contrast to the aromatic counterion case — yields information on the electrostatic interaction. It turns out that the electrostatic interaction accounts for about one third and the dye-dye π-π interaction for about two thirds of the interaction enthalpy, for example ΔH_dye-dye_ = −30 kJ/mol and ΔH_electrostatic_ = −15 kJ/mol. For all dyes, it is:

$$\Delta G_{dendrimer-dye}\;=\;\Delta G_{dye-dye}\;+\;\Delta G_{electrostatic}$$ (3)

Thus, also quantitatively, the interplay of the two interaction forces, π-π and electrostatic interaction, has been confirmed. Furthermore, the aggregation number N_Den_ derived from scattering experiments can be related to the thermodynamic results, as shown in Figure 11B. For all the dyes together, one mastercurve of N_Den_ versus ΔG_dendrimer-dye_ (and likewise versus ΔH_dendrimer-dye_ and ΔG_dye_dye_) results. Thus, based on the mastercurve and if the ΔG_electrostatic_ is approached to only depend on valence, one can predict the aggregation number of a dendrimer-dye assembly with a new divalent dye with which one has not made any polyelectrolyte assemblies before, if one only determines information on ΔG_dye_dye_, i.e. from spectroscopy.

Moreover, the free energy for the self-assembly can be calculated by

$$\Delta G_{dendrimer-dye}\;=\;\Delta G_{electrostatic\;}\;+\;\Delta G_{secondary\;interactions}\;=\;\Delta G_{dendrimer-sulfonate\;groups\;}+\;\Delta G_{dye-dye}$$ (4)

In equilibrium it is

$$\Delta G_{attraction\;}=\;\Delta G_{repulsion}$$ (5)

That is when regarding all attractive and repulsive contributions of the association process:

$$N_{Den}\;N_{dye}\;\left(\Delta G_{electrostatic}\;+\;\Delta G_{dye-dye}\right)=\frac{q_{dendrimer}^{2}}{4\pi \varepsilon_{0}\varepsilon_{R}R_{H-den}}\;\cdot \sum_{i=1}^{N_{Den}-1}\left(\;\frac{i}{\sqrt[{3}]{i}+\;1}exp\left(-\kappa \;\left(\sqrt[{3}]{i}+\;1\right)\;R_{H-den}\right)\right)$$ (6)

or

$$N_{Den}\;N_{dye}\;\Delta G_{dendrimer-dye}\;=\;\frac{q_{dendrimer}^{2}}{4\pi \varepsilon_{0}\varepsilon_{R}R_{H-den}}\cdot \sum_{i=1}^{N_{Den}-1}\left(\;\frac{i}{\sqrt[{3}]{i}+\;1}exp\left(-\kappa \;\left(\sqrt[{3}]{i}+\;1\right)\;R_{H-den}\right)\right)\;$$ (7)

As a solution, Eq. 6 yields one finite aggregation number N_Den_ for each ΔG_dye-dye_, i.e. for each dye, thus confirming that finite-size assemblies with a certain aggregation number (size) form. Furthermore, Eq. 7 makes it possible to calculate the aggregation number N_Den_ as a function of the free energy of the assembly formation ΔG_dendrimer-dye_, revealing an increase of the aggregation number with increasing ΔG_dendrimer-dye_ in correspondence with the experimental result (Figure 11B). Thus, the aggregation number N_Den_ is a thermodynamically favored value and does not increase infinitely, which would lead to precipitation. Of course, the calculation is far too simple, as even spherical polyelectrolytes in solution only cannot fully be described by a simple Debye-Hückel approach (Antonietti et al., 2000; Gröhn and Antonietti, 2000). However, it does give a good approximation for this general consideration and the principle outcome would not change with a more refined expression for the electrostatic interaction.

Beyond this model system, systems with other polyelectrolytes and other dyes yield corresponding results: the assembly formation is predominantly enthalpically driven with a building block and loading ratio controlled assembly size that reflects in a ΔG-determined aggregation number (Willerich and Gröhn, 2011b; Willerich et al., 2011; Kutz et al., 2017). Here, the additive nature of the attractive forces becomes evident also for other cases, demonstrating its general character. Interestingly, when attractive Hamaker interaction in-between gold nanoparticles that are included inside of the dendrimers occurs, it represents the third contribution in addition to electrostatics and π-π interactions:

$$\Delta G_{dendrimer-dye\;}=\;\Delta G_{electrostatic\;interactions}\;+\;\Delta G_{dye-dye}\;+\;\Delta G_{Hamaker}$$ (8)

such that due to the increased ΔG_dendrimer-dye_ still well-defined assemblies form that are larger than with the dendrimers only (Düring et al., 2013). Likewise, the approach holds when hydrogen bonding occurs (Kutz et al., 2017).

**FIGURE 12:**
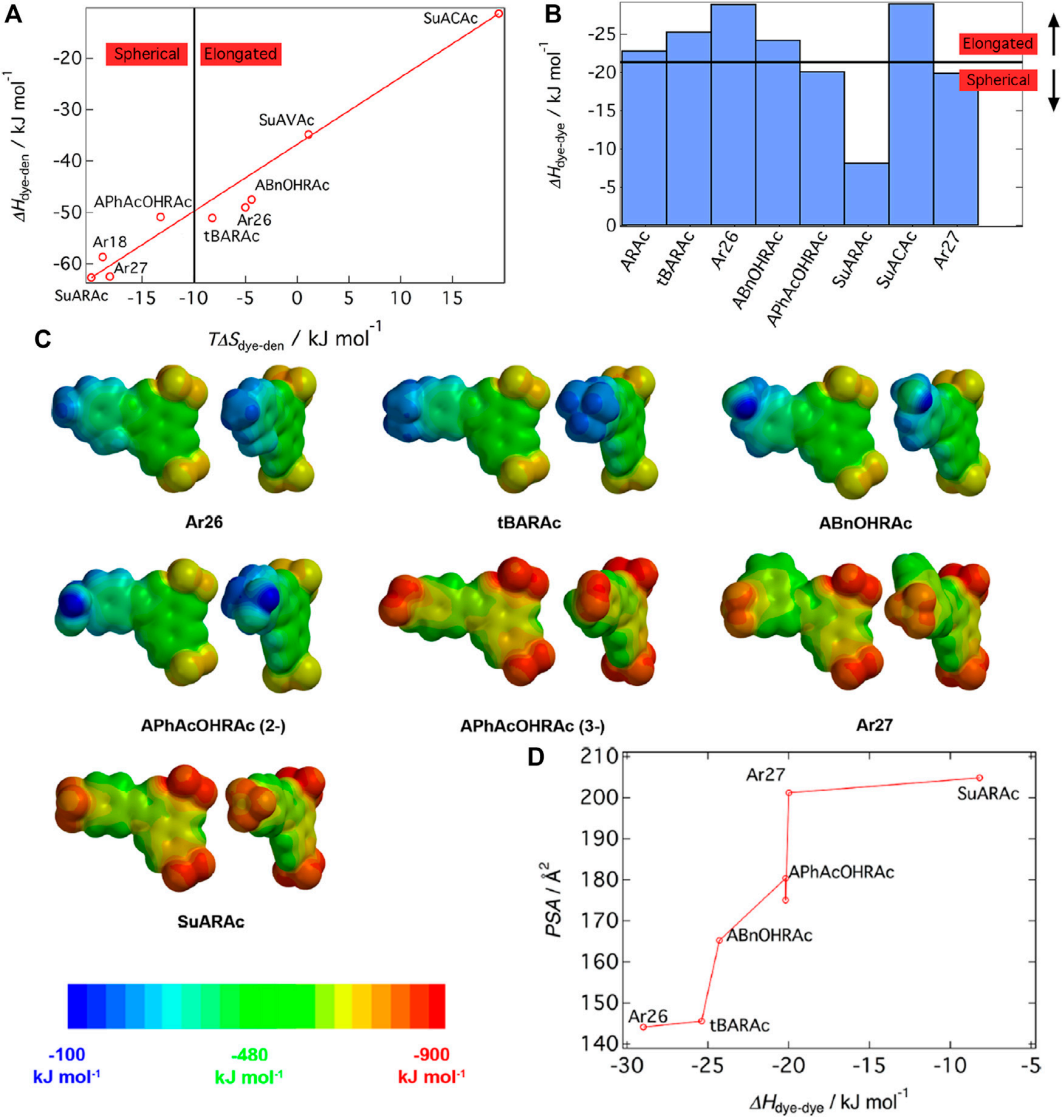
Elucidating the nanoscale shape control in electrostatic self-assembly by an experimental thermodynamics study: **(A)** Enthalpy-entropy relation for dye-dendrimer interaction; **(B)** Assembly symmetry depending on dye-dye interaction enthalpy; **(C)** Electrostatic potential at the molecular surface for the dye molecules from DFT calculation: each top view **(left)** and front view **(right)**; **(D)** masterplot of the polar surface area of the molecules as a function of ΔH_dye−dye_ (Mariani et al., 2016a). Reprinted with permission. Copyright ^©^ 2015 American Chemical Society.

While the thermodynamic consideration thus revealed that the free energy ΔG determines the aggregation number and thereby, the size of the nano-objects, it is imperative to understand how the various shapes of the assemblies are encoded. Can thermodynamics also help understand particle shape? Again, for the same model system, it could be elucidated out that the entropy-enthalpy balance determines the nanoparticle form, as shown in Figure 12a. The Figure displays the so-called effect of entropy−enthalpy compensation for dendrimer-dye assembly. The effect describes that a large part of an enthalpic gain is compensated by entropic effects TΔS. The enthalpy ΔH_dendrimer-dye_ increases with increasing TΔS_dendrimer-dye_. with the factor of 1.3. An entropy exchange of TΔS = −10 kJ/mol represents the threshold for the shape change. Moreover, the contributions can be separated here, and among divalent dyes with all having approximately the same electrostatic interaction, the nanoscale particle shape depends on the dye-dye interaction parameters. A ΔH_dye-dye_ > −21 kJ/mol (less negative ΔH_dye-dye_) leads to spherical particles, while in contrast, more negative ΔH_dye-dye_ values facilitate the formation of elongated, anisotropic particles, as depicted in Figure 12B (Mariani et al., 2016a). This means that a strong π−π contribution increases the sizes as well as the anisotropic character of dendrimer-dye particles, both of which have been quantified here.

After having demonstrated this remarkable relation of thermodynamics and nanoparticle shape, it was then important to again determine thermodynamics and how it can be related to molecular properties. Molecular modeling of the dye molecules by DFT calculations enables simulating the electrostatic potential at the molecular surface and various related parameters such as the dipole moment. Results for a variety of dye molecules are pictured in Figure 12C, showing that it is the polar surface area (PSA) of the dye molecules that relates to the ΔH_dye-dye_ in a master curve (Figure 12D). Thus, the molecular property has been defined that determines the association thermodynamics: the polar surface area.

For the same aromatic backbone with differing substituents, a higher polar surface area diminishes the π-π driven dye-dye interaction enthalpy. Interestingly, this very fundamental relationship on the influence of substituents on the π-π interaction had not been reported before. In the study of the Gröhn group, it was further related to the inter-dye stacking distance and tilt angle, which we skip to discuss here (Mariani et al., 2016a). At this point, it is important to emphasize the finding that thermodynamics serves as a key in understanding the nanoscale structure formation. It has been elucidated how thermodynamics relates to the resulting nanoscale architecture — ΔG determines the size, ΔH/ΔS determines the shape — and how in turn molecular properties determine the thermodynamics. In combination, this also leads to a masterplot of particle anisotropy versus PSA (Mariani et al., 2016a).

Thus, for this model system, an impressive understanding of structure formation and structure-directing principles have been developed. Different studies that access self-assembly beyond classical micelles in depth are several theoretical considerations by Douglas et al. Recently, they pointed out that it is a diverse range of supramolecular assembly processes that arise from the competition between directional and isotropic intermolecular interactions and focused on the nature of the self-assembly of spherical charged particles with competing van der Waals interactions bound to cylindrical surfaces, as a model system (Srebnik and Douglas, 2011). Glotzer et al. introduced a different theoretical approach by using building blocks with a highly complex shape. They predicted structures of different shapes formed of anisotropic building-blocks by calculating the symmetry of ordered arrays produced from these building blocks (Glotzer and Solomon, 2007; Damasceno et al., 2012). However, there are many more complex architectures where a full understanding of the structure-directing effects is still missing. This is not surprising, given that the whole field of building defined structures by electrostatic self-assembly is so much younger than the investigation of amphiphilic or amphiphilic-analogous concepts. At the same time, it is most crucial to develop such fundamental understanding from in-depth model studies to be able to exploit the capacity that lies in the versatile electrostatic self-assembly for functionality, such as, for example, in photocatalysis and switching for solar energy conversion and drug delivery, as will be discussed below.

## 8 Electrostatic Nanotemplating to Create Organic-Inorganic Hybrid Structures

**FIGURE 13:**
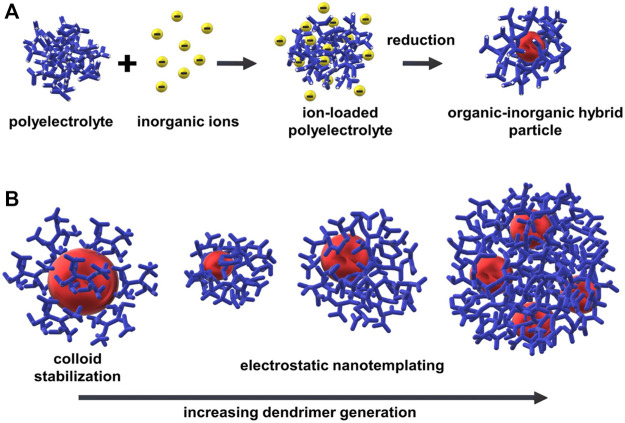
Electrostatic nanotemplating of metal nanoparticles in a polyelectrolyte: **(A)** Scheme of the principle, for example, synthesis of gold nanoparticles within a high-generation polyelectrolyte dendrimer; **(B)** Influence of stabilizing mechanism and hybrid particle morphology on dendrimer generation: Colloid stabilization for G2-G4, electrostatic nanotemplating for G6-G10 (not all dendrimer branches of the dendrimers of high generations (>G4) are shown for better visibility).

Self-assembly of oppositely charged ions can also be exploited for the formation of organic-inorganic hybrid nanostructures by the approach of “electrostatic nanotemplating.” The principle, as shown in Figures 13A, is to first accumulate precursor ions as counterions in and around a polyelectrolyte in an aqueous solution and then, in a second step, by chemical reaction generate inorganic nanoparticles in the inside of the polyelectrolyte.

**FIGURE 14:**
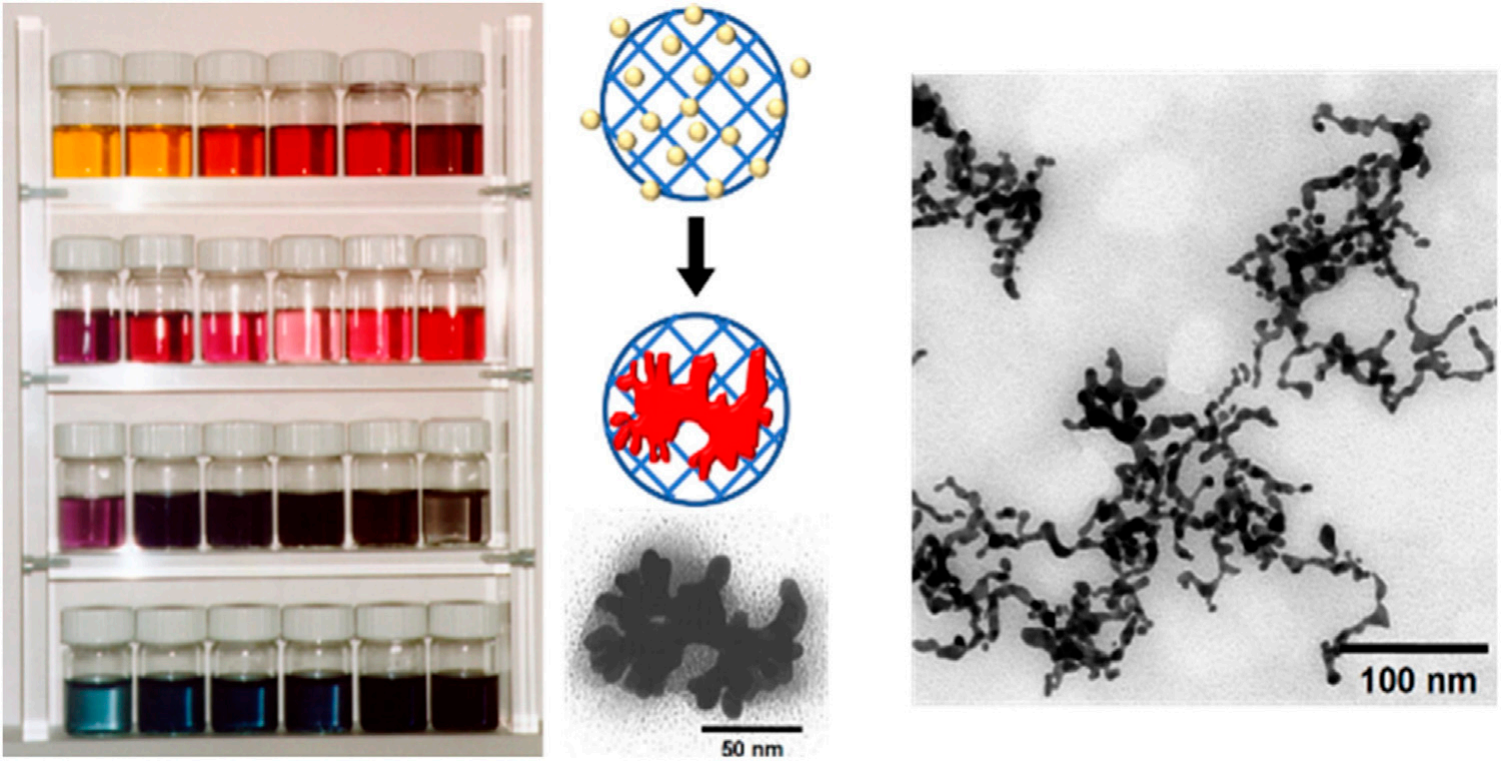
Electrostatic nanotemplating of gold in poly (styrene sulfonate) microgels; left: photograph of gold colloids prepared in microgels templates with varying size and cross-linking density. The different colors of the solutions indicate the adjustable range of gold nanoparticle sizes and morphologies; middle: schematic depiction of gold formation from Au-ions inside the microgel and TEM of a single gold-particle with nugget-like morphology with a microgel; right: TEM of threadlike gold within interconnected microgels (Antonietti et al., 1997). Reprinted with permission. Copyright ^©^ 1997 by WILEY-VCH Verlag GmbH, Germany.

Electrostatic nanotemplating was first described based on the formation of gold and iron oxide colloids with unusual shapes in polyelectrolyte microgels by Antonietti, Gröhn, and Bronstein, as shown in Figure 14 (Antonietti et al., 1997). The gold morphology could be influenced by the degree of crosslinking of the microgel, the loading ratio, and the reducing agent. The principle has since been applied to a wide variety of polyelectrolytes. In higher generation polyelectrolyte dendrimers, as shown by Gröhn and Amis, individual gold nanoparticles of thermodynamically defined size could be prepared and the hybrid particles were characterized in detail by small-angle scattering, electron microscopy, and spectroscopy (Gröhn et al., 2000). In a model system, the dendrimers in an aqueous solution were mixed with HAuCl_4_ and simultaneously protonated while AuCl_4_^−^ ions form the negative counterions to the cationic dendrimer macroion. The metal ions first accumulate in and around the polyelectrolyte dendrimers so that metal nanoclusters in the inside of the dendrimer form under the second step during the reduction of the metal ions. Here, in contrast to the microgel case, the nanoparticle size can be controlled by a “fixed loading”; that is, the ions added per dendrimer form one particle: For example, 4 nm gold particles consisting of about 2048 gold atoms in generation 9 (G9) dendrimer, 3.5 nm gold particles consisting of about 1,024 gold atoms in G8 and 2.5 nm particles comprising about 512 gold atoms in G7. Interestingly, for the G10 dendrimer, with the same slow reduction, about four 3 nm gold particles form within one dendrimer. Here, the space available and the flexibility are no longer sufficient to allow for the formation of one even larger particle. Thus, the polyelectrolyte template determines the size of the inorganic nanoparticles and the overall hybrid structure. In contrast, for the small generation dendrimers, the dendrimers attach to the surface of a growing gold particle in a typically controlled colloid stabilization. This demonstrates the change in mechanism from colloid stabilization to polymer nanotemplating by simply changing the template structure (dendrimer generation) while keeping the chemistry fully constant (Figure 13B).

Such dendrimer hybrid particles have also been successfully used as biological markers and selective catalysts (Zhao and Crooks, 1999; Lesniak et al., 2005). Furthermore, multiple variants of exploiting the principle of electrostatic nanotemplating have been described, such as the preparation of metal, CdS, and magnetic colloids in different microgels (Biffis et al., 2003; Xu et al., 2003; Zhang et al., 2004), nanoparticles in pH-responsive microgels (Palioura et al., 2007), in spherical polyelectrolyte brushes (Lu et al., 2009), in temperature-sensitive microgels (Pich et al., 2004; Rubio-Retama et al., 2007; Lu and Ballauff, 2011), cylindrical brushes (Zheng et al., 2013), and modified dendrimers (Hedden et al., 2002). Also the colloid stabilization with low-generation dendrimers was found for different systems as well, e.g. the formation of CdSe quantum dots in poly (propylene imine) dendrimers (Hayakawa et al., 2003; Fahmi et al., 2009). Thus, multiple variations of the method are available, all of which yield very promising polyelectrolyte-functionalized nanoparticles in an aqueous solution.

**FIGURE 15:**
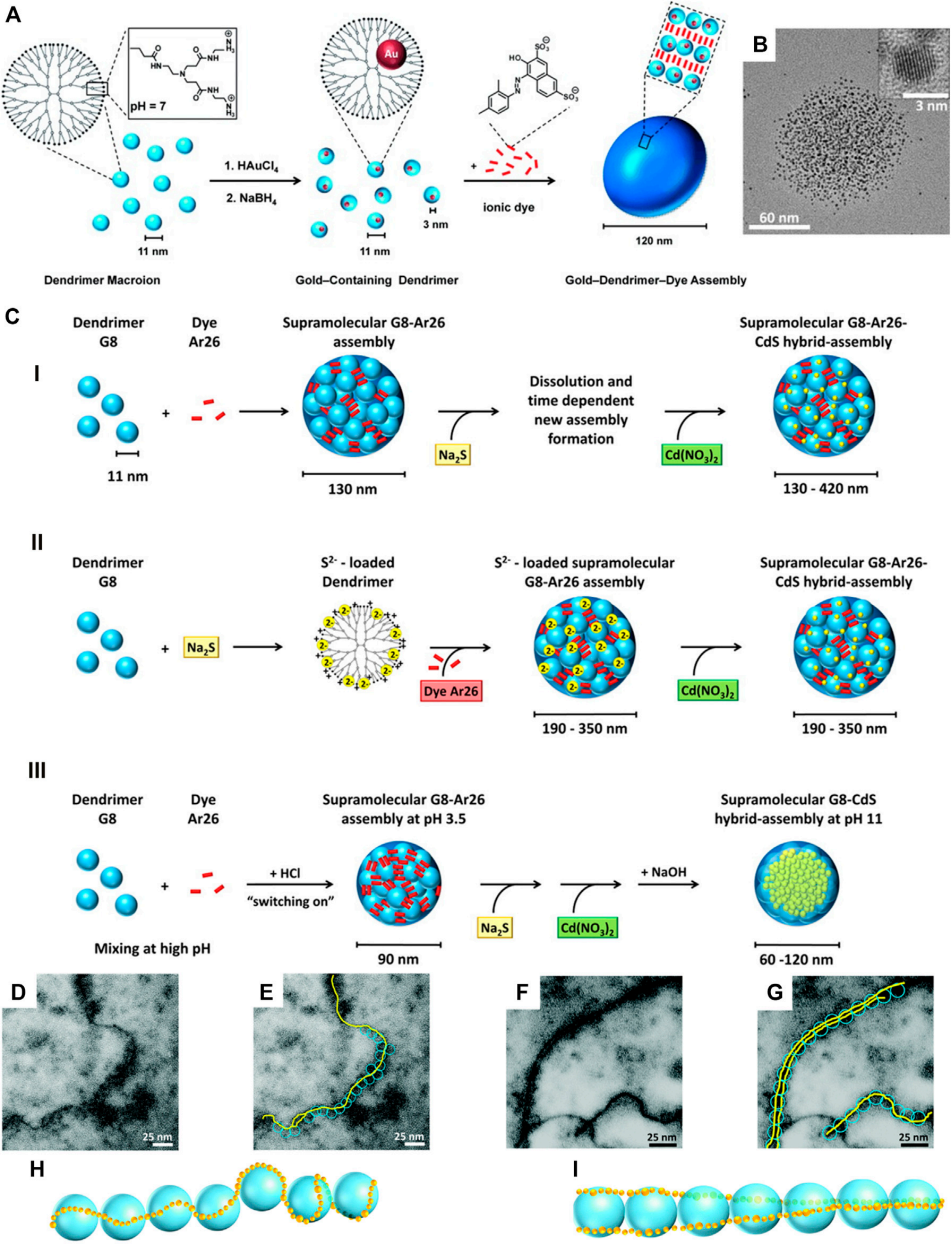
CdS and Au nanoparticles in supramolecular dendrimer−dye assemblies: **(A)** Electrostatic nanotemlating-electrostatic self-assembly approach; **(B)** TEM of a 100 nm god-dendrimer-dye assembly containing 3 nm gold particles prepared according to **(A)**; **(C)** overview of the electrostatic nanotemplating routes for CdS in dendrimer–dye assemblies; **(D–I)** CdS fibers with dendrimer G9: **(D,E)** TEM of single-fibers; **(F,G)** TEM of double-fibers, **(H,I)** proposed orientation of CdS (yellow) and dendrimer (blue) (Düring et al., 2013; Düring et al., 2015; Düring and Gröhn, 2016). Reprinted with permission. Copyright ^©^ 2013 WILEY-VCH Verlag GmbH and Co. KGaA, Weinheim and ^©^ The Royal Society of Chemistry 2016 and ^©^ 2015 American Chemical Society.

Furthermore, to establish a self-assembly route toward more complex multi-component functional structures, this method of electrostatic nanotemplating was recently combined with the electrostatic self-assembly in solution, as described above. In a first demonstration, nanoparticle-containing dendrimers were interconnected by oppositely charged dye molecules, yielding very well-defined 100 nm ternary gold-dendrimer-dye or CdS-dendrimer-dye hybrid particles (Figure 15) (Düring et al., 2013). Here it is particularly remarkable that 3 nm-sized CdS nanoparticles can be selectively prepared in an aqueous solution via a thermodynamic control. The CdS nanoparticles form within the polymer, serving two purposes: 1) The polymer stabilizes the nanoparticles in the aqueous solution (without participating), and 2) it does this in a way where it embeds the nanoparticles but does not fully cover their surface. The nanoparticle surface remains accessible, i.e. the nanoparticles can still be used for catalytic reactions that occur on their surfaces. Via fluorescence quenching, it could be demonstrated that a functional connection of the inorganic nanoparticle and the organic dye molecules is not hindered by the polyelectrolyte.

The systems can also be designed such that interconnection of polyelectrolytes occurs in the final hybrid structure. Recently, the formation of CdS mono- and double-nanowires with G9 PAMAM dendrimers was presented (Figure 15 bottom). An interplay involving Hamaker attraction, coordination, steric and dipole effects in addition to electrostatics was discussed to understand the structure formation (Düring and Gröhn, 2016). Finally, an even larger structural variety becomes available when first building an organic supramolecular structure by electrostatic self-assembly and secondly use remaining charges for an electrostatic nanotemplating within this suprastructure (Düring et al., 2015; Düring et al., 2017a). When using a light-responsive dye, it is also possible to switch the size and compactness of organic-inorganic hybrid structure such as the CdS–dendrimer fibers via light irradiation (Düring and Gröhn, 2016). Thus, the combination of electrostatic self-assembly and electrostatic nanotemplating opens an important field for the design of targeted functional and triggerable hybrid structures via a tool-box principle.

While the structure formation by electrostatic nanotemplating as such is well understood, as is the electrostatic self-assembly of hybrid particles performed by electrostatic nanotemplating, the formation of the inorganic material within the dynamic self-assembled organic nanostructure represents more complex scenario with various effects interplaying, such that a real prediction of the hybrid structure is not yet possible.

**FIGURE 16:**
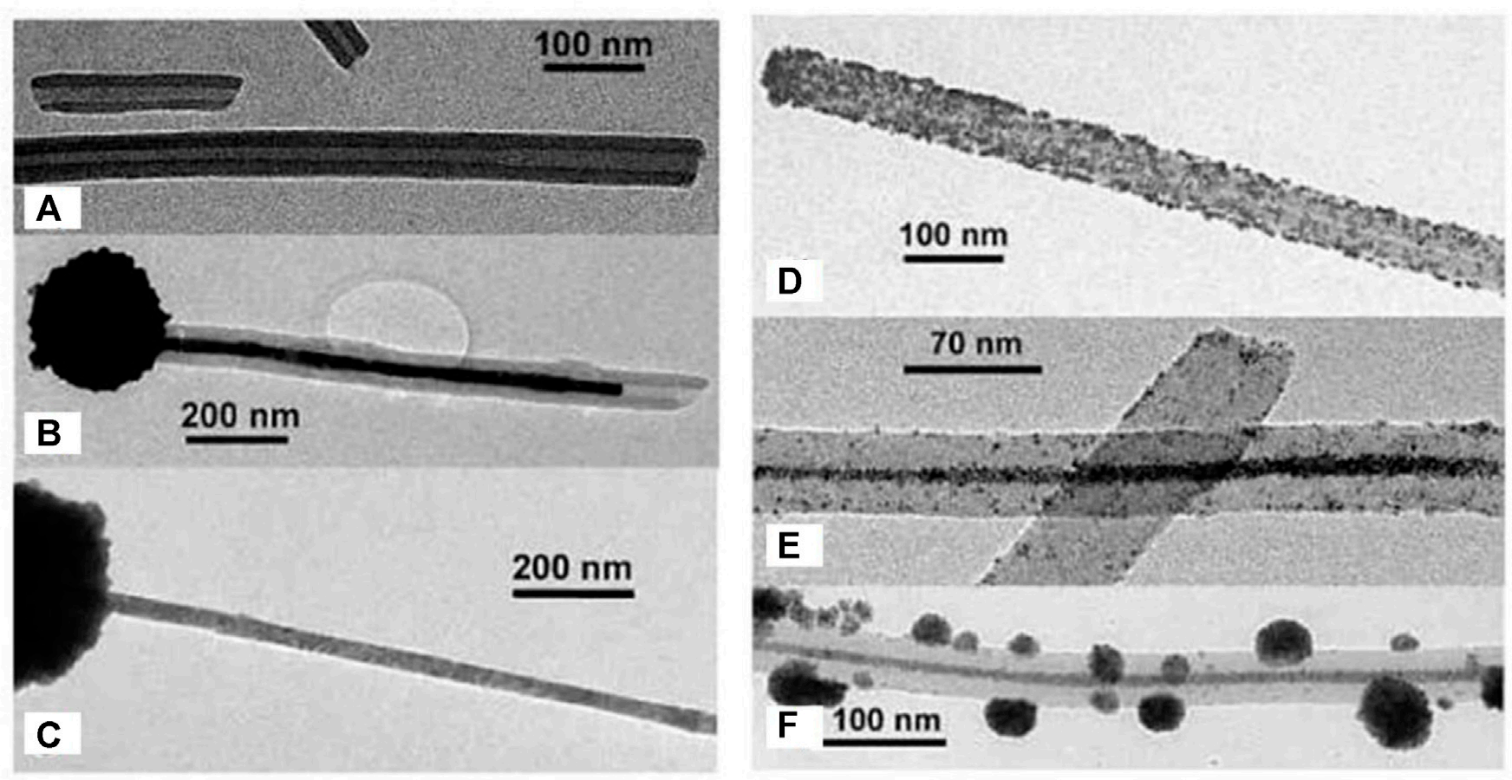
TEM of two-porphyrin nanotubes used as templates for noble metals: **(A)** porphyrin nanotubes; **(B)** porphyrin nanotube with gold nanostructure obtained by photoreduction; **(C)** free-standing gold wire obtained after the porphyrin tube has been dissolved away; **(D)** porphyrin nanotube with Pt nanoparticles distributed mainly on the outside surface; **(E)** a long Pt dendrite in the core of the tube obtained at higher Pt-concentration; **(F)** later stage in the development with a Pt dendrite in the core and globular Pt dendrites on the outer surface of the nanotube (Wang et al., 2004a). Reprinted with permission. Copyright ^©^ 2004 American Chemical Society.

A different kind of hybrid structure was created from porphyrin-porphyrin assemblies of tetrakis (4-sulfonatophenyl) porphyrin (TPPS) diacid and a tin porphyrin Sn(OH) (H_2_O/OH) tetrakis (4-pyridinium) porphyrin (Wang et al., 2004a; Wang et al., 2004b). The porphyrins assemble electrostatically and form long hollow nanotube structures with a diameter of 50–70 nm (Figure 16). Upon irradiation of the assemblies, the porphyrins are then able to photo-reduce added Au(I) complexes to metallic gold. The gold is formed inside of the porphyrin needles and pin-like structures result. The photoreduction by the porphyrin-assemblies is possible for platinum as well, which is accumulated inside the nanotubes. At higher Pt-concentration, continuous metal wires are formed with particles on the outside. This hybrid organic-inorganic material is a photocatalyst for the hydrogen evolution reaction and generates H_2_. Similar tubular structures are formed by TPPS-porphyrin with a peptide by inducting J-aggregation of the porphyrins. Subsequently, the nanotubes are decorated with platinum-fibers inside by photoreduction. The structures also catalyze the generation of hydrogen (Liu K. et al., 2015).

## 9 Diverse Use of Electrostatic Interactions for Structure Formation

As has been shown in the previous sections, very different strategies to exploit electrostatic interactions for the formation of nano- or micro-structures have been established. While each strategy can again be performed with various charged building blocks, it also yields a certain type of structure, e.g. two-dimensional films, macroscopic 3D materials or nanoscale objects with certain sizes and shape in solution. Table 1 gives an overview on the different strategies and resulting structures, both for the more established routes for which we focus on their essence (Section 4), as well as newer approaches concerning which this review gives a comprehensive picture (nano-objects by spontaneous self-assembly in solution, Section 5–7). Details and references can be found in the respective sections given in the Table.

**TABLE 1:** Overview of different electrostatically self-assembled structures.

Self-assembled material	Building blocks		Resulting structures	Typical functionality	Section
Layer-by-layer deposition					
Multilayer film materials	Cationic and anionic polyelectrolytes, proteins, polymer particles, nanoparticles, clay platelets		Layered films	Coatings, sensors, solar cells	3
			Layered capsules	Smart drug transport	
Spontaneous self-assembly to macroscopic materials					
Polyelectrolyte-polyelectrolyte aggregates	Positively and negatively charged polyelectrolytes		Polyelectrolyte aggregates, often with broad size distribution or precipitating; exceptions: e.g. for low charge, special polyelectrolyte-architectures	Basic framework, drug delivery	4.1
Nanoparticle aggregates	Cationically and anionically modified inorganic nanoparticles		Macroscopic 3D materials, exceptions: aggregates solution	Model systems for DLVO theory	4.2
Polyelectrolyte-surfactant materials	Polyelectrolytes and oppositely charged surfactant		Macroscopic 3D materials with internal mesostructure	Super-hydrophobic coatings	4.3
			Exceptions: assemblies in solution	Light-harvesting antennae	6
Surfactant-dye materials	Ionic surfactants and oppositely charged dye molecules		Macroscopic 3D materials with internal mesostructure	Optical materials	4.4
Dye-dye assemblies	Cationic and anionic porphyrins		Micrometer-sized, defined crystalline structures	Photocatalysis	4.5
Spontaneous self-assembly with block copolymers					
Block-polyelectrolyte assemblies	Charged block copolymers with oppositely charged block copolymers, homo-polyelectrolytes or dendrimers		Defined multi-compartment micelle-like assemblies in solution or as macroscopic material, hydrogels	pH-driven drug delivery, tissue engineering, diagnostics	4.6
Spontaneous self-assembly to nano-structures in solution					
Nano-objects	Macroions	Structural counterions
	Linear polyelectrolytes, dendrimers, cylindrical brushes, proteins, enzymes, DNA, polyoxo-metalates, micelles	Stiff multivalent organic ions, multivalent azo dyes, porphyrins, rhodamines, spiropyrans, polyoxo-metalates	Defined assemblies, stable in solution with internal structure	Multi-stimuli-responsiveness, photocatalysis, solar energy conversion, transport, smart optical systems	5, 6
Organic-inorganic hybrid particles	Macroions and metal salts		Inorganic nanoparticles (semiconductor/metal) in polyelectrolyte matrix	Catalysis, optical applications, biomedical usage	8

## 10 Switching Nanostructure and Properties

One great advantage of self-assembly by non-covalent interactions is the possibility to trigger the structures through external forces. The significance of this process can be found in our bodies. Our ability to see is provided by the light-induced isomerization of 11-*cis*-retinal to all-*trans*-retinal (Wald, 1968). This finding by Wald, Hartline, and Granit received a Nobel Prize in 1967. In addition to making it possible to switch the structure and the properties of nanoparticles formed by self-assembly (Lehn, 1995; Balzani et al., 2000), this is also the case for the nano-assemblies synthesized by electrostatic self-assembly, something that has been focused on more recently. Multiple external triggers can be exploited such as pH, light irradiation, temperature, as well as electrochemical or magnetic stimuli to change in the size, shape, and photophysical properties of the assemblies. In the following, we will summarize and discuss the state of knowledge regarding switchable nano-objects based on electrostatic self-assembly.

### 10.1 pH-Switchability

Andrade et al. were one of the first to use the electrostatic interaction to form pH-dependent nanoparticles. They combined meso-tetrakis (*p*-sulfonatophenyl)porphyrin (TSPP) sodium salt with the human serum albumin and β-lactoglobulin, where the binding affinity strongly depended on the pH of the solution (Andrade and Costa, 2002).

**FIGURE 17:**
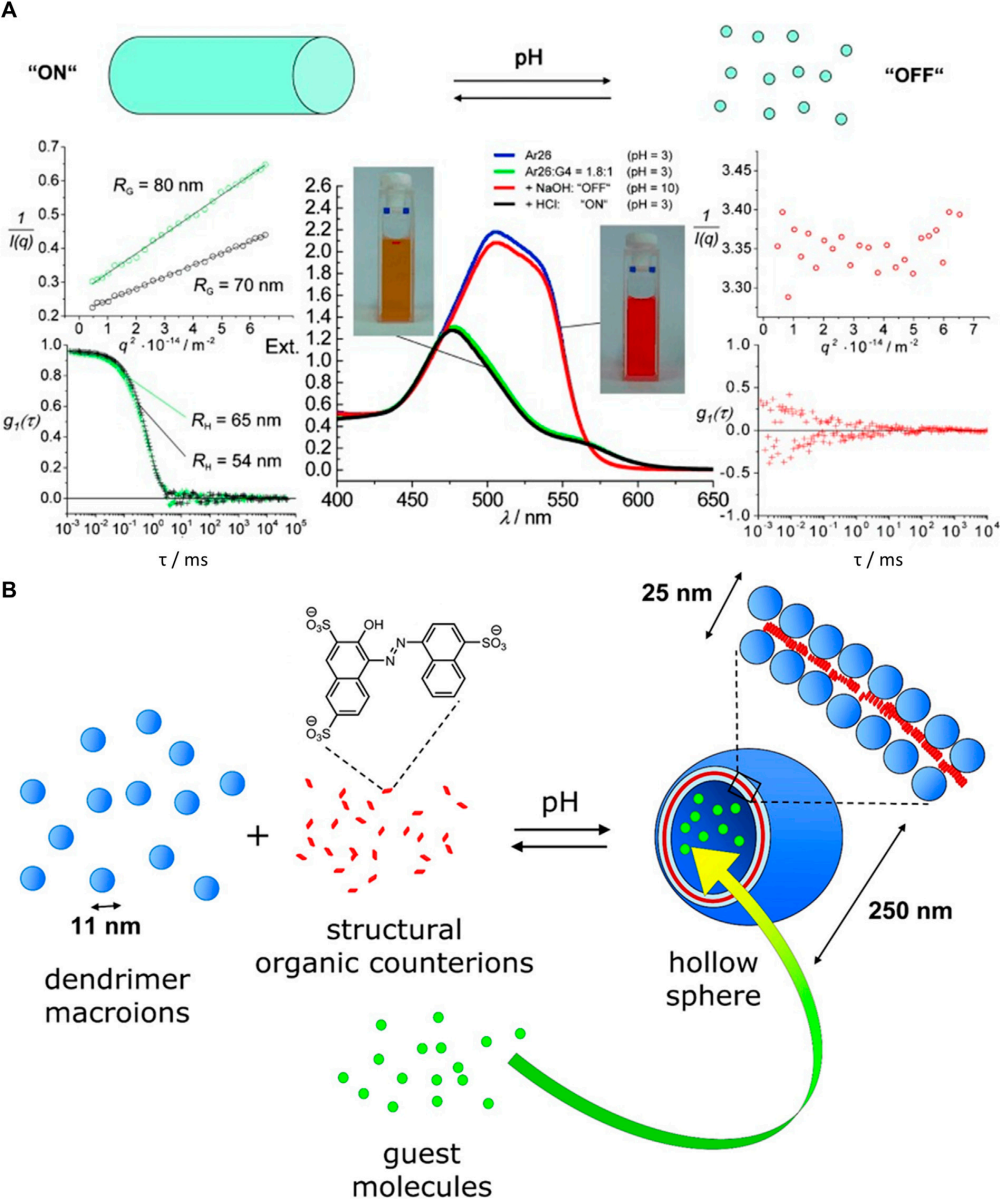
Switching “on” and “off” of electrostatically self-assembled structures by pH: **(A)** G4-Ar26 assemblies: “on” and “off” means aggregates and single dendrimers, respectively; center: UV/Vis spectra; left and right, static and dynamic light-scattering (Willerich and Gröhn, 2008), **(B)** Capsule formation and pH on-off switching of G8 dendrimer with Ar27 (Gröhn et al., 2010). Reprinted with permission. Copyright ^©^ 2008 and 2010 WILEY-VCH Verlag GmbH, Co. KGaA, Weinheim and The Royal Sociaty of Chemistry.

The Gröhn group demonstrated a pH switchable formation of electrostatically self-assembled nanoparticles in solution, i.e. switching between building blocks and assemblies as can be seen in Figure 17 (Willerich and Gröhn, 2008). They used fourth-generation (G4) poly (amidoamine) dendrimer as the cationic polyelectrolyte and Ar26 as the counterion. At a pH-value of pH = 6.5 stable aggregates form at a loading ratio of anionic dye sulfonate groups to primary dendrimer amine groups of 4:1 and 0.9:1, while at a pH = 3.5 the particles are only stable from 2:1 to 1.4:1. This can be understood with the higher degree of protonation of the dendrimer, which has about twice as many charges at pH = 3.5 as compared to pH = 6.5. Upon increasing the loading ratio, more counterions bind electrostatically to the polyelectrolyte, while π-π interactions between dye molecules occur at the same time, leading to an increased size. DLS revealed well-defined sizes in the range of 55 nm < R_H_ < 85 nm, while from SANS in combination with SLS it was deducted that the form of the assemblies is a cylindrical shape with a length of about 225 nm and a cross-section dimension of about 60 nm. By changing the pH from pH = 3.0 to pH = 10 the assemblies dissolve due to the deprotonation of the polyelectrolyte and the then missing electrostatic interaction. Upon subsequent addition of acid, the nanoparticles are again formed, and the accompanying switching back and forth can be repeated at least 10 times (Figure 17A).

This on-off switch can also be used to form nano-assemblies for drug release. Cationic G8 dendrimer with the trianionic azo dye Ar27 forms well-defined hollow capsules with a size of R_H_ = 130 nm (Figure 17B). Particularly interesting is the property that these hollow spheres can be dissolved by increasing the pH value. Furthermore, guest molecules can also be included in the inside of the carriers, as shown for fluorescently labeled peptides (Gröhn et al., 2010). It should be emphasized that these capsules are very different from amphiphilic vesicles. Here, two kinds of hydrophilic water-soluble molecules associate into hollow capsules due to the interplay of the above-mentioned interaction effects.

Later, to further investigate the influence of the pH on the self-assembly of cationic polyelectrolyte dendrimers and oppositely charged dyes, Gröhn et al. examined the shape of nano-assemblies comprising different anionic azo dyes with the G4 dendrimer at different pH-values (Mariani et al., 2016c). It was elucidated that the pH is a key trigger to tune the size, shape, and stability of the nano-assemblies of these building blocks. By combining the results of DLS and ζ-potential, the surface charge density was revealed to be the crucial parameter in the stability and size of the nanoparticles. At low pH, stable and well-defined nanoparticles are found when dye molecules can also bind to the ternary amines “inside” of the dendrimer. At higher pH values, the dye molecules can only bind to the primary amines and thus stabilize the particles less than at lower pH values. In the aforementioned system, for the Ar26 the pH-value triggers the structure from nanoscale cylinders with elliptical cross-sections with R_H_ = 600 nm to spherical nanoparticles with R_H_ = 57 nm (Mariani et al., 2016c). For another related but structurally different dye (ABnOHRA, disodium 4-((3-(hydroxymethyl) phenyl)diazenyl)-3-hydroxynaphthalene-2,7-disulfonate), the pH could be used as a trigger to switch between 2 μm long flexible cylinders and shorter (100–500 nm) stiff cylinders, both with well-defined size and shape. Here, the control of nanostructure size and shape again follows the thermodynamic structure encoding discussed above, where the pH changes the charge status of the dendrimer and by extension, the counterion/macroion charge ratio as well as the charge distribution, thus resulting in changes in the “structure” of the macroion.

**FIGURE 18:**
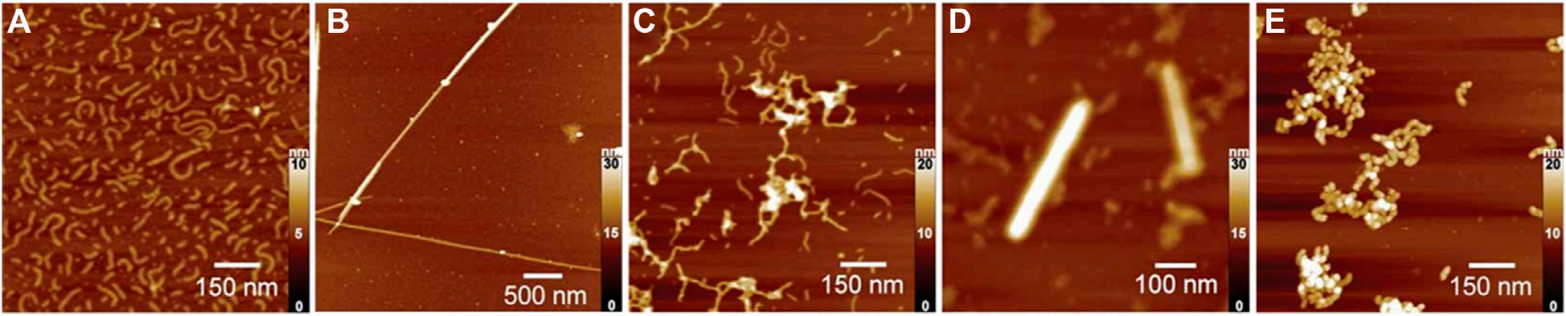
Cylindrical brush-nanorod-network switching in an electrostatically self-assembled polymer-brush porphyrin system; AFM: **(A)** wormlike PVP brushes, pH 7; **(B)** porphyrin nanorods, pH 2; **(C)** porphyrin-brush networks, pH 7; **(D)** nanorod-brush system, set from pH 7 to pH 2; **(E)** switched networks, pH 7; **(F)** nanorod-brush system, prepared at pH 2 (Ruthard et al., 2011b). Reprinted with permission. Copyright ^©^ 2011 WILEY-VCH Verlag GmbH and Co. KGaA, Weinheim.

Following that, Gröhn et al. also researched a system consisting of anionic TPPS, and cationic poly (2-vinylpyridine) cylindrical brushes, which yields a network-like structure (Ruthard et al., 2011b). Interaction occurs by electrostatic interaction of TPPS with the polyelectrolyte. At the same time, mutual π-π interactions occur in addition to ionic binding proven with UV/Vis spectroscopy, similar to the oppositely charged case discussed above. Altering the pH from pH = 7.0 to pH = 2.0, the network structure disassembles into porphyrin nanorods and individual polymer brushes which host TPPS J-aggregates and dianionic TPPS, which is illustrated in Figure 18. Readjusting the pH to pH = 7.0 the network structure reforms. Of further interest was switching from pH = 7.0 to pH = 3.5. Directly after switching, the shape and size of the assemblies remain unchanged, while the network structure disassembles to individual brushes after a few days. In this case, the porphyrin remains bound to the polymer but with a changed ratio of H/J-aggregates.

Helseth et al. also combined the cationic polyelectrolyte poly (allylamine hydrochloride) with the anionic dye pyranine as counterion, yielding a sheet-like colloidal structure (Helseth, 2012). The addition of NaOH leads to disaggregation of the initial complex and the formation of small spherical assemblies, which was accompanied by a change in the fluorescence of the particles.

Chen et al. adopted a different approach and combined the pH-switchable zwitterionic surfactant tetradecyldimethylamine oxide with an anionic POM (Lei et al., 2019a). The surfactant formed micelles itself in an aqueous solution. Upon the addition of POM, so-called “nanobelts” with a length up to micrometers and a width of 50–100 nm were formed with improved red-emitting luminescence. HRTEM images revealed an ordered lamellar packing structure with alternating POM and surfactant layers. Changing to a more basic solution stopped the fluorescence of the assemblies. Adjustable photoluminescent properties for electrostatically assembled structures in solution have also has been studied in other systems (Gong et al., 2016; Shen et al., 2019).

Another promising approach was introduced by Fathalla et al. (Fathalla and Sinatra, 2021). They synthesized benzoic acid-functionalized silica nanoparticles with nanopores and used them to form electrostatically assembled nano-structures with meso-tetrakis (N-methylpyridinium-4-yl) porphyrin. Here, the porphyrin acted as a capping agent for the nanopores of the silica nanoparticles, which lead to the nanopores being closed by the porphyrin. By decreasing the pH, the benzoic acid is protonated, the electrostatic interaction between the silica nanoparticles and the porphyrin is interrupted, and the nano-assemblies are dissolved.

### 10.2 Light Switchability

Compared to pH-responsive nano-assemblies, light switchable structures have recently gained more attention since light is a non-invasive and elegant external trigger that makes it possible to influence the properties of the nano-assemblies. These properties could be interesting for optical materials or switches in biological systems. As the switching through pH usually changes the charge status of one of the components, effects are relatively straight forward to understand for pH triggerable systems, whereas the effects in light responsiveness being accompanied by conformational changes and thereby geometry and/or polarity changes of one of the components, possibly in conjunction with the charge status, are much more complex.

*Cis-trans* isomerization of azobenzenes is the light-responsive reaction, which is most often used in nano-assemblies. They were first used to change the form and size of a structure by changing the secondary structure of a polymer (Goodman and Falxa, 1967). In nano-assemblies azo benzenes were first used to change vesicles into elongated structures upon irradiation (Sakai et al., 1999). The first light-responsive electrostatically self-assembled nanostructures based on azobenzenes were formed by Higashi et al. (Koga et al., 2007). They assembled an anionic azobenzenes derivate with a cationic β-sheet peptide into nanofibers. Upon irradiation and subsequent isomerization of the azo dye to its *cis*-state, the assemblies dissolved, and the particles reassembled upon isomerization of the azobenzene to its *trans*-state.

**FIGURE 19:**
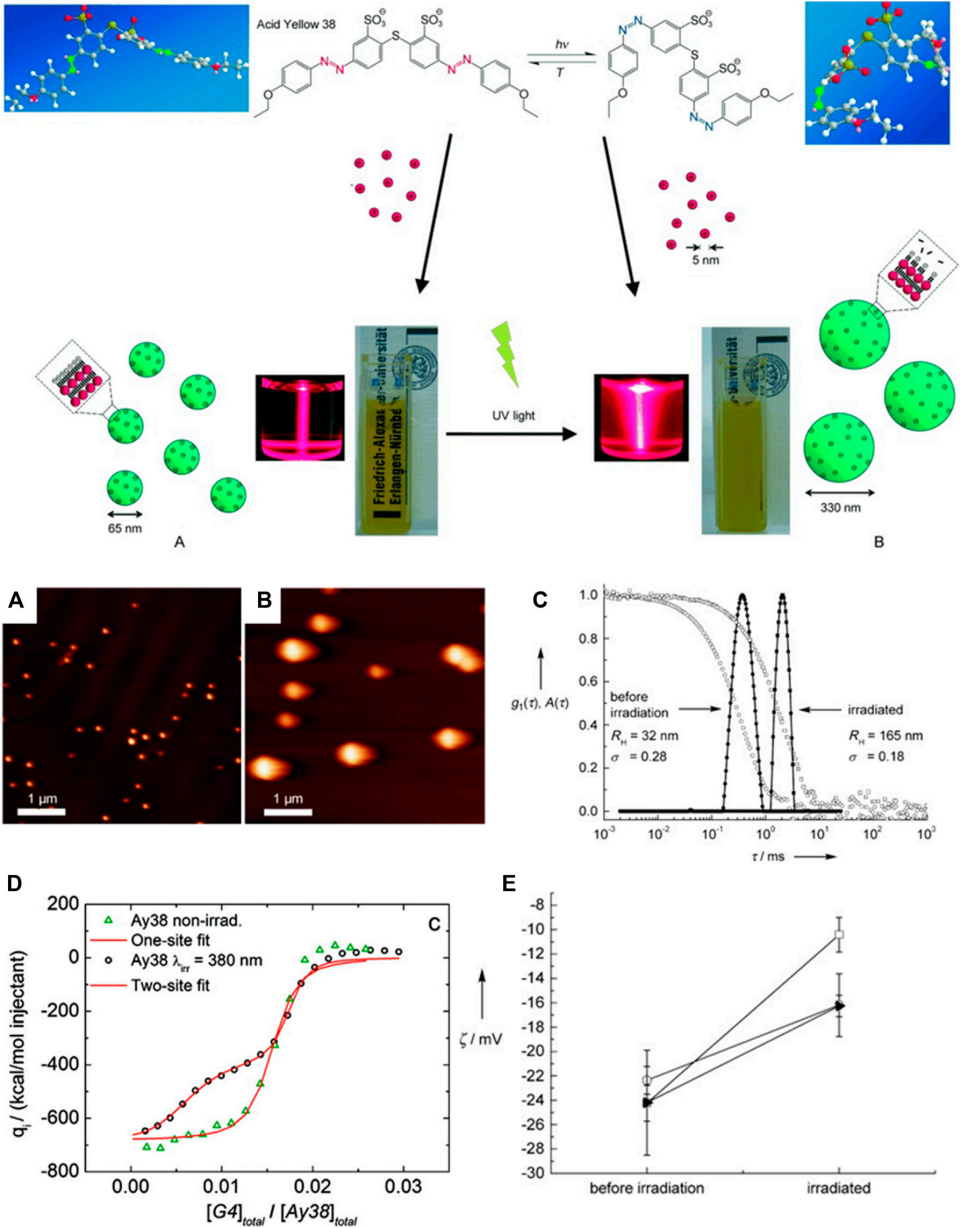
A light-switchable electrostatically self-assembled system with photo addressable particle size; top: schematic representation. Starting from a divalent anionic azobenzene dye and cationic dendrimeric macroions, self-assembled nanoparticles result **(A)**, which grow in size upon irradiation with UV light **(B)**; middle: AFM **(A)** before and **(B)** after UV irradiation and **(C)** corresponding DLS for a loading ratio l = 4.5; **(D)** Isothermal titration calorimetry revealing the different binding enthalpies of the cis and the trans dye isomer; **(E)** ζ-potential for dendrimer–dye assemblies before and after irradiation for different loading ratios confirming the charge density control of the particle size (Willerich and Gröhn, 2010; Willerich and Gröhn, 2011b). Reprinted with permission. Copyright ^©^ 2011 American Chemical Society and ^©^ 2010 WILEY-VCH Verlag GmbH and Co. KGaA, Weinheim.

While assembly/disassembly and gelation could be achieved via irradiation by light using different assembly concepts and triggers (for different triggers see below), it had remained a challenge to access a light-triggered nano-object size in solution. This was first achieved by the Gröhn group in 2010 (Willerich and Gröhn, 2010). The model system comprised azo-switchable anionic dyes in combination with a cationic dendrimer macroion in an aqueous solution (Figure 19). Unlike the electrostatically self-assembled structures of this group discussed above, the supramolecular particles with an excess of dye are of major interest for this size switchability in solution. Depending on the loading ratio between the sulfonate groups of the dye and the primary dendrimer amine groups, the narrowly distributed size of the spherical assemblies ranged between 31 nm ≤ R_H_ ≤ 52 nm.

Irradiation led to an increase in size to 37 nm ≤ R_H_ ≤ 251 nm, depending on the loading ratio within the dye-excess range, for example from R_H_ = 32 nm to R_H_ = 165 nm. The particles’ stability of the non-irradiated nano-assemblies originates from the excess and overbinding of the anionic azo dyes to the cationic polyelectrolyte via π−π interaction to electrostatically bound dye molecules. This leads to a high negative surface charge density, which stabilizes the particles electrostatically. The isomerization of the azo dye upon irradiation then causes an increase in the size of the nano-assemblies, since the surface charge density, as derived from ζ-potential measurements, is less expressed as compared to the assemblies with the *trans-*dye. Evidently, the *cis* dye after irradiation binds to a lesser extent than the *trans* isomer before irradiation. ITC, which is depicted in Figure 19, quantifies this effect and yields further insight. While the binding isotherm shows one step before irradiation, the dye-binding after irradiation clearly showed two steps and was described by a two set of site model, as *trans* and *cis*-isomers coexist after the irradiation. ITC measurements also show that the *trans* azo dye has a higher binding enthalpy and a higher binding equilibrium constant compared to the *cis* dye, in accordance with the ζ-potential results. This is because the *cis*-isomer is more polar and hydrophilic than the *trans*-isomer. Therefore, the affinity to the aqueous phase relative to the development of mutual π-π interaction is higher for the *cis-*isomer. Hence, upon irradiation, dye molecules detach from the nano-assemblies, thus reducing their negative surface charge density, which again causes the coagulation of nanoparticles into larger assemblies with a lower total surface that then are stable again in an aqueous solution. The difference in binding enthalpy of the *trans*-isomer and *cis*-isomer is pivotal to understand how much the size changes upon irradiation and can be exploited to adjust the size via the *cis/trans* ratio by choice of the irradiation wavelength (Willerich and Gröhn, 2011b).

The generality and versatility of this type of light switchable nano-objects obtained by electrostatic self-assembly were further demonstrated by the variation of the building blocks (Moldenhauer and Gröhn, 2013). In particular, different linear flexible cationic polyelectrolytes were combined with the same azo dye Ay38. Stable nanoparticles are formed, except for the loading ratio of *l* = 1.0, where the assemblies directly precipitate due to a missing electrostatic stabilization of the assemblies in solution. Irradiation for the samples with *l* < 1.0 leads to a decrease in size, which is different from the dendrimer system. At *l* > 2.0 irradiation led to an increase in size with again a similar cause as outlined for the dendrimer-Ay38 system. The surface charge density also plays a vital role for *l* < 1.0 and the most stable particles are found to be at an excess of positive charges, which is comparable to the (non-switchable) model system discussed in the previous section. However, light scattering and AFM measurement further showed that the changes upon irradiation here are attributed to a change in density as opposed to a change in aggregation number. Probably, the assemblies with the linear polyelectrolytes are looser and more flexible than the dendrimer assemblies, which is why shrinking represents an additional response possibility upon light irradiation. There are two reasons co-playing: One is the *cis*-form present after irradiation being more compact than the *trans*-form of the azo dye. The second reason is that the interaction before irradiation is more enthalpy-driven than entropically. After irradiation, the interaction is more entropically controlled, i.e. a larger gain in the entropy of water can account for entropy loss through polymer chain compaction.

Yu et al. used the electrostatic self-assembly to combine 4-ethyl-4´-(trimethylaminohexyloxy) azobenzene bromide with an original Keggin-type POM (Guo et al., 2016a). Upon the azo dye’s irradiation, the structure changes from a coral-like shape to smaller nanospheres. This behavior can be attributed to the changes in stacking matter and the hydrophobic interaction of the surfactant molecules upon irradiation. Similar research was also conducted by the group of Li (Xie et al., 2020).

**FIGURE 20:**
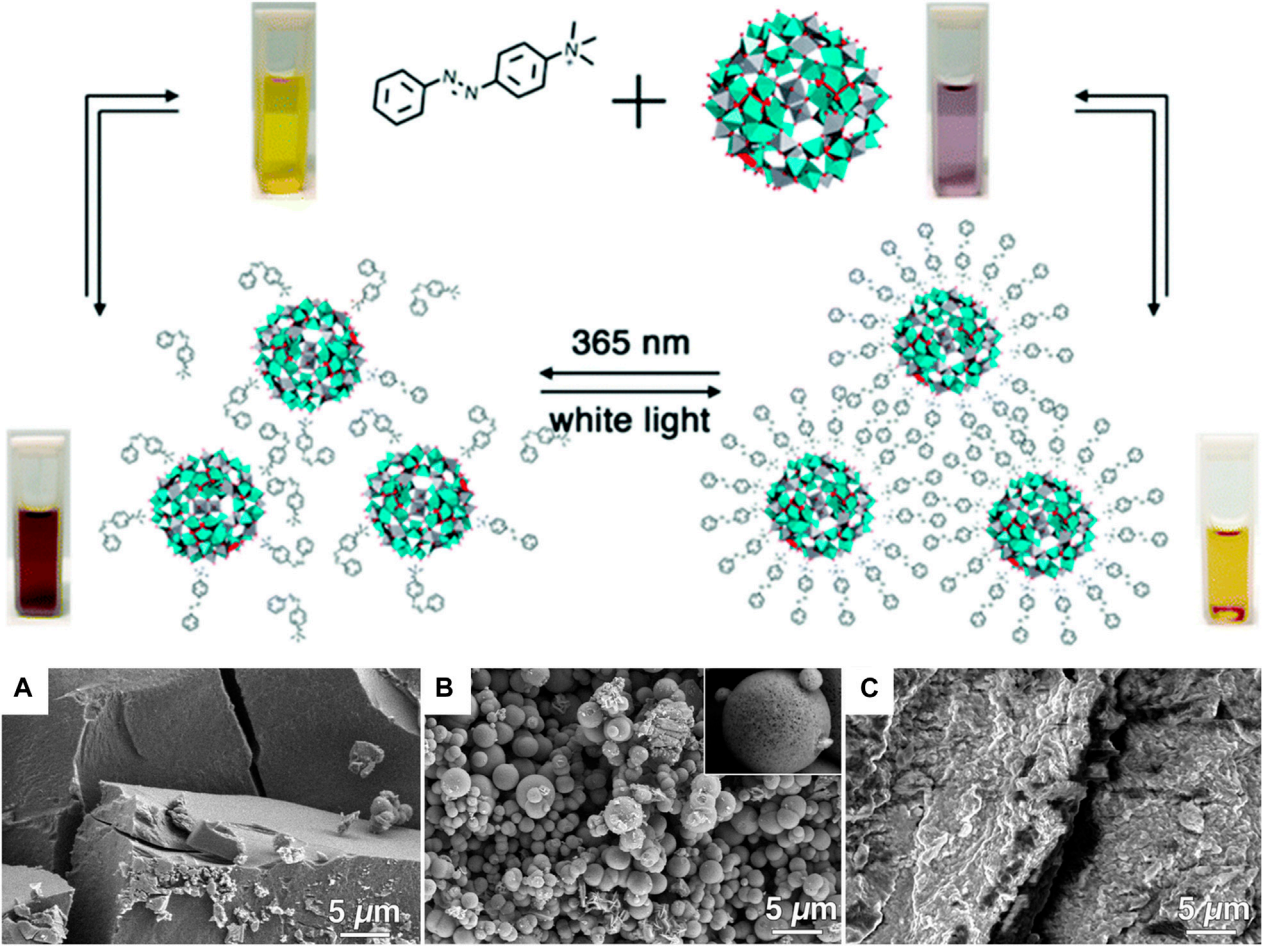
{Mo_72_V_30_}–azo complex with irradiation dependent interconnection; top: schematic representation; bottom: SEM of dry films prepared by drop-casting **(A)** {Mo_72_V_30_} before functionalization with azo molecules; **(B)** the {Mo_72_V_30_}-trans-azo hybrid structure; **(C)** {Mo_72_V_30_}-cis-azo obtained upon UV-light irradiation (365 nm) prior to drop-casting of the {Mo_72_V_30_}-trans-azo solution (Markiewicz et al., 2017). Reprinted with permission. Copyright ^©^ The Royal Society of Chemistry 2017.

In a different example for light-triggered assembly/dis-assembly by the group of Samori, they directly anchored a cationic azo benzene derivate to a spherical Keplerate-type POM via electrostatic self-assembly. Here, the assemblies form a spherical structure which, via π-π interaction, result in larger nanostructured aggregates they refer to as “supramolecules”, as can be seen in Figure 20 (Markiewicz et al., 2017). Dynamic light scattering showed a hydrodynamic radius of R_H_ = 77 nm. After irradiation, the self-assembled structures start to disappear and aggregate with a size of R_H_ = 9.5 nm.

**FIGURE 21:**
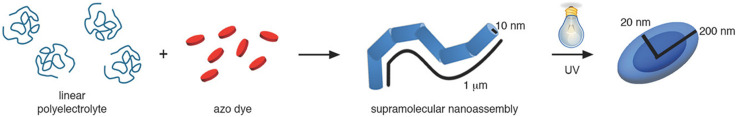
Light switchable nano-object shape by electrostatic self-assembly: An anionic azo dye (Ay38) and a linear flexible polyelectrolyte (PDADMA) for micrometer long thin fibers which compact into ellipsoids upon irradiation (Mariani et al., 2018). Reprinted with permission. Copyright ^©^ 2018 WILEY-VCH Verlag GmbH and Co. KGaA, Weinheim.

A light-triggerable particle shape via modern self-assembly approaches, beyond the classical micelle shapes, has represented another major desire. Again, electrostatic self-assembly in solution played a key role. In the first and impressive model system, the Gröhn group studied linear cationic polyelectrolytes with different azo dyes (Figure 21) (Mariani et al., 2018) Remarkably, PDADMAC in combination with Ay38, forms flexible cylinders with more than a micrometer length and an elliptical cross-section with a major axis R_maj_ = (14 ± 1) nm and a minor axis R_min_ = (5 ± 2) nm. Upon irradiation, the shape of the particles drastically changes into a core-shell ellipsoid which has a major axis R_maj_ = (200 ± 20) nm and a minor axis R_min_ = (20 ± 1) nm; the inner core has r_maj_ = (60 ± 5) nm and r_min_ = (10 ± 1) nm. With the help of UV/Vis spectroscopy, it was found out that the stacking angle changed upon irradiation from *β* = 60° to *β* = 90° suggesting that the observed change upon irradiation is connected to the change in the dye stacking configuration. This behavior can be understood with the different polarity of the two isomer states of the azo dye, which subsequently also influences the dye-dye stacking. The same experiment was carried out with a different polymer in form of poly (N-methyl-4-vinylpyridinium nitrate) and in this case, no change in shape upon irradiation was observed. One possible explanation is the additional π-π interaction, which can occur due to the aromatic ring on the polymer, which prevents a change in shape. To our knowledge, this was the first time that shape changes triggered by irradiation have been observed for electrostatically self-assembled nano-objects. In other words, assemblies are capable of converting light energy into “mechanical” energy, offering highly promising possibilities. As the electrostatic-based principle is general, it is versatile and bears potential in drug delivery or energy conversion.

**FIGURE 22:**
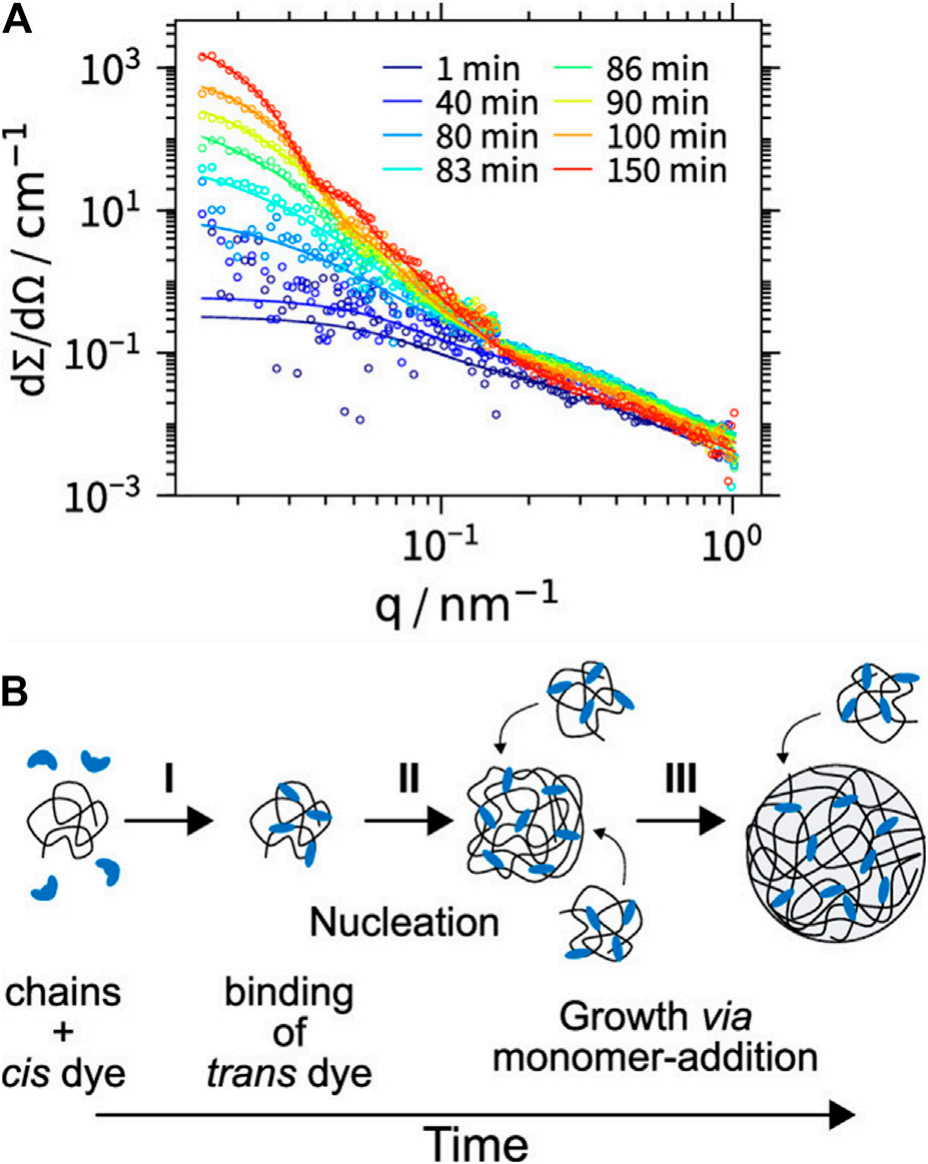
Study of the formation mechanism in light-responsive electrostatic self-assembly: **(A)** Selected SANS profiles of reforming PA–diAzoEt complexes after irradiation and reassembly at 45°C. The solid lines represent fits to the model of a mixture of free polymer chains and complexes; **(B)** Illustration of the mechanism of the self-assembly of the PA−diAzoEt complexes (Carl et al., 2020). Reprinted with permission. Copyright ^©^ 2019 American Chemical Society.

Recently, Huber and coworkers used a similar approach with the anionic polyelectrolyte poly (acrylate) and the *trans* isomer of a divalent diazophenol cation with a special focus on the rearrangement mechanism, as depicted in Figure 22. Well-defined spherical structures are formed in an aqueous solution of the order of R_H_ = 76 nm (Carl et al., 2020). These assemblies could be reversibly assembled and disassembled by using the isomerization of the azo dye. The size and structure also could be controlled by varying the temperature at the reassemble process. By light and neutron scattering, they revealed that the first step, namely the *cis*-*trans* isomerization, is the rate-limiting step. This includes the binding of the *trans*-azo dye to the anionic polyelectrolyte. When a specific number of the azo dye molecules interacts with the polyelectrolyte the chain becomes too hydrophobic, which results in nucleation. This leads to the second step taking place by a monomer-addition-mechanism. Here, the morphology of the aggregate changes from a loosely packed structure to a homogeneous and dense spherical aggregate. In the third step, these assemblies grow via a monomer addition mechanism. This is in contrast to the system of Gröhn et al. where, depending on the loading ratio, the irradiation to the *cis*-isomer only leads to either a decrease or increase in size. Furthermore, the samples here start to precipitate at a charge ratio higher than *l* > 1.0, while the assemblies from the group of Gröhn were stable in solution. This is possibly due to a difference in hydrophilicity of the two systems. While both studies on their own yield valuable insight into the respective system, the comparison clearly demonstrates the need for broader studies or the consideration of different investigations in conjunction, in order to gain a thorough understanding on the light-induced structural changes.

**FIGURE 23:**
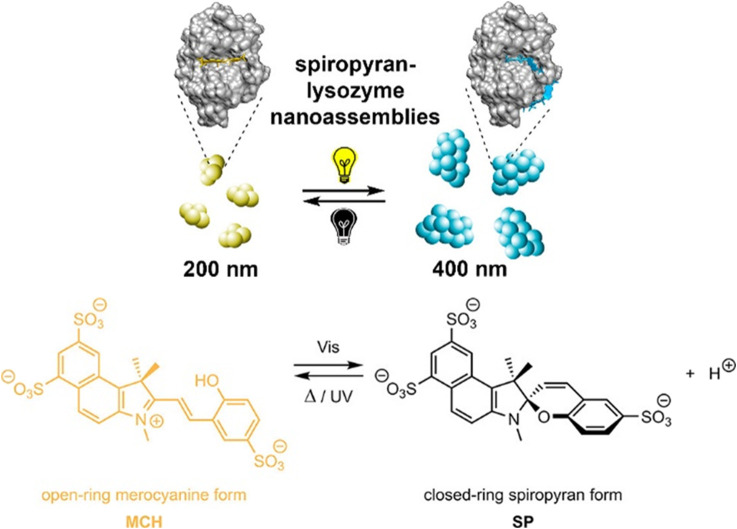
Light-triggerable enzyme aggregation and activity; top: Schematic representation of the assemblies of an ionic spiropyrane and lysozyme; bottom: Interconversion of the two spiropyran isomers in inverse photochromism (Moldenhauer et al., 2019). Reprinted with permission. Copyright ^©^ 2018 American Chemical Society.

Beyond classical *cis-trans* isomerization, other molecular switches that are also operated by light have elicited more attention in the recent past. While the usage of spiropyran in self-assembly systems is already well-established and well known (Ipe et al., 2003; Achilleos et al., 2012; Wang et al., 2015), it is not used that frequently in electrostatically self-assembled nanoparticles. The Gröhn group used the light-responsive property of spiropyrans to build supramolecular enzyme-spiropyran structures by electrostatic self-assembly (Moldenhauer et al., 2019). Here, they combined an anionic spiropyran sulfonate with the enzyme lysozyme. In the dark, the spiropyran is present in the thermodynamic stable open-ring merocyanine isomer (Figure 23). Upon irradiation, the merocyanine changes from the open-ring isomer to the closed-ring isomer, which thermally converts back in the dark or by UV irradiation. The ring closure changes the molecular structure, the number of charges, and the dipole moment, and thereby also the nanoscale assembly structure. While it decreases the basicity of the oxygen in the closed-ring isomer, the open-ring isomer has a much larger molecular volume. After mixing the building blocks in an aqueous solution, aggregates with a size of R_H_ = 103 nm form. Upon irradiation of merocyanine, the size nearly doubles to R_H_ = 198 nm, which changes back by storing the samples in the dark. This size-changing cycle can be repeated many times. The differences in size again, like in the azo dye-macroion system discussed above, can be understood with the different binding of the two spiropyran isomers to the protein. The merocyanine is planar and thus binds more internally to the protein, whereas the closed ring isomer binds more externally due to its bulky form and can so interconnect more lysozymes. However, irradiation not only influences the size of the aggregates but also the functionality of the enzyme. Interestingly, the enzyme lysozyme remains active in the assembly. The formation of the aggregates and the interaction with the merocyanine nearly does not change the enzymatic activity. After irradiation, this activity decreases significantly, which proves the concept of supramolecular structures where the enzymatic activity can be switched (Moldenhauer et al., 2019).

A photo-responsive *o*-nitrobenzyl linker that undergoes a photocleavage reaction was used in a different approach introduced by the group of Haag (Fischer et al., 2011). They synthesized a photo-responsive cationic dendritic poly (glycerol amine), which contains the *o*-nitrobenzyl linker. By combining this polyelectrolyte with DNA, nanostructures with sizes ranging from R_H_ = 25 nm to R_H_ = 40 nm depending on the ratio of positively-charged polymer amine groups to negatively-charged nucleic acid phosphate groups. The structures are formed by the strong binding between the anionic phosphate groups of the DNA with the cationic amines of the polyelectrolyte. They further demonstrated that upon UV irradiation the dendritic polymer is destroyed, which leads to the reduction of the multivalent electrostatic interaction between the DNA and the polyelectrolyte. Due to that the DNA could be released. In a similar approach, the group around Khashab et al. has used a photo-responsive *o*-nitrobenzyl linker (Li et al., 2015). They assembled anionic and cationic nitrophenylene-doped silica nanoparticles to form stable so-called colloidosomes. Upon irradiation, the photo-cleavage reaction of the nitrophenylene group leads to a charge reversal and a subsequent electrostatic repulsion, which leads to the disassembly of the formed nano assemblies. This system was then used for the controlled release of Nile Red dye upon irradiation.

**FIGURE 24:**
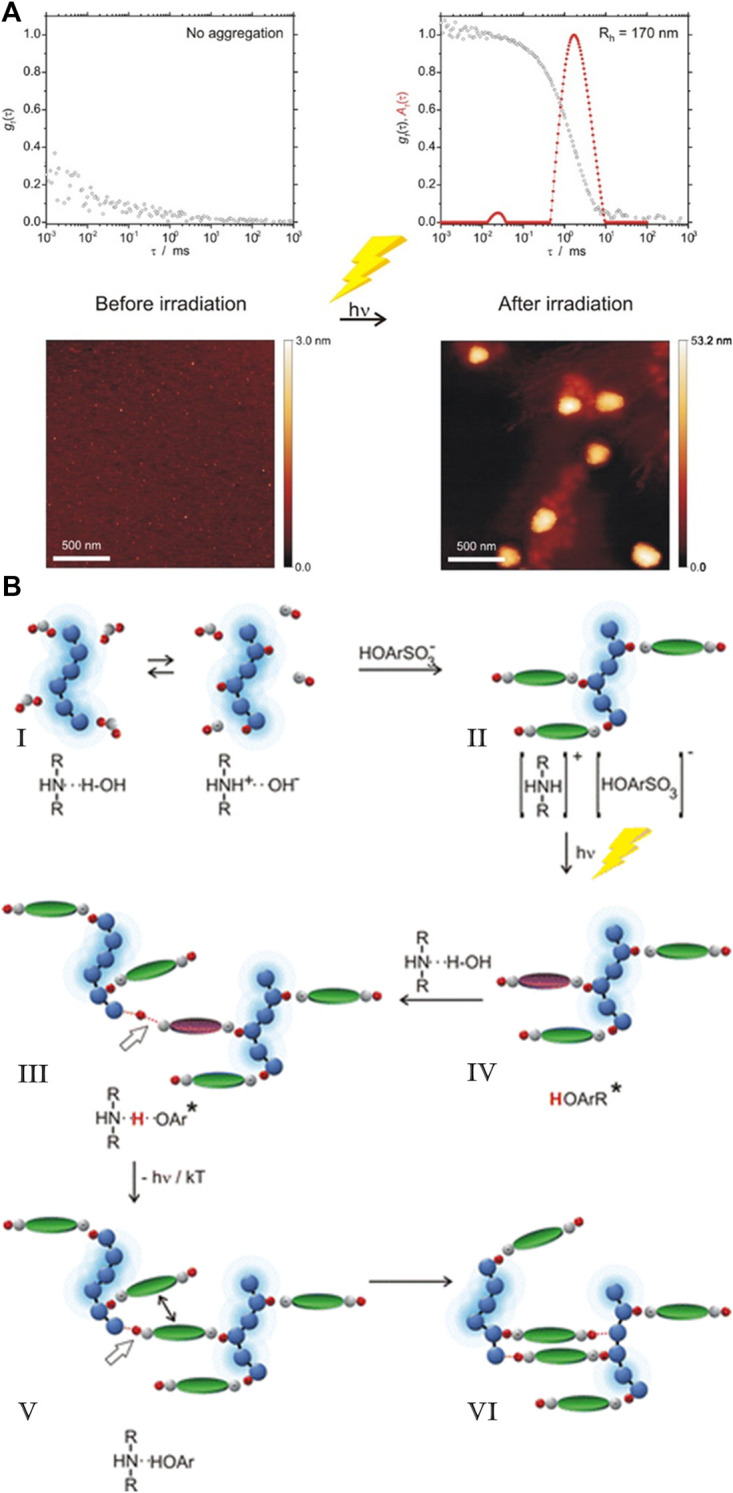
Photoacid which changes the pKa in the excited state acting as a building block in electrostatic self-assembly with linear oligo (ethylene imine): **A** DLS and AFM before **(left)** and after **(right)** UV irradiation. **B**: light-induced assembly mechanism (Cardenas-Daw and Gröhn, 2015). Reprinted with permission. Copyright ^©^ 2015 American Chemical Society.

In 2015, the Gröhn group demonstrated a completely different concept for building light-responsive nano-assemblies by using the unique properties of photoacids (Cardenas-Daw and Gröhn, 2015). Photoacids are only weak Brønsted acids in the ground state, and upon irradiation show an increase in acidity of the hydroxyl proton and undergo an excited-state intermolecular proton transfer reaction (Agmon et al., 2002). For this reason, a more highly charged molecule is formed. In the first project, a linear oligomeric ethylene imine and sodium 1-naphthol-4-sulfonate were combined. As the counterion only has one charge no assemblies are formed directly after mixing the building blocks. After irradiation DLS and AFM showed nano-assemblies with a size of R_H_ = 170 nm (Figure 24).

In combination with UV/Vis spectroscopy, a mechanism for the formation of the assemblies was proposed, as shown in Figure 24. First, before the irradiation, the addition of the counterion gives rise to electrostatic interaction between the cationic polyelectrolyte and the anionic naphthol. Upon irradiation, photoacid dissociation takes place, and the polyelectrolyte is protonated, which, in turn, triggers a strong interconnection due to electrostatic interaction. After decaying to the ground state, the interaction changes from the strong ionic interaction to hydrogen bonds. Due to the desolvation of the amine groups, a stronger hydrophobization of the polymer chains is to be expected. Thereafter, the building blocks are in closer approximation, which leads to rearrangements to take place due to hydrophobic, π-π, and electrostatic interactions. After this study, the Gröhn group extended the systems to develop a deeper understanding. Different photoacids were combined with G4 dendrimer (Zika et al., 2020). In this case, the photoacids have already two negative charges in the ground state. For this reason, nano-assemblies are already formed before irradiation. With the strongest photoacid, 1-naphthol-3,6-disulfonate nanostructures (1N36S) assemblies are formed with a size ranging between 89 nm ≤ R_H_ ≤ 190 nm (depending on loading ratio). Upon irradiation the size increases ranging to 130 nm ≤ R_H_ ≤ 244 nm (depending on loading ratio). Thus, the photoacid switch cannot only be used to trigger assembly from building blocks but also to alter the assembly size.

### 10.3 Further Triggers

Despite pH and light, other external influences are also used in electrostatic self-assembly, although more in singular examples only. Temperature is a well-known influence on the structure and shape of self-assembled structures in general. However, while it is the most effective switch in amphiphilic systems, for example, the effect in electrostatics often is less expressed and thus, less exploited. That is because a temperature shift of course changes the dynamics of the system, including the one of the hydration shell and thereby screening and interaction, while switches that directly access the charged nature and valency lead to more expressed changes. The group of Hao studied the capture and release of DNA by the protonation and deprotonation of alkyldimethylamine oxide in Tris-HCl buffer in dependence on temperature (Feng et al., 2016). At room temperature, the pH value is pH = 7.2 and the DNA molecules only weakly interact with the alkyldimethylamine oxide. Increasing the temperature causes an increase in the pH, which leads to the protonation of the alkyldimethylamine oxide and after electrostatic interaction with DNA.

Pedersen et al. studied a thermoresponsive system in dependence on added salt concentration, using two oppositely charged block copolymers namely poly (N-isopropylacrylamide)_48_-blockpoly ((3-acrylamidopropyl)trimethyl ammonium chloride)_20_ and poly (N-isopropylacrylamide)_70_-block-poly (4-styrenesulfonic acid sodium salt)_30_ (Fehér et al., 2019). With a ratio of polycation to polyanion of 1:1.8, the samples showed sufficient stability in a temperature range of 20–50°C and up to a salt concentration of 50 mM NaCl. Upon increasing the ionic strength at low temperature (below the lower critical solution temperature, LCST), the aggregation number of the micelles decreases, because the attractive electrostatic potential is screened. At a salt concentration of 100 mM NaCl the aggregation number increased again due to the decreasing solubility of the polycation. At a temperature above the LCST of the polycation, a strong association of the structures is observed at a high salt concentration. Here, the polycation loses its solubility in water and inverted micelles are formed.

A magnetic field as an external trigger for electrostatic assemblies was used by the group of Wuyi et al. (Liu F. et al., 2015). They used a positively charged hexadecyl trimethyl ammonium bromide, electropositive Fe_3_O_4_, TEOS, and negatively charged poly (styrene) as building blocks, which formed yolk-shell mesoporous silica nanospheres with a diameter of 600 nm. Upon exposure to an alternating magnetic field, the structure becomes more flocculent, and it is possible to release a drug *in vitro*.

Redox-responsive DNA-loaded vesicles were reported by Hao et al. (Liu et al., 2019). They combined lauric acid and the redox-active surfactant, (11-ferrocenylundecyl)trimethylammonium hydroxide where the overall charge of the surfactant can be changed from dicationic to mono-cationic upon oxidation and reduction of the ferrocene group. This leads to a shift of the ferrocene polarity from more hydrophilic to more hydrophobic. First, the two building blocks form uni- and multi-lamellar vesicles with a diameter of 21 nm. Oxidizing the ferrocene destroys the nano-assemblies and the nano-assemblies reform with the subsequent addition of a reductant, while the supramolecular structures undergo a redox-responsive rearrangement. Redox responsiveness significantly differs with DNA bound to the vesicles: With DNA, after adding an oxidant, the vesicles are still destroyed but the following addition of the reducing agent does not lead to a structure of micron-sized bilayer vesicle. A similar study with siRNA instead of DNA was also done (Chang et al., 2017).

### 10.4 Multiswitchability

Gröhn and coworkers were one of the first ones to combine two different external influences to achieve a multi-switchable system based on electrostatic self-assembly (Willerich and Gröhn, 2010). This is an extension of the light switchable system described above: The photo-responsive anionic azo dye Ay38 is combined with the pH-responsive cationic polyelectrolyte G4 dendrimer. For nano-assemblies sized at R_H_ = 32 nm and light irradiation leads to a size increase to R_H_ = 165 nm, as discussed above. These larger assemblies can now be dissolved by changing the pH value from pH = 3.5 to pH = 10.5. The increase in pH leads to the deprotonation of the amine groups of the polyelectrolyte and the assemblies dissolve as the macroion does not carry any charge. By keeping the assemblies in the dark, the dye can now more easily isomerize to its *trans*-state. After changing the pH back to pH = 3.5, the assemblies are reformed with the same size and form as in the beginning, i.e. a dually responsive system that can be triggered by pH and light has been built.

The Schmuck group introduced a dually responsive system, which combines pH responsiveness with chemical responsiveness (Gröger et al., 2011). They used two different binding interactions, the formation of an iron-terpyridine complex and the dimerization of the self-complementary zwitterion motif mentioned above. Thus, it is possible to switch the size and structure of the system between monomer structures, a metal-complexed dimer, and an ion paired dimer, and either cyclic or linear self-assembled aggregates. This can either be achieved by removing the metal and/or the addition of acid and base. Combining pH and chemical responsiveness was also focused on in other studies (Lin et al., 2015; Lei et al., 2019b).

Combining pH and temperature responsiveness, Lu et al. formed electrostatically self-assembled vesicle-like structures from poly (ionic liquid-co-N-isopropylacrylamide) with deoxycholic acid (Cui et al., 2012). Changing the temperature results in the change from vesicles to a rod-like structure and subsequently, to a micelle-like structure. The vesicles change into spherical micelles upon decreasing the pH, while also some precipitate is formed. This is probably due to the protonation of the zwitterion, which is then only a cation. Changing the pH back, the precipitate dissolves again and the vesicles are again formed. A similar study was conducted by Yu et al. (Gong et al., 2015). The group around Dong created a magnetic and light-responsive system from a cationic surfactant with a light-responsive azobenzene moiety in the tail and a paramagnetic counterion [FeCl_3_Br]^−^ (Xu et al., 2015). The cationic micelles formed were subsequently used to interact with and compact DNA. While the magnetic response leads to a higher degree of compaction efficiency, light irradiation results in the release of the DNA molecules.

**FIGURE 25:**
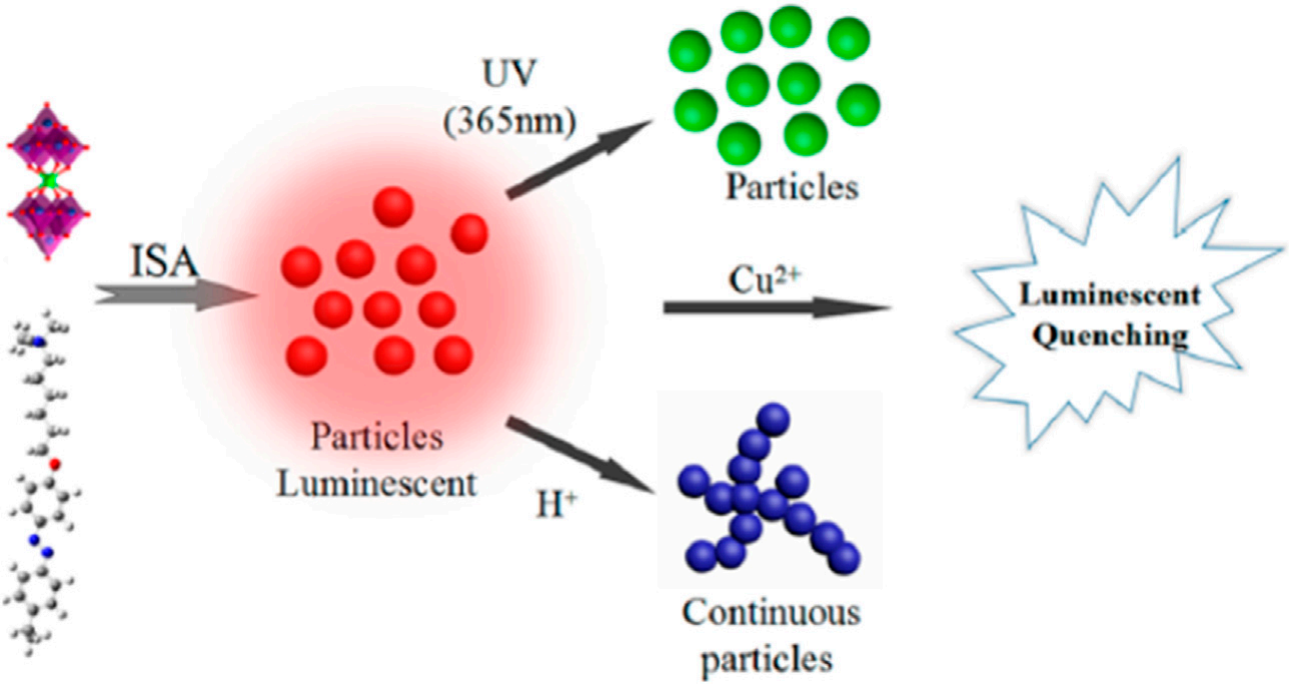
Formation of ETAB/Eu-POM supramolecular materials and their responsiveness to UV Light, Cu^2+^, and H^+^. (Guo et al., 2016b). Reprinted with permission. Copyright ^©^ 2016 American Chemical Society.

Remarkable is also a light-pH-copper ion tri-stimuli responsive system constructed by electrostatic self-assembly, as depicted in Figure 25 (Guo et al., 2016b). Yu et al. combined the cationic azobenzene-containing surfactant (4ethyl-4′-(trimethylaminohexyloxy) azobenzene bromide) with an anionic Eu-containing polyoxometalate, which resulted in a spherical aggregate structure with a size of d = 200 nm. After light irradiation, the particle size decreases somewhat, and the fluorescence of the POM is quenched strongly. This is probably attributed to the more hydrophilic nature around the luminescence center Eu^3+^. The *cis* isomer is more hydrophilic, which reduces the hydrophobic interaction, and it is sterically more hindered, which leads to a looser alignment. By adding H^+^, the morphology changes from a spherical structure to a necklace-like geometry, and the fluorescence is also quenched. The cause is a newly formed hydrogen bond W−O···H···O−H. Another way to quench the fluorescence of the assemblies is by adding Cu^2+^ with an energy transfer from the O→W ligand metal charge transfer excited state to the Cu^2+^ ion.

Electrostatically assembled supramolecular structures in form of vesicles and fibrils responsive to three different external influences namely temperature, pH change, and the solvent polarity were investigated by the Sarkar group (Dutta et al., 2019). They used a surfactant with merocyanine units. At basic pH, both the fiber and vesicle structures reveal a dendritic growth upon increasing the temperature, which the authors explain by an increase in “hydrophobic interactions”. By tuning the solvent polarity, it is possible to hinder the formation of aggregates.

The combination of the ferrocenyl surfactant with a dye molecule is a well-studied topic with great variety and potential (Sui et al., 2019; Sui et al., 2020; Yu et al., 2021). For example, electrostatic self-assembly of a 16-alkyl (ferrocenyl-methyl)ammonium bromide and the anionic alizarin red resulted in rod-like nano-objects (Sui et al., 2019). Due to the high electron density and the spacing, the ferrocene group can further form stable inclusion complexes when β-cyclodextrin is added. Upon inclusion of the hydrophobic ferrocene into the cyclodextrin, the structure of the assembly changes to a dendritic morphology due to the change in hydrophilicity of the head group. Assemblies also are redox-responsive: In the oxidized form, the ferrocene is more hydrophilic, which leads to the formation of vesicles. Moreover, it is also possible to change the fluorescence: In the assembly, the fluorescence of the dye is strongly dependent on the concentration of the ferrocene suggesting that ferrocene quenches the fluorescence. After adding an oxidant, the fluorescence increases significantly, while the subsequent addition of a reducing agent leads to the quenching again. A possible cause is that the oxidation of the ferrocene reduces its electron density and the electron transfer from the head group to the photo-excited state of the alizarin red is suppressed and thus the fluorescence increases. Moreover, the dye molecules carry hydroxyl groups that can respond to a pH change: Next to a change in the color of the solution the morphology changes from a rod-like structure at pH = 6.0 to a star-like structure at pH = 2.0. When the pH value is changed to pH = 10.0, a peony-like structure is built.

**FIGURE 26:**
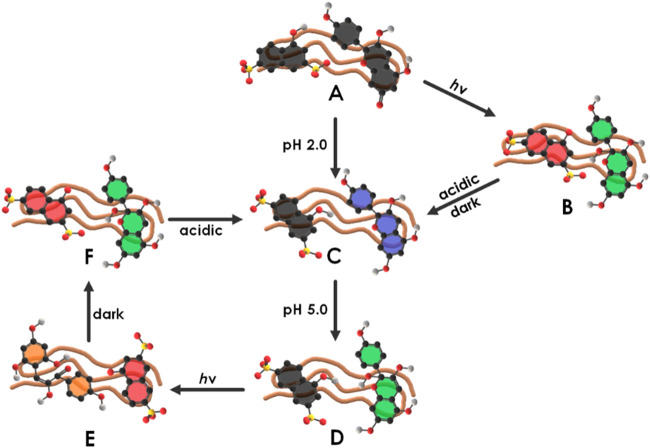
A ternary multiswitchable assembly formed of Flavy, a photoacid (1N36S) and linear poly (allylamine) (simplified schematic representation displaying one molecule of each component per assembly only). **(A)**: The protonated photoacid and Flavy in its closed form. **(B)**: The deprotonated photoacid and hydroxylated Flavy. **(C)**: The protonated photoacid and protonated Flavy. **(D)**: The protonated photoacid and hydroxylated Flavy. **(E)**: The deprotonated photoacid and open Flavy. **(F)**: The deprotonated photoacid and hydroxylated Flavy (Zika and Gröhn, 2021). The Figure was reproduced, © 2021 A. Zika and F. Gröhn, distributed under the terms of the Creative Commons Attribution 4.0 International License.

Recently, Zika and Gröhn built a ternary (three-component) pH- and light-responsive system by the association of a photoacid — the anionic disulfonated naphthol derivative 1N36S –, neutral hydroxyflavylium ions (Flavy) and a linear cationic poly (allylamine) in an aqueous solution (Zika and Gröhn, 2021). Hydroxyflavylium can exhibit a network of different reactions depending on pH and light irradiation. Due to the unique light-responsive property of the photoacid and the pH and light-dependent hydroxyflavylium, this system offers multiple ways of triggering the nanostructures (Figure 26). The ternary electrostatic self-assembly of the building blocks leads to assemblies with a size of R_H_ = 192 nm. Depending on the switching step, the size of the assembly ranges between R_H_ = 142 nm and R_H_ = 332 nm. In addition, it is possible to switch both the shape and size of the nano-objects. While triggering the hydroxyflavylium does not change the spherical structure, excitation of the photoacid leads to the formation of disc-like nanoparticles. Similar to the electrostatically formed systems discussed above, the stability of the nano-assemblies in this case also depends on the surface charge density. Moreover, the shape and size of the assemblies can also be adjusted by the component ratio.

## 11 Electrostatic Nano-Objects as Supramolecular Photocatalysts

One advantage of electrostatic self-assembly is the huge amount of possible building block combinations. By choosing catalytically active building blocks, for example, porphyrins or polyoxometalates (POM), synergetic effects with other building blocks can lead to an improvement of the catalytic activity. The positive effects can range from geometric effects such as structural proximity and high surface area and charge over advantageous changes in electron density to complementary effects like an electron donor-acceptor relation between the building blocks or the implementation of a sensitizer.

**FIGURE 27:**
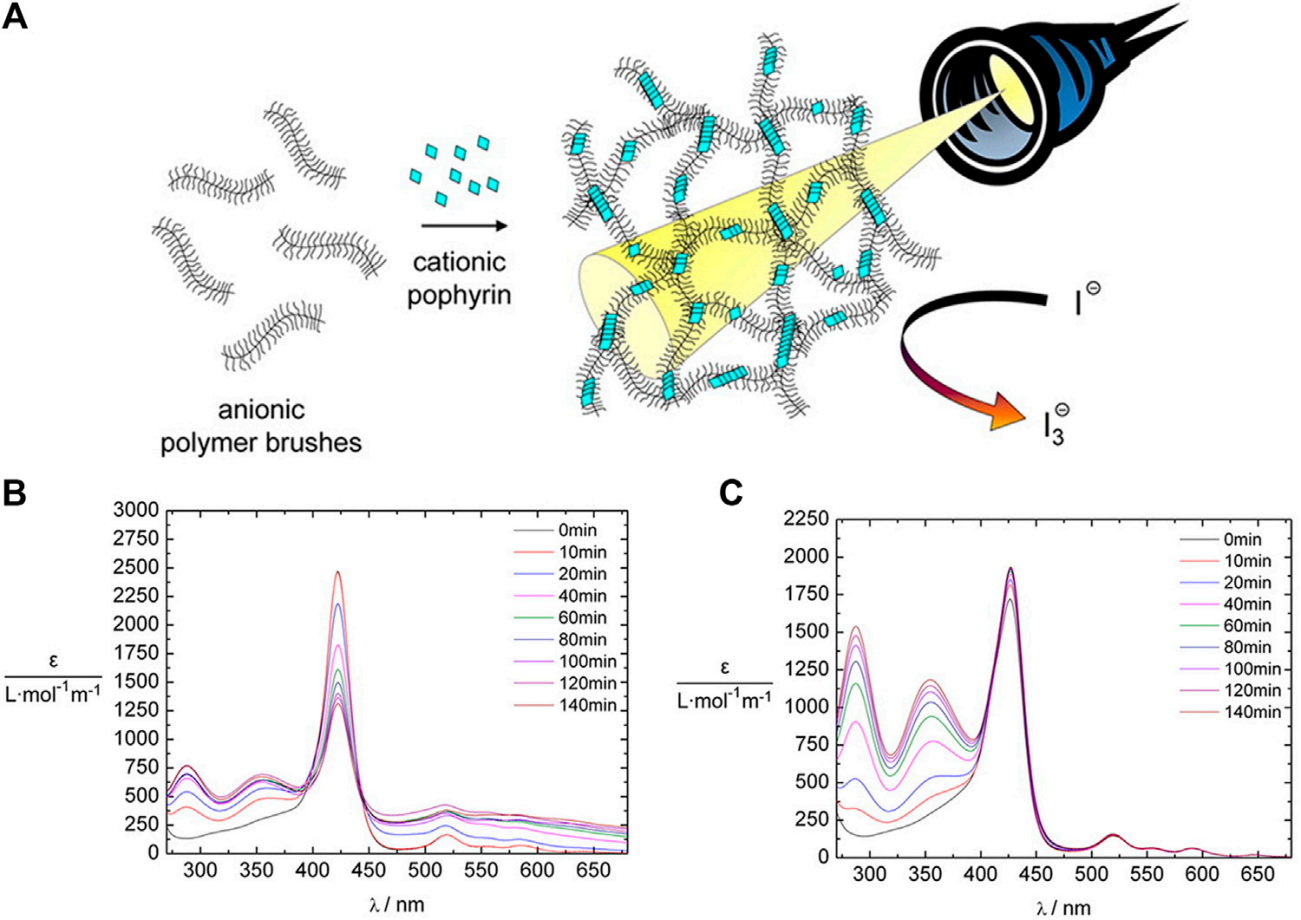
Polyelectrolyte brush-porphyrin assemblies as photocatalysts: **(A)** Scheme, and UV/Vis absorption spectra in dependence on irradiation time for catalyzing the iodide oxidation as the model reaction: **(B)** TMPyP without polyelectrolyte and **(C)** a TMPyP/PSS brush sample; The bands at λ = 353 nm and at λ = 287 nm indicate the faster tri-iodide formation with the nano-assembly as the catalyst (Frühbeißer and Gröhn, 2012). Reprinted with permission. Copyright ^©^ 2012 American Chemical Society.

A proof of concept was presented by the Gröhn group, who showed that poly (styrene sulfonate) brush-TMPyP network assemblies sized at 40 nm ≤ R_H_ ≤ 160 nm (see also section Structural Variety) are stable in an aqueous solution and can photo-oxidize iodide to triiodide, as a model reaction for photocatalysis (Frühbeißer and Gröhn, 2012). The catalytic activity is up to eight times higher than for the pure porphyrins as shown in Figure 27, and in following variations of the systems could be enhanced up to 22-fold (Frühbeißer et al., 2016).

The catalytic activity is improved through the association with the polymer since in an aqueous solution only the porphyrin forms larger aggregates of micrometer size with triiodide during the reaction, thereby limiting its catalytic activity. In contrast, in the polymer-brush-porphyrin assemblies, the porphyrin is bound in a defined manner to the polymer and unwanted aggregation is hindered, preserving its accessibility and catalytic activity. While the solution without the polymer even showed slightly faster oxidation at the very beginning of the reaction, the polymer-porphyrin assemblies enhance the catalytic performance over time. In contrast, the addition of the stryrene sulfonate monomer to the porphyrin did not change the catalytic activity. This demonstrates that the macroion-porphyrin structure formation plays an important role in the catalytic activity. Additionally, further cationic porphyrins are combined with poly (styrene sulfonate) polymer brushes to study the catalytic activity (Frühbeißer et al., 2016). The size of the resulting network-like structures and their photocatalytic activity also depend on the porphyrin used. While the TMPyP diacid shows an enhancement in catalytic activity after adding the polyelectrolyte at a charge ratio of 0.35 ≤ l ≤ 0.6, tetrakis (4-(trimethylammonium)phenyl) porphyrin (TAPP) diacid, Zn-TMPyP, and TMPyP monoacid show only a slight increase. TMPyP diacid-PSS brush assemblies exhibit a 22-times larger triiodide generation. Given that the lifetime of the triplet state and molecular symmetry can be excluded as possible reasons for this behavior, the aggregation of the porphyrin must play is a key factor for the activity. The difference in electronic structure causes the different behavior of TAPP diacid and TMPyP diacid in catalytic activity, as can be analyzed by UV/Vis spectroscopy.

**FIGURE 28:**
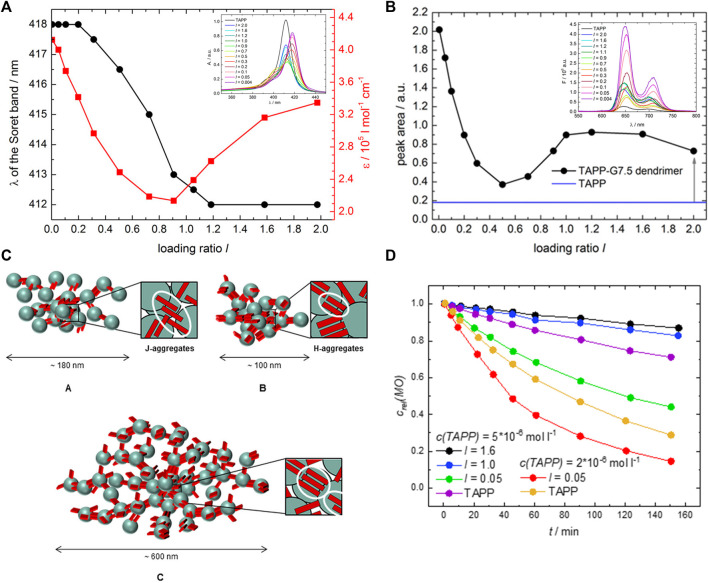
TAPP-G4 dendrimer aggregates and the dependence of the catalytic activity on the internal stacking of the porphyrins: **(A)** UV/Vis analysis in dependence on the loading ratio l: shift of the Soret band and changes in the extinction coefficient ε of the Soret band at pH 11; inset: Soret band; **(B)** fluorescence analysis in dependence on the loading ratio l: area of the fluorescence peak at 574 nm ≤ λ ≤ 800 nm; inset: fluorescence; **(C)** Schematic illustration of TAPP−G7.5 dendrimer assemblies with *l* = 0.05, *l* = 1.0, and *l* = 1.6 (red: TAPP molecules; blue: G7.5 dendrimers); **(D)** photocatalytic degradation of methyl orange upon irradiation with visible light similar to the sun spectrum as a photocatalytic model reaction: Decrease of the methyl orange concentration measured at λ = 464 nm for TAPP−G7.5 dendrimer assemblies with different loading ratios and for TAPP only (Krieger et al., 2017). Reprinted with permission. Copyright ^©^ 2017 American Chemical Society.

Other cases of photocatalytically active polyelectrolyte-porphyrin assemblies include aggregates of cationic TAPP and anionic PAMAM dendrimer of generation 7.5 at basic pH (Figure 28), as investigated by Krieger and Gröhn (Krieger et al., 2017) Here as well, the size of the cylindrical aggregates and the internal aggregation of the porphyrins into stacks within the assemblies depends on the charge ratio, as depicted in Figure 28. The shift in both UV/Vis spectra and fluorescence spectra demonstrates the internal stacking of the porphyrins in the assemblies. This has a direct effect on photocatalytic activities (Figure 28D). In excess of dendrimer (*l* = 0.05), the porphyrins build J-aggregates, which show a higher quantum yield due to a smaller HOMO-LUMO gap and have a length of 180 nm, whereas H-aggregates are predominate and have a length of 100 and 500 nm, respectively at equal charge stoichiometry (*l* = 1.0) and an excess of porphyrin (*l* = 1.6). The photocatalytic degradation of methyl orange with visible light increases for dendrimer-porphyrin assemblies with *l* = 0.05 in comparison to pure TAPP, whereas aggregates with a charge ratio of *l* = 1.0 and *l* = 1.6 show a lower activity. The increased photocatalytic activity for the assemblies with excessive dendrimer is ascribed to two reasons: the large surface area due to the small particle size, and the higher number of porphyrin J-aggregates. Thus, both the internal assembly structure and the size of the assemblies are accountable for the methyl orange’s degradation rate.

Porphyrin-surfactant assemblies of meso-tetra(4-carboxyphenyl)porphyrin and cetyltrimethylammonium bromide exhibit shapes from spherical to ellipsoids to flakes and finally, to flowers, as was shown by Mandal et al. (Mandal et al., 2014). Rhodamine B is photocatalytically degraded by the different morphologies. 56, 81, 79, and 71% of the Rhodamine B are degraded by the spherical, the ellipsoids, the flakes, and the flowers, respectively. Thus, morphology also plays an important role in the catalytic activity in surfactant-based structures. Similar to the dendrimer-porphyrin particles, the aggregation of the porphyrin in these structures is decisive for the catalytic activity and J-aggregates enhance the photodegradation. Moreover, other Zn-porphyrin-surfactant particles are capable of photocatalyzing the hydrogen evolution with Pt as the cocatalyst (Zhong et al., 2019).

Electrostatically self-assembled organic-inorganic hybrid systems with metal nanoparticles as a catalyst are of promise as well (Biffis et al., 2003; Krieger et al., 2020). The self-assembled organic building blocks serve as a template for the nanoparticles. Bioinspired assembly of a Zn-protoporphyrin and human serum albumin can reduce water with triethanolamine as sacrificial methylviologen in the capacity of a mediator. Here, the porphyrin-protein assemblies act as a photosensitizer, without which the Pt-particles cannot form H_2_. The efficiency of H_2_ evolution was 25–30% higher for the porphyrin-protein complex than for samples without the protein (Komatsu et al., 2006).

Peptide-porphyrin assemblies with Pt-nanoparticles can serve as biomimics for light-to-chemical energy conversion (Kim et al., 2012; Yang et al., 2020). Light-harvesting peptide nanotubes were synthesized by the interaction between diphenylalanine, and tetra (phydroxyphenyl) porphyrin via electrostatic self-assembly, which contains attached Pt-nanoparticles by the group of Park (Kim et al., 2012). The porphyrins enabled the self-metalation process of the Pt-nanoparticles which serve as electron separators and transfer the electrons efficiently to nicotinamide. The nicotinamide cofactors, coupled with an enzymatic model reaction, are regenerated. This system mimics the light reaction of photosynthesis. For α-ketoglutarate to L-glutamate conversion, the efficiency of the nanotube was about 48 times higher as compared to porphyrin alone. With similar building blocks, peptide–porphyrin assemblies with a self-mineralized reaction center of TiO can mimic the light-harvesting of primitive photobacteria (Liu K. et al., 2016).

A supramolecular so-called multi-to-one photocatalyst has been assembled by multiple graphene quantum dots on a unimolecular porphyrin micelle with Pt as central atom, a so-called single Pt-site porphyrin (Ji Y. et al., 2021). The latter ones form micelle-like structures by themselves with large, positively charged side groups of the porphyrin as head groups. A core-shell structure forms by adding the anionic graphene quantum dots, with a single porphyrin in the core and multiple graphene quantum dots in the corona. These nano-assemblies are photocatalysts for water splitting with ascorbic acid as the sacrificial agent. The hydrogen evolution rate is nearly 200 times higher than for the individual building blocks, which highlights the proficiency of the multi to one light energy transfer. Several graphene quantum dots transfer energy to a single porphyrin and excite it, thus generating electron-hole pairs. The non-aggregated Pt-ions in the center of the porphyrin utilizes the catalytic site and boost the photocatalytic activity. Although a slight decrease in the hydrogen evolution rate is observed after three cycles, the nano-assemblies remain more stable than Pt-site porphyrin.

**FIGURE 29:**
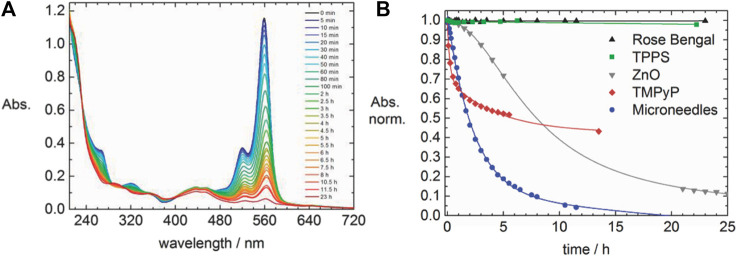
Photodegradation of Rose Bengal by porphyrin-porphyrin microrhombuses decorated with ZnO nanorods: **(A)** Time-dependent UV/Vis spectroscopy of the degradation of Rose Bengal with the ZnO microrhombuses. **(B)** Catalytic degradation of Rose Bengal with ZnO-microrhombuses (blue circles), with TMPyP (red rhombs), with TPPS (green squares), with ZnO-nanorods (gray inverted triangles), and without catalyst (black triangles) (Düring et al., 2017b). Reprinted with permission. Copyright ^©^ 2017 WILEY-VCH Verlag GmbH and Co. KGaA, Weinheim.

Furthermore, hybrid systems of ZnO nanorods with two oppositely charged porphyrins have been investigated (Düring et al., 2017b; Düring et al., 2018). The aggregation of cationic TMPyP and anionic TPPS leads to the formation of a rhombus-like microneedles with a size of 3.5 µm in length and 1.5 µm in broadness, which were still stable in solution. These assemblies decorated with ZnO are highly promising for photocatalysis, as demonstrated by the degradation of the dye Rose Bengal shown in Figure 29. The degradation is more effective with the microneedles as compared to the single components. A comparison of the photodegradation of different dyes showed that degradation was only possible for anionic dyes containing either carboxyl or hydroxyl groups. The high selectivity for carboxyl and hydroxyl groups indicates that either TMPyP or ZnO is responsible for the degradation process, while TPPS acts only as a light-harvester. The selectivity for the dye degradation arises from a highly positive ζ-potential of the rhombuses.

Phthalocyanines can be used instead of porphyrin as a photocatalytic active building block to form functional self-assemblies. For example, nanovesicles with a size of R_H_ = 250 nm and a wall thickness of 62.5 nm are built by phthalocyanine and amphiphilic amino acids 9-fluorenylmethyloxycarbonyl-l-histidine (Han et al., 2019). The photocatalytic activity was demonstrated by the reaction of dopamine to leucodopaminechrome. The production rate of the vesicle is twice as high as the phthalocyanine alone due to an increase in the formation of photoexcited states.

Beyond porphyrins, also other dyes can play significant roles in photocatalysis. Polyelectrolyte-dye assemblies can constitute an effective photocatalyst with the dye as photosensitizer. Since the pioneering work of Meijer et al. on amphiphilic dendrimers acting as a “box”, dendrimer- Rose Bengal assemblies are well-known (Jansen et al., 1994). A recent study of a Rose Bengal-G4 PAMAM dendrimer system deals with photocatalysts (Kutz and Gröhn, 2017). In this case, the reduction of methyl viologen at different pH values ranging from pH = 3.5 to pH = 10.5 is used as a model reaction. At pH = 8.0 the reduction was nearly seven times higher than for the dye alone. The enhanced conversion rate can be attributed to the macroion, which reduces the distance between the Rose Bengal molecules. This replicates the dimer formation at a high dye concentration. Due to electrostatic interaction, the dye molecule binds at the periphery and interior of the cationic dendrimer to form dimers with increased catalytic activity. Furthermore, the aggregation of methyl viologen with the dendrimer inhibits an electron back transfer to Rose Bengal. Results also revealed a pH-dependent assembly structure and photocatalytic activity. At pH = 3.5, the dendrimer is highly charged and the monovalent Rose Bengal counterions distribute inside and around the dendrimer macroion, without leading to interconnection as is the case for multivalent dye counterions. At pH = 10.5, larger aggregates with a size of R_H_ = 37 nm are found, in difference to systems discussed in the last sections and although the dendrimer is uncharged. It was shown that hydrogen bonding of the Rose Bengal–OH groups and halogen bonding of its Cl-groups occurs. The photocatalytic activity increases from pH = 3.5 to pH = 8.0 and decreases at even higher pH values again. Evidently, a strong interaction of the components disables the catalytic reduction of methyl viologen again. Although the formation of dimers is important, it seems crucial to maintain an equilibrium of dimer and monomer. For the first time, assembly formation was shifted from predominantly electrostatic interactions at low pH to hydrogen and halogen bonding at higher pH in this study. A delicate interplay of all three types of interactions — electrostatic interaction, hydrogen and halogen bonding — may be a further origin of the optimized performance. Creating a pH-dependent efficiency of the assemblies based on the variation of the underlying non-covalent interaction forces may develop into a general concept for tunable catalytic activity in future. While this will require a — so far missing — quantitative understanding of the interaction interplay, an (understood) pH-dependent activity is highly desirable in photodynamic cancer therapy and in solar energy conversion.

Another type of promising complex self-assembled organic structures, which are photocatalytically active, comprises electrostatically self-assembled water-soluble pillar[5]arene, a bola-type salicylaldehyde-azine derivative, and two different hydrophobic fluorescent dyes Eosin Y and Nile Red can transfer solar energy into chemical energy through the photocatalytic dehalogenation reaction of α-bromoacetophenone in an aqueous phase (Sun et al., 2021).

Polyoxometalates (POMs), consisting of several transition metal oxyanions, exhibit excellent catalytic properties and carry multiple charges, which makes them ideal as building blocks for electrostatically self-assembled catalysts. For example, catalytically active sandwich-type POMs, in conjunction with the chiral amphiphilic cation bis(1-phenylethyl)amine hydrochloride, build spherical structures sized at R_H_ = 50 nm, where the chiral building blocks introduce a chirality to the assemblies (Wang et al., 2012). Thus, the oxidation of prochiral asymmetric sulfide as methyl phenyl sulfide with the addition of H_2_O_2_ is possible and the catalytic activity was up to 72% enantiomeric excess due to the chiral microenvironment of the chiral organic cations surrounding POMs. Meanwhile, the assembly is also an effective catalyst for asymmetric sulfoxidation.

The POM Na_7_PW_11_O_39_ immobilized on quaternary ammonium functionalizes chloromethylated polystyrene oxidized alcohols (Wang et al., 2016). The electrostatically self-assembled material is less efficient compared to pure POM but has the advantage of recyclability. Supramolecular nanowires are constructed from surfactant-encapsulating polyoxometalates (Zhou et al., 2016). However, these structures were not stable in solution and started to aggregate in an aqueous solution. By loading the aggregates with Ag-nanoparticles, 4-nitrophenol (4-NP) is reduced in the presence of NaBH_4_. Moreover, other POM-surfactant materials are known as heterogeneous catalysts (Sun et al., 2018; Ji X. Y. et al., 2021).

Sun et al. used Na_9_ [EuW_10_O_36_]·32H_2_O in combination with a cationic peptide which forms spheres of R_H_ = 285 nm but starts to precipitate after a few days (Tong et al., 2017). The assemblies degrade methylene blue upon irradiation with visible light and H_2_O_2_ as a sacrificial molecule. The decomposition efficiency is 91%, while neither of the components or binary component combinations can decompose the dye at all. This is because the electrostatic interaction with the peptide exposes the photocatalytic active sites of POM; at the same time, the POM molecule accepts electrons from the peptide. Furthermore, the assemblies immobilize and stabilize the POM by protecting it from degradation. The same POM Na_9_ [EuW_10_O_36_]·32H_2_O is combined with biomolecule dopamine and catalyzes the degradation of methyl orange and rhodamine B with the help of H_2_O_2_ (Zhang et al., 2017). Also here, the spherical nanoflowers formed started to precipitate after a few days. Again, the POM-dopamine-assemblies were highly catalytic active in degrading methyl orange in comparison to the building blocks.

**FIGURE 30:**
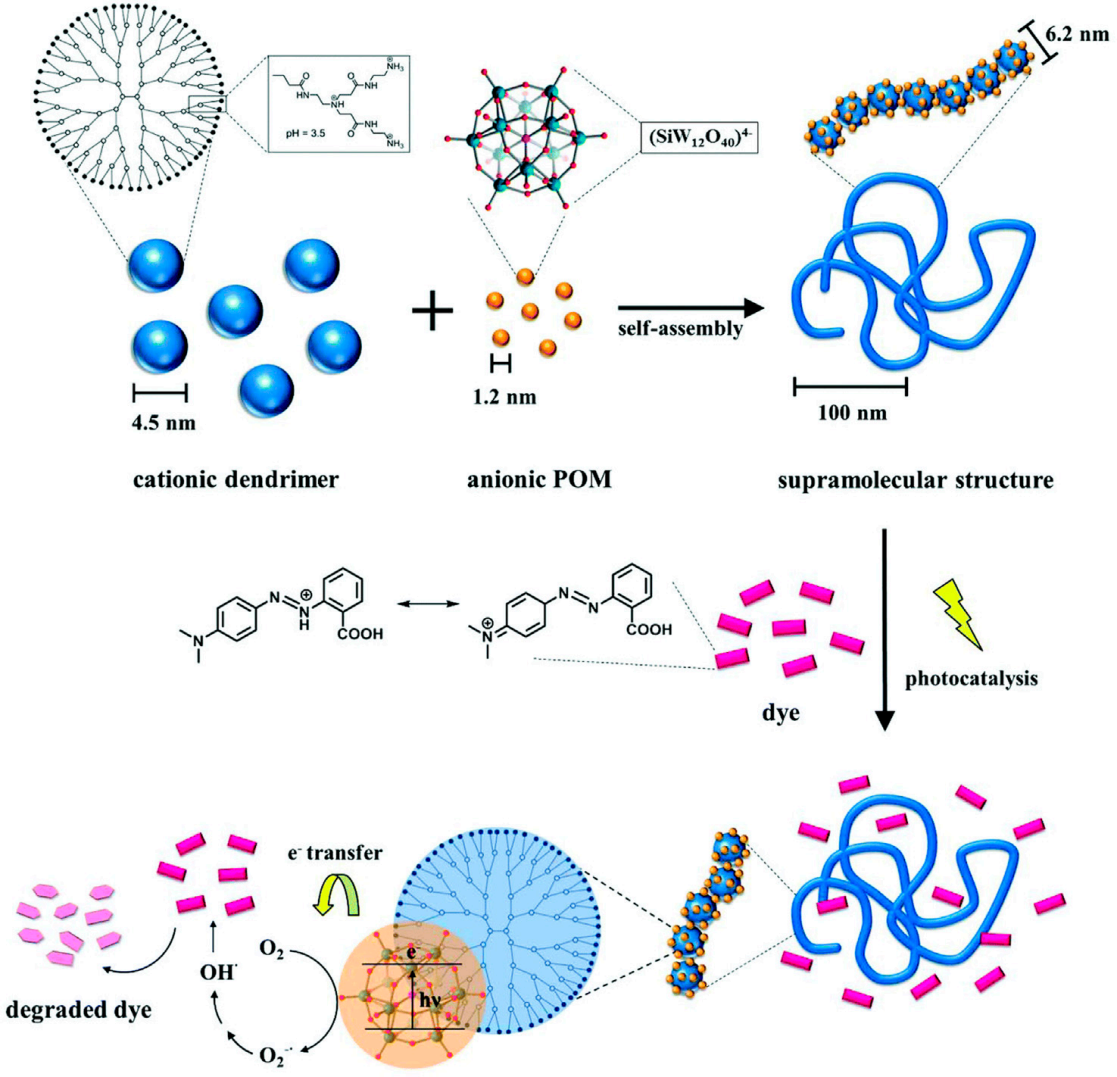
POM–dendrimer self-assembly and photocatalysis in aqueous solution as investigated: **(A)** Scheme and photos of the photocatalytic degradation of methyl red by POM–dendrimer assemblies and POM in aqueous solution; A: POM–dendrimer before irradiation, **(B)**: POM–dendrimer after 20 min UV-irradiation, **(C)**: POM before irradiation, **(D)**: POM after 20 min UV-irradiation; The dye degrades much more with the POM–dendrimer assembly (A → B) as compared to the cluster only solution (C → D) (Kutz et al., 2018). Reprinted with permission. Copyright ^©^ The Royal Society of Chemistry 2018.

Self-assembled structures based on POMs with long-term stability in water at pH = 3.5 have recently been introduced by the Gröhn group (Kutz et al., 2018). Nanostructures of electrostatically assembled anionic Keggin-type polyoxometalate cluster K_4_ [SiW_12_O_40_] and cationic G4 dendrimer, as already mentioned in the section on Structural Variety, have been shown to represent selective photocatalysts for dye degradation. The photocatalytic activity was tested for a variety of cationic and anionic dye molecules with very different structures. In the case of the cationic methyl red, the assemblies degrade the dye completely in 20 min, while pure POM only degrades 11% since the cationic dye aggregates with the anionic POM, as UV/Vis data in Figure 30 reveal. The photo-degradation of other cationic dyes such as methylene blue behaves similarly. In contrast, the photocatalytic degradation of anionic dyes such as xylenol orange is significantly faster by pure POM clusters rather than with the assemblies. Thus, the system exhibits a charge selectivity in the degradation which might initially seem surprising in its direction, as one might expect that a cationic dye should react well with the anionic POM — without the need for a cationic polyelectrolyte that could diminish their approach. UV/Vis spectroscopy indeed revealed the attachment of the cationic methylene blue to the anionic POM clusters in absence of polyelectrolyte, but the attachment is so strong and complete that the POM surface becomes passivated. With the polyelectrolyte, in contrast, the POM surface is kept accessible.

The trivalent Keggin anion K_3_PW_12_O_40_ and the Keggin anion H_4_SiW_12_O_40_, generate network-like structures in combination with a cationic carboxylic functionalized poly (ionic liquid) by electrostatic self-assembly, which acts as heterogeneous catalyst for the additive-free Baeyer-Villiger oxidation of cyclic ketones (Li X. et al., 2020). H_3_PW_12_O_40_-assemblies show a higher catalytic activity than H_4_SiW_12_O_40_-assemblies but both ionic-liquid-POM assemblies show higher conversion with H_2_O_2_, selectivity, and yield than the POM alone in solution. This is attributed to two reasons. Firstly, the more highly charged H_4_SiW_12_O_40_ leads to a higher acidity content in the self-assembly process, which makes it easier for the lactone to be hydrolyzed. Furthermore, the higher charge enhances the cross-linking of the nano-assemblies, which decreases the number of pores on the surface and as a result, the active surface area. Secondly, H_3_PW_12_O_40_ has a higher oxidative activity. To test the generality of the principle, various ketone substrates were tested where all were oxidized with one exception, possibly pointing to an interesting though not yet fully understood selectivity. The reusability of the catalyst was also demonstrated, as no efficiency loss occurred after five reactions.

Thus, already a couple of different photocatalytically active nano-objects have been formed by electrostatic self-assembly, with very promising properties as activity enhancement and selectivity. Evidently, many questions are still open in this young field, and a fundamental understanding of the structure-function relationships will have to be extended to exploit the full potential of these types of structures.

## 12 Conclusion

In conclusion, the design of functional nano-objects by electrostatic self-assembly in solution represents an emerging field with great potential. For a long time, electrostatic interaction had not been significantly exploited as much as the hydrophobic effect or hydrogen bonding for the creation of defined self-assembled structures. Reasons have been the complex interplay of interactions in a multi-component assembly-building bock-counterion system on the one hand, and the spherical symmetric electrostatic potential of an electric charge, seemingly “boringly” non-directional as opposed to directional amphiphilic and hydrogen bonding motifs on the other hand. Only the targeted combination of electrostatic interaction with other effects and interactions, such as the positioning of charges on stiff building blocks, the use of additional amphiphilic, π-π stacking building blocks, or polyelectrolytes with certain architectures, have promoted electrostatic self-assembly to a principle for versatile defined structure formation.

After structured materials consisting of polyelectrolyte-surfactant or polyelectrolyte-dye combinations by Thünemann and Antonietti, and Faul and Antonietti, respectively, about 20–25 years ago, respectively, the formation of defined assemblies in a solution has come into focus only more recently. A classical case is represented by assemblies comprising double-hydrophilic block polyelectrolytes with a charged and a neutral block together with a homo-polyelectrolyte or with another oppositely charged block polyelectrolyte, as investigated by Cohen Stuart and others. Here, to some extent, design concepts can be transferred from micelle formation. Since 2009, the formation of nano-objects by two water-soluble building blocks, mostly a polyelectrolyte with multivalent stiff counterions, has been established by the Gröhn group. In recent years, it has been elucidated as to how the interplaying interactions control the size and shape of the nano-assemblies, where association thermodynamics play a key role. On the one hand, the free energy — additively composed of the attractive contributions such as electrostatic and π-π interaction — encodes the aggregation number and the entropy/enthalpy balance sets the degree of particle anisotropy on the nanoscale; on the other hand, a molecular property, specifically the polar surface area of the π-π stacking dye counterions, was shown to be the molecular factor again determining the association thermodynamics. A variety of nano-objects with shapes of spheres, rods, capsules, chains, networks, flexible fibers, core-shell structures and others has been developed in this manner.

The first steps toward functionality have been undertaken. In this review, we focused on two main aspects: switchability and photocatalytic activity. Triggering assembling/disassembling, nano-object size, and nano-object shape have been presented. While pH responsiveness is more straightforward in this context, various approaches to light responsivity have also been discussed, ranging from a triggerable size and shape in azo-dye-based systems, to spiropyran linked enzymes with addressable activity, to the construction with photoacids as an addressable linker in electrostatically self-assembled nano-objects. As photoacids exhibit a different degree of dissociation in the excited state, the ionic valency can be triggered via light irradiation. Moreover, the recent findings on magnetic switching and the response of optical properties in electrostatic self-assembly have been addressed.

Very promising potential lies in the just emerging field of photocatalytically active assemblies. As discussed herein, the photocatalytic activity of porphyrins and polyoxometalates can be enhanced up a factor of 20 by electrostatic self-assembly with polyelectrolytes, and more importantly, a novel selectivity can be achieved. By choosing these catalytically active building blocks, synergetic effects with other building blocks lead to an improvement of the catalytic activity. The positive effects can range from geometric effects like structural proximity over advantageous changes in electron density to complementary effects such as an electron donor-acceptor relation between the building entities.

A major key to really establishing electrostatic self-assembly, or novel self-assembly approaches in general, in new areas — such as in this case for the formation of functional and switchable nano-assemblies — is developing a fundamental understanding of interplaying interactions and structure and property directing effects. This review’s authors opine that the time of just “throwing together” some entities that then form “some” interesting structures are being increasingly taken over from detailed studies on fundamental effects guiding the structuring. Naturally, an understanding has so far not reached the levels of, for example, micelle formation being investigated for more than 60 years by several researchers focusing on different aspects. Nevertheless, such understanding of the complex interplay in electrostatic self-assembly is both needed and in the process of being developed. It is only with this approach that a targeted design of functional structures will be possible, and only then will it be possible to exploit the great capability of electrostatic self-assembly that represents a general concept and makes it possible to form an immense variety of structures and functions for various applications.
